# Natural variation of the wheat root exudate metabolome and its influence on biological nitrification inhibition activity

**DOI:** 10.1111/pbi.70248

**Published:** 2025-07-21

**Authors:** Arindam Ghatak, Alexandros E. Kanellopoulos, Cristina López‐Hidalgo, Andrea Malits, Yuhang Meng, Florian Schindler, Shuang Zhang, Jiahang Li, Steffen Waldherr, Hugo Ribeiro, Melina Kerou, Logan H. Hodgskiss, Maximilian Dreer, Reyazul Rouf Mir, Sandeep Sharma, Gert Bachmann, Dimitrios G. Karpouzas, Christa Schleper, Evangelia S. Papadopoulou, Palak Chaturvedi, Wolfram Weckwerth

**Affiliations:** ^1^ Molecular Systems Biology Lab (MOSYS), Department of Functional and Evolutionary Ecology University of Vienna Vienna Austria; ^2^ Vienna Metabolomics Center (VIME) University of Vienna Vienna Austria; ^3^ Laboratory of Plant and Environmental Biotechnology, Department of Biochemistry and Biotechnology University of Thessaly Larissa Greece; ^4^ Archaea Biology and Ecogenomics Unit, Department of Functional and Evolutionary Ecology, Faculty of Life Sciences University of Vienna Vienna Austria; ^5^ Division of Genetics & Plant Breeding, Faculty of Agriculture (FoA) SKUAST‐Kashmir Srinagar India; ^6^ Centre for Crop and Food Innovation (CCFI) Murdoch University Murdoch Western Australia Australia; ^7^ Department of Genetics and Plant Breeding, Institute of Agricultural Sciences Banaras Hindu University Varanasi India; ^8^ Laboratory of Environmental Microbiology and Virology, Department of Environmental Sciences University of Thessaly Larissa Greece; ^9^ Environment and Climate Research Hub University of Vienna Vienna Austria; ^10^ Research Network Health in Society University of Vienna Vienna Austria

**Keywords:** Nature‐based Solution (NbS), root exudates, *Triticum aestivum*, root exudate metabolome, Biological Nitrification Inhibition (BNI), fast‐track BNI screening bioassay

## Abstract

Excessive nitrogen use and low nitrogen use efficiency (NUE) in current agroecosystems are disrupting the global nitrogen cycle. Chemical inhibitors offer only temporary relief, while plant‐derived biological nitrification inhibitors (BNIs) remain safer but underexplored. Identifying biological nitrification inhibition (BNI) traits in nitrogen‐demanding crops like wheat is key to improving sustainability. In this study, a combined GC‐ and LC–MS platform was used to determine the metabolome of the root exudates of 44 diverse wheat genotypes originating from India and Austria. With more than 6000 metabolic features, a pronounced genotype‐specific variation, a clear geographic pattern and an unexpected complexity of the root exudate metabolome were observed. A novel high‐throughput assay utilizing diverse ammonia‐oxidizing bacteria (AOB) and archaea (AOA) was developed for rapid BNI testing, highlighting distinct inhibition and even growth stimulation capacities between genotypes. Network analysis and advanced machine and deep learning analysis identified combinations of 32 metabolites linked to high BNI activity, including phenylpropanoids sinapinic acid, syringic acid and others, as well as glycosylated flavones isoschaftoside and others. This indicates that the concurrent presence of specific metabolites, rather than a single compound, drives nitrification inhibition in the rhizosphere. Variation in BNI activity among wheat genotypes, classified as either spring or winter types, suggests that root architecture modulates the dynamics of root exudation and the potential for nitrification inhibition. The unique combination of high‐throughput metabolomics analysis and the BNI fast‐track assay allows for screening of large germplasm collections as an essential requirement to introduce BNI and related NUE traits into modern breeding programmes.

## Introduction

Feeding a rapidly growing global population sustainably poses significant challenges. By 2050, agricultural production worldwide is projected to increase by 70% in order to provide adequate food and nutrition for 9 billion people. Nitrogen (N), a crucial macronutrient, constitutes 1–5% of the total dry weight of plants. As a result, agriculture heavily depends on nitrogen fertilizers to maximize crop yields. However, fertilizer N is a ‘double‐edged sword’ that ensures food security for much of humanity, while having enormous negative impacts on the environment and human health (Sutton *et al*., 2019). Globally, it is also the single largest source of nitrous oxide (N_2_O), a potent (~300 times the global warming potential of carbon dioxide) and long‐lived greenhouse gas (Winiwarter *et al*., 2018). Thus, in many agricultural systems, too much N is used and there is a need to rein in the amounts of N fertilizer used to reduce environmental pollution (Weckwerth *et al*., 2025).

Currently, one potential strategy for reducing nitrogen (N) losses involves curbing the nitrification process through the application of synthetic nitrification inhibitors (SNIs). These include compounds such as dicyandiamide (DCD), 3,4‐dimethylpyrazole phosphate (DMPP) and 2‐chloro‐6‐(trichloromethyl) pyridine (nitrapyrin). These inhibitors can extend the retention time of ammonium N in the soil, which increases nitrogen use efficiency (NUE). However, SNIs can have several disadvantages, including high synthesis costs and negative effects on soil health. For example, commercially available SNIs can be toxic to non‐target microbes in the soil, leading to unintended changes in soil functions (Bachtsevani *et al*., 2021; Ghatak *et al*., 2023). Additionally, their performance can be inconsistent across different soils and microbiomes; while they effectively reduce the growth of ammonia‐oxidizing bacteria (AOB), they have limited effectiveness in inhibiting the growth of ammonia‐oxidizing archaea (AOA) (Beeckman *et al*., 2018; McGeough *et al*., 2016; Papadopoulou *et al*., 2020; Wolt, 2004).

Interestingly, biological nitrification inhibitors (BNIs) demonstrate a promising option to alleviate N losses by the nitrification process (Ghatak *et al*., 2024). The phenomenon termed ‘biological nitrification inhibition (BNI)’ refers to the natural ability of some plants to suppress soil nitrification by exuding secondary metabolites from roots (Ghatak *et al*., 2023; Subbarao *et al*., 2015). Therefore, a key step towards the implementation of BNIs in agricultural practice is understanding the role of root exudates and their associated metabolomes in regulating soil N fluxes. The root exudates of Brachiaria grasses have demonstrated the highest BNI capacity, but sorghum (*Sorghum bicolor*) is at the forefront among crops in terms of BNI‐production capacity (Subbarao *et al*., 2009, 2013).

BNIs are exuded into the rhizosphere, a site which is usually the most populated with ammonia‐oxidizing microorganisms of both groups (AOB and AOA) compared to the bulk soils (Nardi *et al*., 2020), and both of which could be affected by BNIs (Byrnes *et al*., 2017; Lan *et al*., 2022; Lu *et al*., 2019; Nardi *et al*., 2013). In this context, current research is focused on identifying a variety of BNI‐positive wheat plants and metabolites to promote the sustainable management of nitrogen (Nardi *et al*., 2020). However, most studies have focused on identifying single, novel plant metabolites with potential nitrification inhibition capacity, often ignoring the concurrent presence of multiple metabolites with the same or maybe complementary inhibition potency in the root exudates of BNI‐positive plants (Egenolf *et al*., 2020; Otaka *et al*., 2022). Another limitation in BNI research is the screening systems used for the discovery of potent BNI compounds and plants. This was long based on testing of plant extracts on low‐throughput and time‐consuming axenic cultures of ammonia‐oxidizing microorganisms (AOM) (Sun *et al*., 2016), or on fast‐track screening assays with a genetically modified strain of a single AOB species, *Nitrosomonas europaea* (Iizumi *et al*., 1998; Subbarao *et al*., 2006), of low ecological relevance in soil ecosystems. Recent studies developed high‐throughput testing platforms enriched with soil‐relevant AOM strains (i.e. AOB *Nitrosospira multiformis* and AOA *Nitrososphaera viennensis*) (Beeckman *et al*., 2023; O'Sullivan *et al*., 2016) but still provide a limited coverage of the AOB and AOA diversity in soil, especially of AOA, which exhibit varying sensitivities to NIs (Kaur‐Bhambra *et al*., 2022; Kolovou *et al*., 2023b; Papadopoulou *et al*., 2020), distinct ecophysiological diversity and in many cases dominate AOB in soil nitrification (Huang *et al*., 2021). Hence, despite the several fast‐track screening methods available, we are still in demand of methods that provide a broader coverage of the functional diversity of AOM in soil ecosystems that enable ecologically relevant and early detection of the potential nitrification inhibitory activity of chemicals or extracts.

Wheat (*Triticum aestivum*) is the primary crop consumed worldwide, with production expected to reach 3.8 Mg/ha by 2050 (Alexandratos and Bruinsma, 2012; Roychowdhury *et al*., 2024). For production, wheat agro‐systems are managed with intensive N fertilization, but nitrogen use efficiency is only about 33% (Raun and Johnson, 1999; Tilman *et al*., 2002). Additionally, modern wheat cultivars do not exhibit detectable BNI capacity in their root systems (Subbarao *et al*., 2007a). As a result, they require the application of slow‐release SNIs or improved N management practices to mitigate nitrogen pollution (Dawar *et al*., 2021; Matson *et al*., 1998). Research by Subbarao and colleagues has indicated that *Leymus racemosus*, a wild relative of wheat, possesses a high BNI capacity, with the responsible genes located on chromosome Lr#n (Lr#n‐SA = T3BL.3NsbS) controlling BNI capacity (Subbarao *et al*., 2007b). Based on these findings, new wheat cultivars with enhanced BNI capacity in their root systems have recently been released, specifically ‘ROELFS‐BNI’ and ‘MUNAL‐BNI’ (Subbarao *et al*., 2021). Field studies using isogenic lines of MUNAL (i.e. MUNAL‐BNI vs MUNAL‐Control) in slightly acidic soils (pH 6.0) have shown significant improvements in grain yields across a range of N inputs. Furthermore, nitrification rates and N_2_O emissions were significantly lower (about 30%) in areas where MUNAL‐BNI was grown (Subbarao *et al*., 2021). The development of these new BNI‐producing wheat lines represents a major milestone towards greater sustainability in agricultural systems. However, we are still missing systematic and fundamental knowledge on the mechanism and metabolites underpinning the BNI activity of wheat lines, especially the combinatory effect of root exudates on AOB and AOA growth. Consequently, screening wheat genotypes for their root exudate metabolome and verifying in parallel their BNI‐like potential through advanced testing against a range of phylogenetically and ecophysiologically distinct AOM can enrich wheat germplasm with more BNI capacity, act as a thesaurus for biologically active plant metabolites and provide a better understanding of the metabolic pathways employed by plants to regulate soil N.

The current study aimed to evaluate the potential for BNI in 44 diverse wheat genotypes sourced from Austria and India and identify individual or groups of metabolites that may be involved in inhibition activity. To achieve these objectives, root exudates were collected from the wheat genotypes and analysed in two primary ways: (a) through metabolomic analysis, which focused on identifying primary and secondary metabolites using gas chromatography (GC) and liquid chromatography–mass spectrometry (LC–MS), and (b) by assessing BNI activity using a novel high‐throughput testing platform developed for this study. This platform included a set of ecologically relevant and ecophysiologically diverse strains of AOB and AOA. The data gathered from the metabolomic analysis and BNI capacity were subjected to advanced statistical and machine and deep learning techniques. These methods led to the identification of metabolites that showed individual or combined effects on BNI activity. This integrative approach aims to enhance the accuracy of predictions for specific traits, such as BNI capacity, which is crucial for future breeding programmes.

## Results

### Genotype‐specific changes in the exudation of primary and secondary metabolites reveal a pronounced natural variation

**Figure 1:**
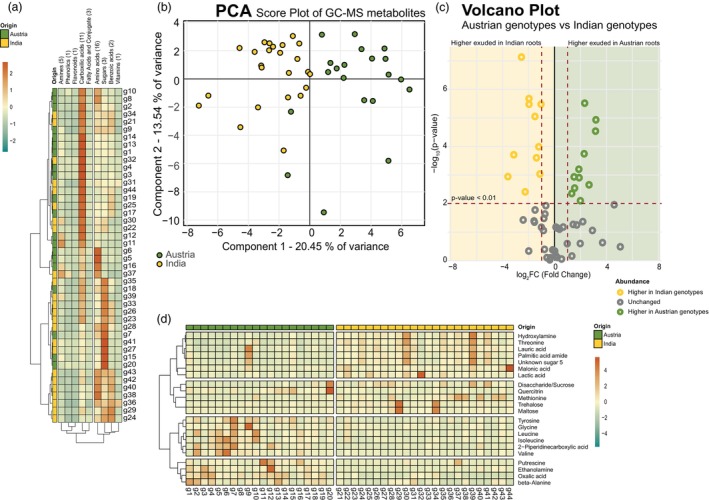
GC–MS metabolomics analysis of root exudates. (a) Heat map. The heat map highlights accumulation pattern of the metabolites identified in the root exudates of different genotypes. The genotypes and the chemical class of the metabolites were clustered using Pearson correlation. Metabolic changes are presented as the mean of each chemical class and are scaled by the sum. The values inside brackets indicate the number of compounds in each chemical class. Colours indicate regulation of the chemical class, increased levels (red) and decreased levels (green). (b) Principal component analysis. PCA Score plot of metabolites identified in the root exudates. (c) Volcano plot. A volcano plot was generated with a 44 genotype dataset (20 Austrian genotypes and 24 Indian genotypes and 56 identified primary metabolites in the root exudates). Green circles represent metabolites with a fold change ≥2 that were statistically significant (*P* ≤ 0.01), indicating higher accumulation in Austrian genotypes compared to Indian genotypes. Yellow circles represent metabolites with a fold change ≤ −2 that was statistically significant (*P* ≤ 0.01), indicating higher accumulation in Indian genotypes compared to Austrian genotypes. Grey circles represent metabolites with a fold change ≤2 to ≥ −2 and lack statistical significance (*P* > 0.01). (d) Heat map. The heat map highlights accumulation pattern of the metabolites identified in the root exudates of different genotypes. The metabolites were clustered using Pearson correlation. Metabolic changes were presented as the mean of each metabolite and scaled by the sum. Colours indicate regulation of the chemical class, increased levels (red) and decreased levels (green).

**Figure 2:**
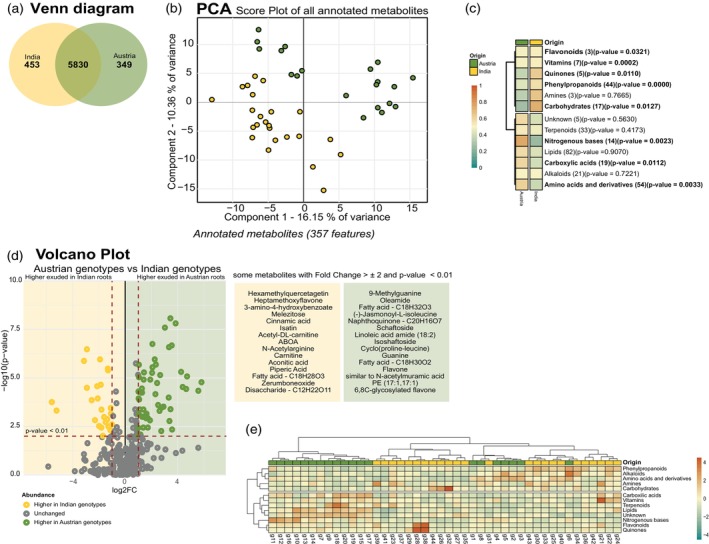
LC–MS metabolomics analysis of root exudates. (a) Venn diagram. All the detected features were used in the Venn diagram to distribute metabolic features between Austrian and Indian genotypes. (b) Principal Component Analysis considering annotated features (Annotation levels 1–4). PCA Score plot. (c) Heat map. Accumulation pattern of the metabolites in the root exudates of different genotypes. The genotypes and the chemical class of the metabolites were clustered using Pearson correlation. The chemical classes were clustered using Pearson correlation. Metabolic changes are presented as the mean of each chemical class and are scaled by the sum. The values inside brackets indicate the number of compounds in each chemical class. The bold chemical class indicates statistical significance (*P* ≤0 0.05). Colours indicate regulation of the chemical class, increased levels (red) and decreased levels (green). (d) Volcano plot. A volcano plot was generated with a 44 genotype dataset (20 Austrian and 24 Indian genotypes), considering 357 annotated features in root exudates. Green circles represent metabolites with a fold change ≥2 that was statistically significant (*P* ≤ 0.01), indicating higher accumulation in Austrian genotypes than in Indian genotypes. Yellow circles represent metabolites with a fold change ≤ −2 that was statistically significant (*P* ≤ 0.01), indicating higher accumulation in Indian genotypes than in Austrian genotypes. Grey circles represent metabolites with a fold change ≤2 to ≥ −2 and lack statistical significance (*P* > 0.01). The boxes indicate the names of the highlighted annotated features. (e) Heat map. Highlights accumulation pattern of the metabolites identified in the root exudates of different genotypes. The genotype and chemical class were clustered using Pearson correlation. Metabolic changes are presented as the mean of each chemical class and are scaled by the sum. Colours indicate regulation of the chemical class, increased levels (red) and decreased levels (green).

Metabolome profiling of the root exudates was conducted using GC‐time‐of‐flight (TOF)‐MS and high‐resolution LC–MS (Figure 1). Metabolites from GC–MS analysis were identified and quantified using quality control (QC) mixes and in‐house libraries (Chaturvedi *et al*., 2024a, 2024b; Ghatak *et al*., 2022; Zhang *et al*., 2021) and classified into organic acids, amino acids, sugars and amines (Figure 1a; Table S1a; Figure S1a,b). Principal component analysis (PCA) revealed distinct metabolites associated with Austrian and Indian wheat genotypes (Figure 1b), suggesting a clear geographic distinction in metabolite accumulation. The loadings of principal component 1 (PC1) revealed a unique set of metabolites; the positive loadings include metabolites such as valine, 2‐piperidine‐carboxylic acid, ethanolamine and oxalic acid, which showed enhanced accumulation in Austrian wheat genotypes, whereas negative loadings include metabolites such as hydroxylamine, palmitic acid, threonine and lauric acid, showing higher accumulation in Indian wheat genotypes (Figure S1c, Table S1c). Metabolites with significant accumulation (*P* <0.01) were determined by a volcano plot (Figure 1c, Table S1d), including hydroxylamine, malonic acid, lactic acid, threonine and unknown sugar 5 in Indian wheat genotypes and oxalic acid, 2‐piperidine‐carboxylic acid, ethanolamine, valine, putrescine, leucine and tyrosine in Austrian wheat genotypes. A heatmap represents the regulation of these significant metabolites across all the genotypes (Figure 1d). Using LC–MS analysis, 6632 m/z metabolic features were detected in total with 357 high quality annotations (Figure 2; Table S2a). To generate a broad survey of identified m/z features with altered regulation in the compared genotypes, a Venn analysis was prepared; 453 m/z features were found unique in the Indian wheat genotypes, and 349 m/z features were unique to the Austrian wheat genotypes (Figure 2a, Table S2b). Principal component analysis of annotated features showed a clear separation between the compared wheat genotypes (Figure 2b). The loadings of principal component 1 (PC1) revealed a unique set of metabolites; the positive loadings include metabolites such as fatty acids, flavons and nucleotide bases, which showed enhanced accumulation in Austrian wheat genotypes, whereas negative loadings include metabolites such as di‐ and trisaccharides and cinnamic acid, showing higher accumulation in Indian wheat genotypes (Figure S2d,e, Table S2c,d). The annotated features were classified into flavonoids, vitamins, quinones, phenylpropanoids, amines, carbohydrates, terpenoids, nitrogenous bases, lipids, carboxylic acids, alkaloids and amino acids and derivatives (Table S2a; Figure 2c). Metabolites with significant accumulation (*P* <0.01) were determined by a volcano plot (Figure 2d; Table S2d), including isatin, cinnamic acid, ABOA and carnitine in Indian wheat genotypes and oleamide, schaftoside, isoschaftoside and guanine in Austrian wheat genotypes (Figure 2d). A heatmap represents the regulation of the chemical class across all the genotypes (Figure 2e).

### Fast‐track system validation and application to wheat root exudates BNI screening

**Figure 3:**
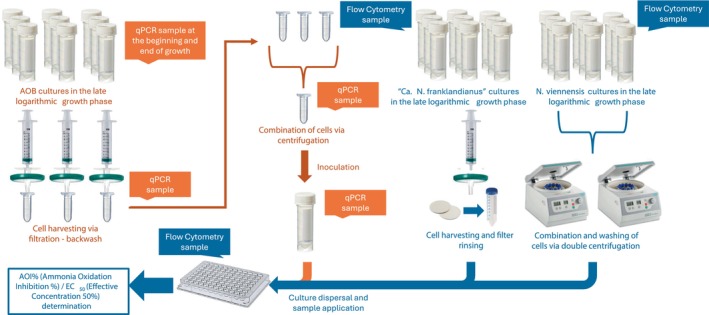
Overview of the development of the fast‐track, high‐throughput system. The AOB workflow is presented in orange, while the AOA workflow is shown in blue. The points of DNA sample collection for qPCR during the procedure specifically refer to the standardization step.

In order to test the root exudates for BNI activity, we developed a fast‐track BNI assay with different AOA and AOB strains (Figure 3). Cell concentration procedures were implemented to obtain significantly increased and stable cell densities (Appendix S1—Table S3 and Figures S1–S3) and consistently increased activity rates (Appendix S1—Table S4 and Figures S1–S6) of AOM strains compared to routine cultures. Inhibition thresholds for different known inhibitors revealed differences in efficacy and strain sensitivity (Appendix S1—Table S5 and Figures S7, S8), consistent with literature (Appendix S1—Table S5 and Figures S21–S23). A full account of the fast‐track system validation results is provided in the Appendix S1.

**Figure 4:**
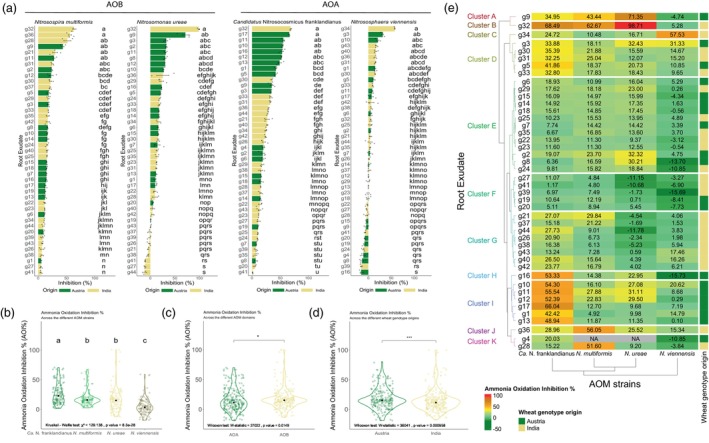
Fast‐track wheat root exudate screening. (a) AOM responses to root exudates. Bar plots showing AOM strain responses to the wheat root exudates ranked by inhibitory activity. Points indicate individual ammonia oxidation inhibition values, with error bars representing standard error. Bars are coloured by the origin of wheat genotypes, according to the bottom legend. Lowercase letters indicate groupings based on Kruskal–Wallis tests. (b–d) Summary of BNI variability of root exudates. Violin plots showing ammonia oxidation inhibition (AOI%) variation by (b) AOM strain, (c) AOM domain and (d) Origin of wheat genotypes. Black points indicate the group mean value. Lowercase letters indicate Kruskal–Wallis groupings (*P* < 0.05, groups with different lowercase letters differ significantly, while shared letters indicate no significant difference between groups). Asterisks denote Wilcoxon test significance (ns, not significant, *P < 0.05, **P < 0.01 and ***P < 0.001). Statistics appear in the bottom left of each graph. (e) Hierarchical clustering of root exudate BNI capacity. Heatmap of mean AOI% values for each root exudate (RE) across four reporter AOM strains. Box colours reflect AOI% values, as indicated by the legend. Wheat genotype origins are annotated on the right, with corresponding colours.

A strain‐specific response was observed when screening the ammonia oxidation inhibition by wheat root exudates, combined with a wheat genotype‐specific effect (Figure 4). Genotype g32 was the most inhibitory against three AOM reporter strains (Figure 4a), but did not significantly inhibit *N. viennensis* (NV), which was mainly inhibited by root exudates of g34 (Figure 4a). Overall, *Ca.* N. franklandianus (NF) was the most sensitive strain to the root exudates tested, followed by AOB, with *N. viennensis* being the least sensitive AOM strain (Figure 4b). AOB were slightly more sensitive compared to AOA (W‐statistic = 27 022, *P* <0.05) (Figure 4c), and root exudates from Austrian genotypes exhibited significantly higher potency than those from Indian genotypes (W‐statistic = 36 041, *P <*0.001) (Figure 4d).

Hierarchical clustering revealed distinct inhibitory patterns grouping root exudates into several clusters (Figure 4e). Notable clusters were A and B, where g32 and g9 were the most potent inhibitors and Cluster C, where g34 primarily inhibited AOA (Figure 4e). Clusters D, H and I encompass root exudates of Austrian genotypes, with significant inhibitory activity on *Ca.* N. franklandianus, while Clusters J and K featured g36 and g28 with targeted inhibitory activity on *N. multiformis* (NM) (Figure 4e). Clusters E and F consisted of root exudates with low inhibitory potential (Figure 4e). Principal Component Analysis (PCA) reaffirmed g9, g32 and g34 as the most potent root exudates and distinctly separated root exudates targeting AOB (g9, g28, g36) or AOA (g34, g3, g10, g17), explaining 78.6% of the variation (Appendix S1—Figure S24).

### Identification of metabolites with high inhibitory potential on AOM

**Figure 5:**
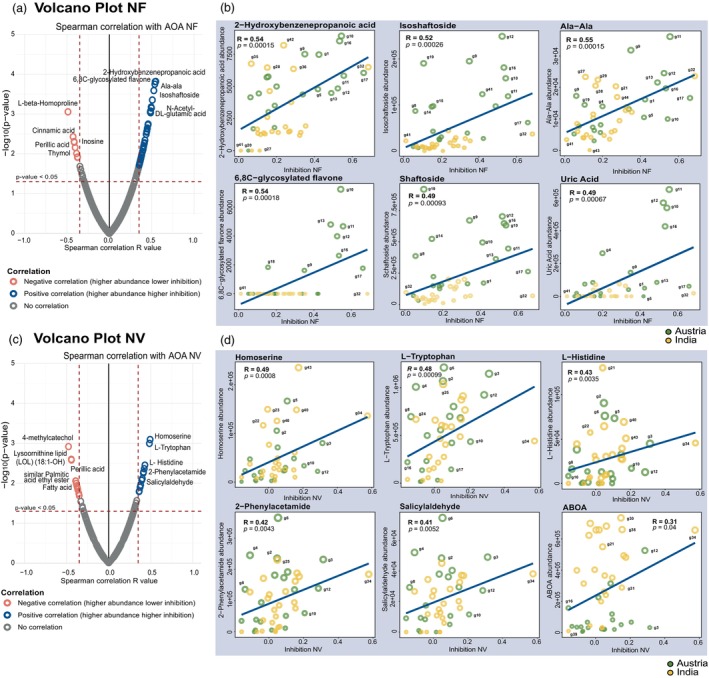
Inhibition of AOA strains by metabolites. Spearman correlation analysis was used to distribute 44 diverse wheat genotypes, considering metabolite abundance in root exudate (identified using LC–MS) and the inhibition of AOA strains. Pairwise correlations were calculated between the 357 annotated metabolite levels and AOA inhibition. (a) Volcano plot of NF inhibition. A volcano plot was generated using 357 annotated metabolite levels identified in root exudates and NF inhibition data. Blue circles represent metabolites with a Spearman's correlation *R*‐value higher than 0.40, which is statistically significant (*P* ≤0.05), indicating a positive correlation with NF inhibition. Red circles represent metabolites with a Spearman's correlation *R*‐value lower than −0.45, which is statistically significant (*P* ≤ 0.05), indicating a negative correlation with NF inhibition. Grey circles represent metabolites with a Spearman's correlation *R*‐value ≤0.40 to ≥ −0.45 and lacking statistical significance (*P* > 0.05). (b) NF inhibition distribution plot. Significantly correlated metabolites and NF inhibition activities were detected by Spearman's correlation analysis (*P* <0.05). Dots were colour‐coded according to the origin of the genotype. The size of each circle denotes the relative amount of metabolites corresponding to the wheat genotypes. Only some of the highly significant positively correlated metabolites are shown. (c) Volcano plot of NV inhibition. A volcano plot was generated using 357 annotated metabolite levels identified in root exudates and NV inhibition data. Blue circles represent metabolites with a Spearman's correlation *R*‐value higher than 0.40, which was statistically significant (*P* ≤ 0.05), indicating a positive correlation with NV inhibition. Red circles represent metabolites with a Spearman's correlation *R*‐value lower than −0.40, which were statistically significant (*P* ≤ 0.05), indicating a negative correlation with NV inhibition. Grey circles represent metabolites with Spearman's correlation *R*‐value ≤0.40 to ≥ −0.40 and lacking statistical significance (*P* > 0.05). (d) NV inhibition distribution plots. Significantly correlated metabolites and NV inhibition activities were detected using the Spearman's correlation approach (*P* < 0.05). Dots were colour‐coded according to the origin of the genotype. The size of each circle denotes the relative amount of metabolites relative to the corresponding wheat genotypes. Only some of the highly significant positively correlated metabolites are shown. NF (*Ca.*Nitrosocosmicus franklandianus). NV (*Nitrososphaera viennensis*).

**Figure 6:**
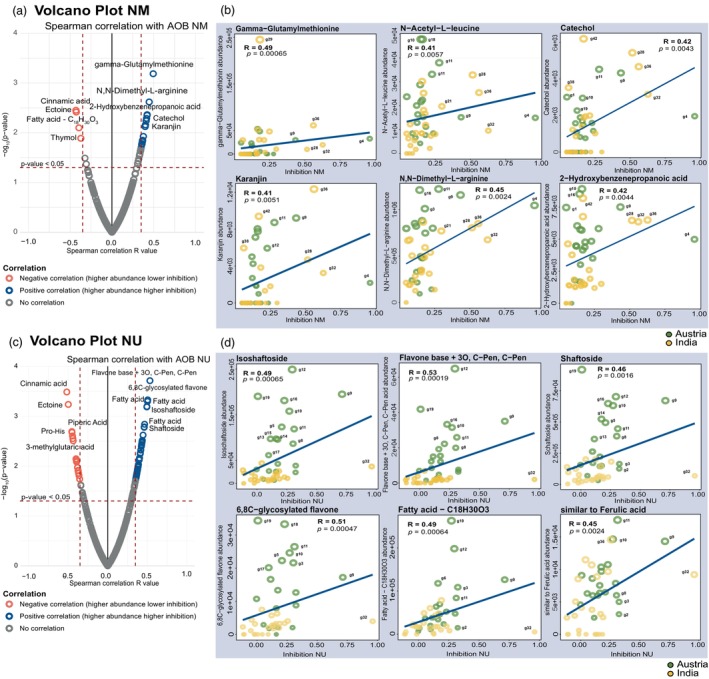
Inhibition of AOB strains by metabolites. Spearman correlation analysis was used to distribute 44 diverse wheat genotypes, considering secondary metabolite abundance in root exudate (identified using LC–MS) and the inhibition of AOB strains. Pairwise correlations were calculated between the 357 annotated metabolite levels and AOB inhibition. (a) Volcano plot of NM inhibition. A volcano plot was generated using 357 annotated metabolites identified in the root exudates and NM inhibition data. Blue circles represent metabolites with a Spearman's correlation *R*‐value higher than 0.40, which is statistically significant (*P* ≤ 0.05), indicating a positive correlation with NM inhibition. Red circles represent metabolites with a Spearman's correlation *R*‐value lower than −0.45, which is statistically significant (*P* ≤ 0.05), indicating a negative correlation with NM inhibition. Grey circles represent metabolites with a Spearman's correlation *R*‐value ≤0.40 to ≥ −0.40 and lacking statistical significance (*P* > 0.05). (b) NM inhibition distribution plot. Significantly correlated metabolites and NM inhibition activities were detected by Spearman's correlation analysis (*P* < 0.05). Dots were colour‐coded according to the origin of the genotypes. The size of each circle denotes the relative amount of metabolites and corresponding wheat genotypes. Only some of the highly significant positively correlated metabolites are shown. (c) Volcano plot of NU inhibition. A volcano plot was generated using 357 annotated metabolite levels identified in the root exudates and NU inhibition data. Blue circles represent metabolites with a Spearman's correlation *R*‐value higher than 0.40, which is statistically significant (*P* ≤ 0.05), indicating a positive correlation with NU inhibition. Red circles represent metabolites with a Spearman's correlation *R*‐value lower than −0.40, which is statistically significant (*P* ≤ 0.05), indicating a negative correlation with NU inhibition. Grey circles represent metabolites with Spearman's correlation *R*‐value ≤0.40 to ≥ −0.40 and lacking statistical significance (*P* > 0.05). (d) NU inhibition distribution plots. Significantly correlated metabolites and NU inhibition activities were detected using the Spearman's correlation approach (*P* < 0.05). Dots were colour‐coded according to the origin of the genotypes. The size of each circle denotes the relative amount of metabolites and corresponding to the wheat genotypes. Only some of the highly significant positively correlated metabolites are shown. NM (*Nitrosospira multiformis*). NU (*Nitrosomonas ureae*).

We further co‐analysed the variable nitrification inhibition data and the metabolomic data of the root exudates to identify metabolites responsible for the differential inhibition effect. To this end, Spearman correlation analysis was performed to derive a linear relationship between the inhibition capacity and the metabolites exuded by the roots of each genotype (Figures 5, 6; Figure S3). We identified metabolites that are positively correlated (*R* > 0.4, *P* ≤0.05) and showed higher inhibition activity (Figures 5b,d, 6b,d), for example, 2‐hydroxybenzenepropanoic acid (*R* = 0.54, *P* = 0.00015), isoschaftoside (*R* = 0.52, *P* = 0.00026), schaftsoside (*R* = 0.49, *P* = 0.00093), uric acid (*R* = 0.49, *P* = 0.00067), homoserine (*R* = 0.49, *P*‐value = 0.0008), L‐tryptophan (*R* = 0.48, *P* = 0.00099), L‐histidine (*R* = 0.43, *P* = 0.0035), karanjin (*R* = 0.41, *P* = 0.0051) and catechol (*R* = 0.42, *P* = 0.0043). Notably, some metabolites inhibited both AOB and AOA strains, such as 6, 8 C‐glycosylated flavones (NV (*R* = 0.54) and NU (*R* = 0.51)), isoschaftoside (NV (*R* = 0.52) and NU (*R* = 0.49)), schaftsoside (NV (*R* = 0.49) and NU (*R* = 0.46)) and phenylpropanoids, 2‐hydroxybenzenepropanoic acid (NV (*R* = 0.54) and NM (*R* = 0.42)). Other metabolites, such as alkaloids, amino acids and fatty acids, appeared to specifically inhibit AOA, like the ABOA alkaloid (NV (*R* = 0.31)). However, some metabolites, such as disaccharides (*R* = −0.35, *P* = 0.021) or metabolites related to nitrogen metabolism and hydroxylamine (*R* = −0.47, *P* = 0.0012), showed mild stimulatory effects on AOM (Table 1; Figure S3). Tables S1d and S2e,f show all Spearman Correlation *R*‐values and their corresponding *P*‐values.

**Table 1:** List of significant metabolites highly correlated to biological nitrification inhibition

Name of compounds	INCHIKEY	Level of annotation	Formula	Chemical class	Ion species	m/z	RT (min)	Mass accuracy (ppm)
2‐Hydroxybenzenepropanoic acid	CJBDUOMQLFKVQC‐UHFFFAOYSA‐N	Level 2	C_9_H_10_O_3_	Phenylpropanoids	[M‐H_2_O + H]^+^	149.0598	28.25	0.33
2‐Acetylpyrazine	DBZAKQWXICEWNW‐UHFFFAOYSA‐N	Level 2	C_6_H_6_N_2_O	Alkaloids	[M + H]^+^	123.0551	4.31	0.98
Isoshaftoside	OVMFOVNOXASTPA‐VYUBKLCTSA‐N	Level 2	C_26_H_28_O_14_	Flavonoids	[M + H]^+^	565.1555	30.76	0.6
Uric acid	LEHOTFFKMJEONL‐UHFFFAOYSA‐N	Level 1	C_5_H_4_N_4_O_3_	Nitrogenous bases	[M + H]^+^	169.0356	4.62	−2.4
Isatin	JXDYKVIHCLTXOP‐UHFFFAOYSA‐N	Level 1	C_8_H_5_NO_2_	Indol	[M + H]^+^	148.0393	30.73	−2.03
N,N‐Dimethyl‐L‐arginine	YDGMGEXADBMOMJ‐LURJTMIESA‐N	Level 2	C_8_H_18_N_4_O_2_	Amino acids and derivatives	[M + H]^+^	203.1496	2.53	−3.06
Flavone base +3O, C‐Pen, C‐Pen	LDVNKZYMYPZDAI‐UHFFFAOYSA‐N	Level 3	C_25_H_26_O_13_	Flavonoids	[M + H]^+^	535.1447	34.69	−1.13
6,8 C‐glycosylated flavone	–	Level 3	C_26_H_28_O_14_	Flavonoids	[M + H]^+^	565.1554	34.37	0.39
Shaftoside	MMDUKUSNQNWVET‐UHFFFAOYSA‐N	Level 2	C_26_H_28_O_14_	Flavonoids	[M + H]^+^	565.1552	32.19	0.12
L‐Tryptophan	QIVBCDIJIAJPQS‐VIFPVBQESA‐N	Level 1	C_11_H_12_N_2_O_2_	Amino acids and derivatives	[M + H]^+^	205.0970	19.36	−4.88
Oleamide	FATBGEAMYMYZAF‐KTKRTIGZSA‐N	Level 2	C_18_H_35_NO	Fatty acids and conjugates	[M + H]^+^	282.2790	68.79	−0.36
Oleamide	FATBGEAMYMYZAF‐KTKRTIGZSA‐N	Level 2	C_18_H_35_NO	Fatty acids and conjugates	[M + H]^+^	282.2790	69.18	−0.36
Sinensetin	LKMNXYDUQXAUCZ‐UHFFFAOYSA‐N	Level 2	C_20_H_20_O_7_	Flavonoids	[M + H]^+^	373.1280	31.77	−0.41
Cyclo(proline‐leucine)	SZJNCZMRZAUNQT‐UHFFFAOYNA‐N	Level 2	C_11_H_18_N_2_O_2_	Amino acids and derivatives	[M + H]^+^	211.1441	28.92	−0.21
Cyclo(proline‐leucine)	SZJNCZMRZAUNQT‐UHFFFAOYNA‐N	Level 2	C_11_H_18_N_2_O_2_	Amino acids and derivatives	[M + H]^+^	211.1440	27.99	−0.26
Lumichrome	ZJTJUVIJVLLGSP‐UHFFFAOYSA‐N	Level 1	C_12_H_10_N_4_O_2_	Flavins (Vitamins)	[M + H]^+^	243.0875	37.56	−0.81
Carnitine	PHIQHXFUZVPYII‐ZCFIWIBFSA‐N	Level 1	C_7_H_15_NO_3_	Amino acids and derivatives	[M + H]^+^	162.1120	2.41	−1.82
Acetyl‐DL‐carnitine	RDHQFKQIGNGIED‐UHFFFAOYSA‐N	Level 2	C_9_H_17_NO_4_	Amino acids and derivatives	[M + H]^+^	204.1228	3.39	−0.99
Zerumboneoxide	UXYYOHOTPOQJPD‐QNKGIFIZSA‐N	Level 2	C_15_H_22_O_2_	Terpenoids	[M + H]^+^	235.1692	40.82	−3.35
Perillic acid	CDSMSBUVCWHORP‐UHFFFAOYSA‐N	Level 2	C_10_H_14_O_2_	Terpenoids	[M + H]^+^	167.1066	30.19	−0.34
Hydroferulic acid	BOLQJTPHPSDZHR‐UHFFFAOYSA‐N	Level 2	C_10_H_10_O_3_	Phenylpropanoids	[M‐H_2_O + H]^+^	179.0703	29.25	0.05
ABOA	FZAQRVWPQCXSPC‐UHFFFAOYSA‐N	Level 2	C_9_H_7_NO_3_	Alkaloids	[M + H]^+^	178.0499	27.08	−3.58
Karanjin	LKPQNZRGGNOPPU‐UHFFFAOYSA‐N	Level 2	C_18_H_12_O_4_	Flavonoids	[M + H]^+^	293.0813	38.28	4.41
Sinapinic acid	PCMORTLOPMLEFB‐ONEGZZNKSA‐N	Level 2	C_11_H_12_O_5_	Phenylpropanoids	[M + H]^+^	225.0756	36.99	−0.49
Caffeic acid	QAIPRVGONGVQAS‐DUXPYHPUSA‐N	Level 2	C_9_H_8_O_4_	Phenylpropanoids	[M + H]^+^	181.0495	25.82	−0.05
Syringic acid	JMSVCTWVEWCHDZ‐UHFFFAOYSA‐N	Level 1	C_9_H_10_O_5_	Phenylpropanoids	[M + H]^+^	199.0601	26.44	0.41
Catechol	YCIMNLLNPGFGHC‐UHFFFAOYSA‐N	Level 2	C_6_H_6_O_2_	Phenylpropanoids	[M + H]^+^	111.0438	28.49	−0.99
Ala‐Ala	DEFJQIDDEAULHB‐QWWZWVQMSA‐N	Level 2	C_6_H_12_N_2_O_3_	Amino acids and derivatives	[M + H]^+^	161.0916	2.38	−2.85
Homoserine	UKAUYVFTDYCKQA‐VKHMYHEASA‐N	Level 2	C_4_H_9_NO_3_	Amino acids and derivatives	[M + H]^+^	120.0649	2.34	−4.16
Violantin	FIAAVMJLAGNUKW‐UHFFFAOYSA‐N	Level 2	C_27_H_30_O_15_	Flavonoids	[M + H]^+^	595.1671	28.49	−3.54
Flavonoid (C17H14O7)	–	Level 3	C_17_H_14_O_7_	Flavonoids	[M + H]^+^	331.0811	36.91	3.57
L‐Histidine	HNDVDQJCIGZPNO‐YFKPBYRVSA‐N	Level 1	C_6_H_9_N_3_O_2_	Amino acids and derivatives	[M + H]^+^	156.0763	2.28	−3.12

**Figure 7:**
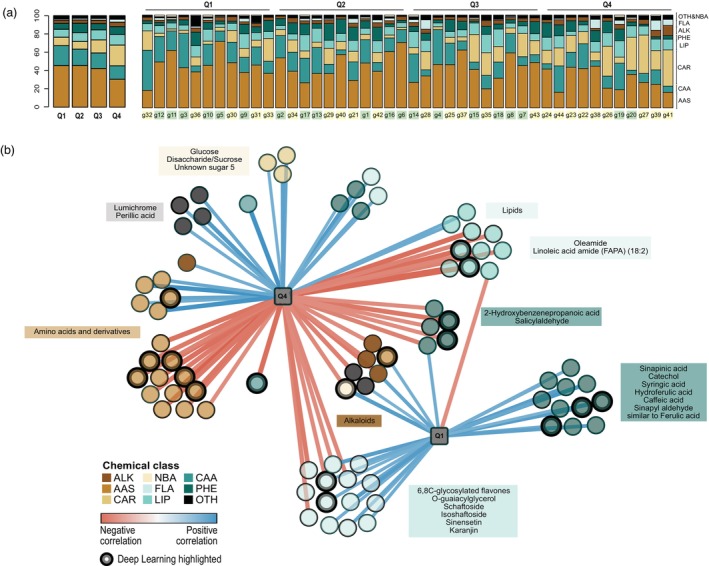
(a) Changes in the relative abundance of different metabolite chemical classes by percentage. Averaged quartile group and independent genotypes were represented. (b) PLS discriminant analysis network to determine the positive and negative correlation of the metabolites with the inhibitory activity of AOA and AOB strains. The genotypes were classified in quartiles by their inhibition capacity (from Q1; highest inhibitory activity by the genotypes to Q4; lowest inhibitory activity by the genotypes). The correlation cut‐off was 0.35, and the edge colour reflected positive (blue) or negative (red) interactions. Metabolites identified using the Deep Learning approach are indicated with thick lines in nodes. ALK, alkaloids; AAS, amino acids and derivatives; CAR, carbohydrates, sugars; NBA, nitrogenous bases; FLA, flavonoids; LIP, lipids, fatty acids; CAA, carboxylic acids; PHE, phenylpropanoids; OTH, others that include terpenoids, vitamins, quinones, etc.

Furthermore, PLS regression analysis was performed on the inhibition values of different AOB and AOA microorganisms to evaluate the differential exudation pattern among the various genotypes (Figures S4 and S5). The PLS networks determine the regulation of the metabolites and their impact on the inhibition capacities of these genotypes (Figure 7). Highly accumulated metabolites in the root exudates demonstrated greater inhibition capacity and had positive regression, whereas metabolites that accumulated more in exudates but showed lower inhibition capacity had negative regression. Metabolites grouped in the class of flavonoids, phenylpropanoids and amino acids strongly inhibit AOA (NF and NV). Among these, the previously mentioned flavonoids, such as isoschaftoside and schaftsoside, and phenylpropanoids like 2‐Hydroxybenzenepropanoic acid, syringic acid and sinapinic acid stood out. In addition, some of these metabolites seemed to play a significant role in inhibiting AOB (NM and NU), such as 2‐Hydroxybenzenepropanoic acid, N, N‐dimethyl‐L‐arginine, 2‐acetylpyrazine and N‐Acetyl‐DL‐glutamic acid. Root exudates containing metabolites with low inhibition activity include terpenoids (perillic acid) and vitamins (lumichrome) (Table 1).

### Deep learning analysis reveals combinatorial activity of root exudate metabolites on BNI

In the deep learning (DL) analysis, a Deep Canonical Correlation Analysis (DCCA) model was trained to establish the relationship between metabolites and BNI activity. Metabolites were subsequently imported into a Genetic Algorithm (GA), with the DCCA model serving as the fitness function to evaluate the correlation between each metabolite and BNI activity (for detailed information, see Appendix S1). From 200 repetitions of the GA, metabolites occurring with a higher frequency (more than 10) showed a stronger correlation with BNI activity, while a lower frequency of metabolites indicated a weaker correlation. The algorithm thus selected metabolites with a strong correlation to BNI activity (Table S4g). Using the deep learning method, we identified several metabolites significantly associated with BNI activity in different ammonia‐oxidizing strains (AOA and AOB). In AOA‐NF, the key metabolites included 2‐Acetylpyrazine (frequency (f) = 30), 2‐Hydroxybenzenepropanoic acid (*f* = 19), N‐Acetyl‐DL‐glutamic acid (*f* = 19), Ala‐ala (*f* = 17), 6,8 C‐Glycosylated flavone (*f* = 8), Uric Acid (*f* = 10) and Isoschaftoside (*f* = 8). For AOA‐NV, significant metabolites were Pyroglutamate (*f* = 10), L‐Phenylalanine (*f* = 9), L‐Glutamic acid (*f* = 9) and 2‐Phenylacetamide (*f* = 6). In AOB‐NM, metabolites such as N,N‐Dimethyl‐L‐arginine (*f* = 24), catechol (*f* = 16), 2‐Acetylpyrazine (*f* = 16) and 2‐Hydroxybenzenepropanoic acid (*f* = 6) showed strong correlations. In AOB‐NU, significant metabolites were N‐Acetyl‐DL‐glutamic acid (*f* = 20), 2‐Acetylpyrazine (*f* = 15), Oleamide (*f* = 8) and 6,8 C‐glycosylated flavone (*f* = 5). These classes of metabolites showing strong inhibition capacity, including amino acids, flavonoids, phenylpropanoids and fatty acids, are mapped on Figure 7 (Table S4g). We observed a good agreement between the DL and multivariate statistics results. The significant metabolites based on different statistical and deep learning methods are represented in Appendix S2 together with chemical structure, chromatographic peaks and mass spectra. The results are confirmed by an 80/20% cross‐validation split with five repeats and showed a consistent ranking for the top few metabolites. This confirms that the GA‐DCCA model is robust in identifying key BNI‐related metabolites, as the frequency of these metabolites in the GA population remained stable with little variation in data partitioning.

## Discussion

Screening wheat cultivars for BNI capacity emerges as a crucial demand for sustainable agricultural processes. Modern wheat cultivars often do not carry detectable BNI capacity (Subbarao *et al*., 2007a) However, these early reports came from tests of a small number of genotypes of each target plant species and, in most cases, used inhibition testing systems that fail to assess the full inhibitory potential of root exudates against active AOM in soil. Hence, screening of the BNI activity of a broader range of modern wheat varieties using ecologically relevant screening systems has the potential to facilitate breeding for elite wheat cultivars with high BNI activity. Moreover, the PANOMICS approach integrated with transformative biological technologies designed for genetic mitigation (Ghatak *et al*., 2023; Hu *et al*., 2025; Mishra *et al*., 2024; Raza *et al*., 2025; Weckwerth *et al*., 2020) can enhance the understanding of the dynamic and interconnected biological systems (i.e. root–soil–microbiome ecosystem), aiding BNI‐enabled cropping systems that can rein in soil‐nitrifier activity, to help reduce greenhouse gas (GHG) emissions (Lehmann *et al*., 2020) and globally make farming nitrogen efficient and sustainable. In the present study, we determined the root exudate metabolome of 44 diverse wheat genotypes from different geographical locations (India and Austria) for nitrification inhibition capacity using a novel fast‐track BNI assay and identified a group of metabolites that are associated with the nitrification inhibition.

Water is the most effective solution for accumulating root exudates (Ghatak *et al*., 2022; Valentinuzzi *et al*., 2015). However, it has certain limitations when compared to naturally released compounds in soil. These limitations include the absence of the rhizosphere microbiome, potential overestimation of in situ stability and concentration, and the loss of chemical gradients due to the disruption of spatial heterogeneity and soil matrix effects (Canarini *et al*., 2019). Additionally, some hydrophobic BNI compounds are poorly soluble in water, which may result in their loss during extraction (Subbarao *et al*., 2015). The main objective of our study was to identify metabolites released under natural conditions; therefore, we used agricultural soil instead of a hydroponic system, as the latter has less ecological relevance. In this context, the present study employed a soil‐based sampling approach, which allows for the collection of root exudates targeting the entire root system (Oburger and Jones, 2018). Using this approach, we have resolved an unexpected complexity of the root exudate metabolome by detecting more than 6000 metabolites and metabolic features in a combined GC–MS and LC–MS analysis. This approach is crucial for elucidating ‘the missing link’ driving plant–microbe–soil interactions, the formation of rhizosphere patterns and the relation of root exudates and nitrification processes in the soil.

### Ammonia oxidation inhibition is associated with natural variation of the wheat root metabolome

BNI activity has been mainly attributed to the release of biologically active compounds from roots that suppress or regulate the growth and function of the nitrifiers. This eventually leads to the deceleration of the nitrification process in soil (Nardi *et al*., 2020; Subbarao and Searchinger, 2021). This study provides the string of evidence that genotypic differences in the metabolite composition of root exudates explains the variation in the inhibitory activity on the AOB and AOA strains tested.

Several studies have indicated that the effects of BNIs vary depending on the microbial lineage or even the microbial strain tested; some BNIs show largely different activity on AOA and AOB (Issifu *et al*., 2024; Kolovou *et al*., 2023a). Therefore, as a first step in our quest for assessing the BNI potential from the root exudate metabolome of wheat genotypes, we developed a high‐throughput, fast‐track screening assay for AOA and AOB characterized by (i) a short turn‐around time, ranging from 5 h (AOA) to 22 h (*N. ureae*), aligned with other similar screening platforms (Beeckman *et al*., 2023; O'Sullivan *et al*., 2016) and (ii) high ecological relevance compared to earlier screening tools (Beeckman *et al*., 2023; O'Sullivan *et al*., 2016; Subbarao *et al*., 2006; Sun *et al*., 2016). The developed system involved testing on three phylogenetically diverse and soil‐relevant AOB strains (Sedlacek *et al*., 2019) compared to a single *N. europaea* strain (Subbarao *et al*., 2006; Sun *et al*., 2016), as well as two terrestrial AOA with distinct physiology (Han *et al*., 2024) and phylogeny (Alves *et al*., 2018), representing key nitrifiers in the rhizosphere of important crops (Lee *et al*., 2024). The responses of the individual AOM strains in our screening platform to known BNIs and SNIs were in accord with their range of activity in previous studies, adding further validity to our newly developed approach (Kaur‐Bhambra *et al*., 2022; Kolovou *et al*., 2023a; Papadopoulou *et al*., 2020).

Following the validation of the screening process, root exudates of the 44 wheat genotypes were tested on two AOB and two AOA representing nitrifiers from both the bacterial and archaeal domains. Representation from both domains is crucial as each domain has unique cellular structures and differing, albeit unresolved, ammonia oxidation pathways (Lancaster *et al*., 2018). The screening of root exudates against the nitrifiers showed a clear differentiation between the different wheat genotypes (Figure 4) as established for the metabolomes (Figures 1 and 2). The two information datasets (inhibition and metabolome) were further integrated to identify BNI active compounds.

### Genotype‐specific metabolome determines varying nitrification inhibition capacity

To identify metabolites that inhibit both AOB and AOA, a PLS‐DA analysis was performed considering varying inhibition capacity of the root exudates (Figure 7; Table S4f). This analysis classified wheat genotypes into quartiles based on their inhibition capacity across all the evaluated microorganisms (AOB and AOA) with Q1 representing the highest inhibition and Q4 the lowest (Figure 7a). This clustering allowed us to observe trends in the metabolic profiles of the root exudates (Figure 7a). The genotypes that exhibited the strongest inhibition (Q1) demonstrated a higher concentration of amino acids, amines, amino acid derivatives, phenylpropanoids and flavonoids and a lower concentration of carbohydrates.

A subsequent network analysis allowed for the identification of combinatorial effects of these compounds and specific phenylpropanoids and flavonoids that were found to be present at the highest concentration in root exudates with the greatest inhibitory capacity (Figure 7b). These included phenylpropanoids such as catechol, sinapinic acid, syringic acid, caffeic acid and hydroferulic acid as well as glycosylated flavones, such as 6,8 C‐glycosylated flavone, isoshaftoside and shaftoside (Figure 7b). According to Strehmel and co‐workers, flavonoids accumulate in plants and represent a significant proportion of root exudates (Strehmel *et al*., 2014). Both aglycones and glycosides of flavonoids have been identified in these exudates (Wang *et al*., 2024). The glycosylation of flavonoids enhances their solubility, facilitating their secretion into the rhizosphere. Once in the rhizosphere, microbes or plant exoenzymes break them down into aglycones (Wang *et al*., 2024). C‐glycosides like isoshaftoside and shaftoside serve a variety of functions, including acting as siderophores, antibiotics, antioxidants, attractants and feeding deterrents (Brazier‐Hicks *et al*., 2009). Importantly, these flavonoids can influence nutrient availability by altering soil chemistry, functioning as metal chelators (Cesco *et al*., 2010). Similar to SNI such as allylthiourea, flavonoids can form complexes with copper, which is essential for the activity of the membrane metalloenzyme ammonia monooxygenase (AMO) (Caranto and Lancaster, 2017).

Several of the identified compounds have previously been associated with BNI activity, suggesting that the co‐analysis of inhibition and metabolome was successful. A prominent example of this is syringic acid (SA) (Figure 7b; Table S4e,g). Lu and co‐workers identified SA as a BNI in rice root exudates, showing equivalent inhibition activity on both AOB and AOA in different types of soils (Lu *et al*., 2022). Similarly, previous studies with caffeic acid showed strong growth‐inhibition activity on AOA (*N. viennensis*, *Ca.* N. franklandianus, and *Nitrosotalea sinensis*) (Kolovou *et al*., 2023b) and AOB strains (*Nitrosospira briensis*, *N. europaea*, *N. multiformis* and *Nitrosospira tenuis*) (Issifu *et al*., 2024). Although the inhibitory mode of action of these compounds remains unknown, it is hypothesized that, like in other microorganisms, they may participate in the disruption of the cytoplasmic membrane (Andrade *et al*., 2015).

Within the flavonoids category, 6,8 C‐glycosylated flavones, two apigenin 6,8 C‐di‐glycoside isomers (isoschaftoside and schaftoside) and the furanoflavonoid Karanjin were positively correlated with high inhibitory activity on AOM. Karanjin has been reported before as a highly efficient nitrification inhibitor, much more potent than DCD, that effectively reduced N_2_O emissions by approximately 92–96% (Majumdar, 2002). Schaftoside and isoschaftoside are bioactive natural products that are widely present in cereal crops such as rice, maize, wheat and sorghum, as well as medicinal herbs (Wang *et al*., 2020). Their biosynthesis might be part of the defence system of plants against abiotic and biotic stressors; for example, their concentration in rice shoots increased by approximately 40% after infection by the fungus *Magnaporthe oryzae* causing rice blast (Wang *et al*., 2020). From these combined results, it can be concluded that different plant genotypes rely on a diverse group of metabolites that can inhibit both AOB and AOA simultaneously, thereby reducing soil nitrification (Figures 4 and 7). Deep learning analysis further confirmed the strong nitrification inhibition capacity of the same class of metabolites (Figure 7b).

In some cases, root exudates were observed to have a positive effect on nitrifying organisms (Figure 4e). Metabolites that appear to show some stimulatory effects in root exudates of Q4 genotypes include carbohydrates such as glucose and sucrose, along with vitamins (lumichrome), terpenoids (zerumboneoxide and perillic acid), amines (carnitine and acetyl‐DL‐carnitine) and hydroxylamine, an intermediate in ammonia oxidation by AOM (Vajrala *et al*., 2013). Carbohydrates, particularly glucose, can act as general chemo‐attractants (Badri *et al*., 2013) and promote heterotrophic nitrification (Lan *et al*., 2023). Accordingly, our study demonstrates the importance of screening a large number of genotypes in germplasm collections to distinguish growth inhibiting and stimulating effects as well. The exact mechanisms of these effects, however, demand further studies.

### Different rooting structure of spring wheat and winter wheat influences nitrification inhibition capacity

In this study, the root exudates of Indian wheat genotypes showed a lower nitrification inhibition capacity compared to Austrian genotypes, with a few exceptions such as g32 and g36. These specific Indian genotypes are characterized by higher carbohydrate content and fewer carboxylic acids. On the other hand, the genotypes demonstrating the highest inhibition capacity generally contain elevated levels of phenylpropanoids, flavonoids and amino acids. It is important to note that Indian wheat genotypes are classified as spring wheat, while the Austrian genotypes are classified as winter wheat. Notably, winter wheat has approximately twice the rooting depth of spring wheat (Thorup‐Kristensen *et al*., 2009). The early sowing of winter wheat is recognized as an effective strategy to mitigate nitrate leaching. This is because deeper root penetration and increased biomass production enhance nitrogen uptake during the autumn period (Rasmussen and Thorup‐Kristensen, 2016). The deeper rooting characteristics of winter wheat facilitate nitrogen absorption at depths greater than 1 m (Thorup‐Kristensen *et al*., 2009), which ultimately results in reduced levels of nitrogen leaching.

These findings align with our previous study on pearl millet, which suggested that high levels of root exudation are associated with increased root length, potentially affecting BNI activity (Ghatak *et al*., 2022). This phenomenon may be attributed not only to enhanced root growth and increased nitrogen assimilation but also to the specific composition of compounds exuded by the roots. The higher prevalence of certain molecules with potential BNI activity in Austrian wheat genotypes may support this hypothesis. However, further research is needed to fully understand the impact of root architecture on BNI and nitrifiers.

## Conclusion

This study examined the complex role of root exudate metabolomes from various wheat genotypes in inhibiting nitrification in the rhizosphere. We utilized a combination of advanced metabolomic analysis and a novel, high‐throughput nitrification inhibition assay, representing a significant advancement over previous testing methods. This co‐analysis allowed us to discover genotype‐specific differences in the metabolomic profiles of root exudates, which correlated well with their capacity to inhibit nitrification. These variations are linked to the simultaneous accumulation of several compounds, including primary metabolites such as oxalic acid, oleamide and amino acids, as well as secondary metabolites like isoshaftoside, shaftoside and 2‐hydroxybenzenepropanoic acid. We hypothesize that the concurrent presence of certain metabolites, rather than a single compound, may drive nitrification inhibition in the rhizosphere. Our findings underscore the importance of re‐evaluating the collective role of the root exudate metabolome and especially its natural variation, which encompasses a wide range of phytochemicals, in achieving nitrification inhibition. These identified metabolites are essential for the determination of the relationship between NUE and nitrification inhibitory effects because N uptake varies strongly between the different varieties and between the different growth stages of the plant (Sun *et al*., 2016). Therefore, future studies should be focused on both the collective and individual BNI potential of these identified metabolites and generate real‐time data using ^15^N‐labelling methods to establish the relationship between NUE and BNIs. This will offer deeper insights into the mechanisms that underpin biological nitrification inhibitors (BNIs) in wheat. The presence of BNIs in different wheat genotypes opens up possibilities for breeding this trait into modern elite lines, particularly those with increased root length, which can significantly enhance below‐ground nitrogen acquisition and reduce nutrient leaching. This will provide a nature‐based solution for sustainable agroecosystems (Weckwerth *et al*., 2025).

## Materials and methods

### Plant growth and root exudate collection

In the present study, 44 diverse wheat genotypes from Austria and India were evaluated for root exudate composition. The plants were grown in a natural field soil obtained from the Lower Austrian region (48°19′55.1″ N and 16°10′08.2″ E) under controlled greenhouse conditions (12 h of light, an average of 220 μmol/m^2^/s, max 30 °C during daytime, 26 °C at night (±2 °C) and 60% relative air humidity). As an initial fertilizer, 0.1% NPK was added for the proper growth of the plants and no pesticides were used.

Plants were harvested at the developmental stage of 50–59 on the wheat BBCH scale. Roots were carefully removed from the soil, thoroughly cleaned with deionized water and incubated in 100 mL distilled water. The root exudates were allowed to accumulate for 48 h at a lower temperature primarily to extract exudates from the root apoplast. After incubation, the root exudate solution was filtered (0.2 μm pore size filter) to remove any root debris, and additionally, the samples were centrifuged at 20 000 ***g*** for 10 min at 4 °C (Ghatak *et al*., 2022). Each 100 mL sample was concentrated to a volume of 15 mL using a rotary evaporator under vacuum at 40 °C. The concentrated root exudates were stored at 4 °C until further analysis. The dry weight of the roots was also recorded.

### High‐throughput nitrification inhibition screening

#### AOM strains and growth conditions

*Nitrosospira multiformis* ATCC25196 (NM) and *Nitrosomonas ureae* Nm10 (NU) (obtained from University of Lyon, France), were cultivated in Skinner and Walker (SW) medium (Skinner and Walker, 1961). *Nitrosomonas communis* Nm2 (NC) (obtained from University of Alberta, Canada) was grown in NCOM medium (Koops *et al*., 1991) adapted to a 3 mM NH_4_^+^ substrate. Axenic liquid cultures of the terrestrial AOA *N. viennensis* EN76 (NV) and ‘*Candidatus* Nitrosocosmicus franklandianus C13’ (NF) (obtained from University of Lyon, France) were grown in freshwater medium (FWM) buffered with HEPES to pH of 7.5 and supplied with 2 mM NH_4_^+^ following (Reyes *et al*., 2020) with slight modifications (for more information, see Appendix S1).

#### Development and validation of the screening system

The workflow of the AOB and AOA fast‐track system is presented in Figure 3. Regarding AOB, cultures at the late logarithmic phase were filtered through hydrophilic 0.22 μm polyethersulphone (PES) membrane filters (Frisenette ApS, Knebel, Denmark), harvested and further concentrated through centrifugation at 11 000 ***g*** for 10 min, into a final volume of 2 mL. This concentrated inoculum was then diluted with sterile AOB medium at ratios of 1:10 for *N. multiformis* and *N. communis* and 0.7:10 for *N. ureae*, and the concentrated culture was dispersed to a 96‐well plate (BRAND GMBH & CO KG, Wertheim, Germany). The plate was incubated with a lid, statically, at 28 °C for 10 h (*N. communis*) or 25 h (*N. multiformis* and *N. ureae*). Nitrite production was determined colorimetrically at 540 nm (Shinn, 1941). Samples for measuring the abundance of the *amoA* gene were collected at specific protocol steps as outlined in Figure 3.

For AOA, *N. viennensis* cultures at the late logarithmic phase were concentrated via centrifugation at 21 000 ***g*** for 30 min at 4 °C, while *Ca.* N. franklandianus cultures of the same growth stage, were concentrated on Millipore® sterile hydrophilic 0.22 μm mixed cellulose esters (MCE) membrane filters using a sterile filtration unit. Both cell types were harvested and stored at 4 °C until further processing. AOA culture concentrates were allowed to prewarm at 42 °C before being dispersed in a 96‐well plate, which was incubated with a lid, statically, at 42 °C, for 3–5 h and NO_2_ production measured. Samples for determination of cell density were taken at specific protocol steps as outlined in Figure 3.

The validity of our screening platform was tested with a range of known SNIs such as DMPP, nitrapyrin, 1,2‐dihydro‐6‐ethoxy‐2,2,4‐trimethylquinoline (ethoxyquin) and BNIs such as methyl 3‐(4‐hydroxyphenyl) propionate (MHPP), sakuranetin and 1,9‐decanediol (Appendix S1—Table S1). The selected NIs were utilized at a range of concentrations, expected to impose from slight to full inhibition of the activity of AOM strains (Appendix S1—Table S2), as depicted from earlier studies (Kolovou *et al*., 2023b; Papadopoulou *et al*., 2020). For each AOM strain, 200 μL of the concentrated culture were added to each well, followed by the addition of each inhibitor. Triplicate cultures for each inhibitor x concentration combination, along with respective control cultures of 0.1% v/v ddH_2_O and 0.1% v/v DMSO (AOB) or 0.02% v/v DMSO (AOA) were established, following the observed DMSO tolerance limits for the concentrated AOM cultures. Nitrite determination was used at a single (AOA) or multiple selected time points (AOB) to determine the Ammonia Oxidation Inhibition percentage (AOI%) and the EC_50_ value. A detailed description of the development and validation procedure of the screening system is provided in the Appendix S1—Figures S1–S23.

#### Wheat root exudate screening

Dried root exudates were resuspended in sterile ddH_2_O and further diluted to standardize their concentration based on the original dry root weight. Negative control treatments with respective ddH_2_O volumes were also included. The 96‐well plates were incubated as described above. Nitrite determination was used to follow the inhibition of the AOM cells and determine the AOI% at one selected time point, approximately 5 h for *N. viennensis* (NV) and *Ca.* N. franklandianus (NF), 20 h for *N. multiformis* (NM), and 22 h for *N. ureae* (NU). Due to limited availability of root exudates, *N. communis*, the least sensitive AOB reporter strain, as derived from testing with pure BNIs and SNIs, was excluded from the screening process.

### Metabolome profiling of root exudates

#### Gas chromatography coupled to mass spectrometry (GC–MS)

Metabolite extraction was performed according to Weckwerth *et al*. (2004), with slight modifications. All analysis steps, including sample derivatization and GC‐TOF‐MS (gas chromatography coupled to time‐of‐flight mass spectrometry), were carried out as previously described (Ghatak *et al*., 2018; Weckwerth *et al*., 2004; Zhang *et al*., 2024).

Data analysis was performed using the ChromaTOF (Leco) software. Chromatograms of different samples were used to generate a reference peak list, and all other data files were processed against this reference list. Retention index markers were used to calculate retention indices of compounds and chromatographic alignment. Deconvoluted mass spectra were matched against an in‐house mass spectral library, and the retention index was used for peak annotation. Peak annotations, as well as peak integrations, were checked manually before exporting peak areas for relative quantification into Microsoft Excel (Table S1a). Areas of different trimethylsilyl derivatives of single metabolites were summed, and from methoxyamine products, only one peak was selected for further analysis (Ghatak *et al*., 2018; Weckwerth *et al*., 2004; Zhang *et al*., 2024). A detailed methodology of the GC–MS analysis is provided in the Appendix S1.

#### Liquid chromatography coupled to mass spectrometry (LC–MS)

Root exudate analysis was performed using an UltiMate 3000 UHPLC system coupled to an Orbitrap Elite mass spectrometer (Thermo Fisher Scientific) according to the method described by Schindler and co‐workers, with slight modifications (Schindler *et al*., 2021). For untargeted LC–MS analysis, Xcalibur (Thermo Scientific, Waltham, MA, USA) RAW files were converted to mzML files using MSconvert (ProteoWizard) (Chambers *et al*., 2012), followed by feature extraction using MZmine 4 (Heuckeroth *et al*., 2024; Pluskal *et al*., 2010). The MS1, MS2 and reference spectra of annotated metabolites are summarized in Table S2a and MSP files (data excluded). A detailed methodology of the LC–MS analysis, processing and feature annotation is provided in the Appendix S1. The level of identification was determined for each LC–MS m/z feature according to the Metabolomics Standard Initiative (Sumner *et al*., 2007) and is listed in Table S2a.

### Data analysis and statistical methods

#### Statistical analysis for BNI activity screening

EC_50_ values represent the concentrations that inhibit ammonia oxidation activity by 50% compared to the respective control treatment. Ammonia Oxidation Inhibition percentage (AOI%) for NIs and root exudates refers to the proportional inhibition of AOM activity compared to the control. For AOB dose–response curves, fitting a single sigmoidal pattern to normalized nitrite production data were used for EC_50_ determination, as described previously (Papadopoulou *et al*., 2020). For linear or polynomial AOB responses, the slopes of the activity linear regression curves at each dose level were utilized to determine the AOI% for each inhibitor concentration. AOI% was plotted against the respective concentrations to estimate the EC_50_ values. For AOA data, AOI% values for one time‐point were calculated as the proportional production of nitrite relative to the control, and EC_50_ values were determined as for the AOB.

Differences in the AOI% values of the root exudates were evaluated per AOM strain with Kruskal–Wallis tests and the Dunn's post hoc test with the Bonferroni *P*‐value adjustment method. The root exudate AOI% values heatmap was generated with hierarchical clustering and Euclidean distances for both column and row dendrograms. The effect of the AOM strain on the root exudate AOI% values was assessed through a Kruskal–Wallis test and the Dunn's post hoc test with the Bonferroni *P*‐value adjustment method, and the effect of the AOM domain and wheat genotype origin on the root exudate AOI% values was assessed via a Wilcoxon test. Details on data and statistical analyses and resources used are provided in Appendix S1.

#### Statistical analysis and deep learning approach

Data processing and statistical analyses were performed using the R software (R Core Team, 2024). The GC–MS metabolite abundances (Table S1a) were normalized by weight and log_10_ transformed. The LC–MS peak areas (Table S2a) were pre‐processed using abundance balancing (values were sample‐centric, and then each value was multiplied by the average intensity in the pRocessomics package available at https://github.com/Valledor/pRocessomics). Venn diagram analysis, heat maps and principal component analysis (PCA) were obtained from both metabolomic datasets (Tables S1b,c and S2b–d). Differentially excreted metabolites of different wheat origins were analysed using the Wilcoxon test, and a volcano plot analysis displayed the fold change (FC) differences and statistical significance for each metabolite (*P*‐value) (Tables S1d, S2e,f). Spearman correlations were used to identify the linear accumulated metabolites with the inhibitory activity of different wheat genotype root exudates against different microorganisms. ‘cor_mat’ and ‘cor_get_pval’ functions from rstatix package were employed to obtain *R* and *P*‐value of each metabolite (Tables S1d, S2e,f, S4e). Statistically significant correlation scatter plots were constructed using the ggplot2 and ggcorrplot R packages. For each microorganism (AOA‐NF, AOA‐NV, AOB‐NM and AOB‐NU), Partial Least Squares regression (PLS) is considered as the single dependent variable. The PLS algorithm was employed to determine correlations between the predictors (metabolite matrix) and response variables (root exudate inhibition in different microorganisms) (Table S4a–d). The PLS analysis used the R package mixOmics (Rohart *et al*., 2017). Generated networks were visualized and filtered (only edges equal or higher than 0.4 [NF], 0.35 [NV], 0.38 [NM] and 0.45 [NU] were maintained) in Cytoscape v.3.10.2. Partial least squares discriminant analysis (PLS‐DA) was used to elucidate the crucial metabolites associated with root exudate inhibition in the studied microorganisms (AOA and AOB) (Table S4f). The wheat genotypes were classified into quartiles based on their inhibition capacity: Q1, highest inhibitory root exudate genotype to Q4, and lowest inhibitory root exudate genotype (Table S3b). The generated network was visualized and filtered (only edges equal to or higher than 0.4 were maintained) using Cytoscape v.3.10.2 (Shannon *et al*., 2003). The PLS‐DA network incorporates Deep Learning (DL) metabolites identified through an advanced feature selection technique, a Deep Canonical Correlation Analysis (DCCA) model (Andrew *et al*., 2013), a genetic algorithm (GA) (Lambora *et al*., 2019) and comprehensive data analysis (Table S4g). The PLS‐DA model incorporates key metabolites identified through an integrated deep learning‐based feature selection approach, combining Deep Canonical Correlation Analysis (DCCA) (Andrew *et al*., 2013), a genetic algorithm (GA) (Lambora *et al*., 2019) and comprehensive data analysis (Table S4g). The GA uses a population where each individual corresponds to a random triplet of metabolites. In each iteration, the GA selects and evolves metabolite groups based on their predictive performance, which is evaluated using the DCCA model. The DCCA model quantifies the correlation between selected metabolites and BNI activity, guiding the GA search towards selecting for more relevant metabolites in the triplets. After 200 GA repetitions, the occurrence of metabolites in the highest performing triplets is counted to determine the frequency of each metabolite. This approach enables systematic identification of key BNI‐related metabolites and insights into their potential biological relevance. Further methodological details are provided in Appendix S1.

## Funding

This work was funded by the Grantham Foundation as part of the project ‘*Pipeline for Development and Commercialization of Biological Nitrification Inhibitors to Mitigate GHG Emissions from Cultivated Soils*.’ AG is also thankful to the Vienna Metabolomics Center (VIME). CHL is thankful to the HORIZON‐MSCA‐2023‐PF‐01‐01 (Project ID: 101153366). YM is supported by China Scholarship Council (CSC) (Grant number: 202309040003) and completion grant of the Vienna Doctoral School Ecology and Evolution of the Faculty of Life Sciences, University of Vienna, Austria. AEK acknowledges partial funding by the MSc programme ‘HOSMIC – Host‐Microbe Interactions’. MK, LH, DGK, CS and ESP acknowledge funding support from the European Union's Horizon 2021–2027 research and innovation programme ACTIONr, under grant agreement No. 101079299.

## Conflict of interest

The authors declare no competing financial interest.

## Author contributions

Conceived the study: WW, AG, PC, MK, DGK, CS and ESP; Performed and designed the experiment: AG, PC, WW, SZ, AEK, ΑΜ, HR, MD and LHH; Performed GC–MS measurements: PC and AG; Performed LC–MS measurements: FS and AG; Analysed GC–MS raw data: AG and SZ; Analysed LC–MS raw data: CLH and FS; Performed fast‐track nitrification assay: AEK, ΑΜ and HR; Analysed the data: PC, CLH, WW, AG, SZ, YM, SW and AEK; Drafted the manuscript: PC, AG, AEK, CLH, YM and WW; Critically reviewed the manuscript: RRM, SS, GB, MK, AM, LHH, DGK, CS, ESP and WW; Edited the manuscript: PC, MK, DGK, CS, ESP and WW. All the authors read and agreed to the final version of the manuscript.

## Supporting information


**Appendix S1** Details and validation of the Fast‐track screening system using SNIs and BNIs, metabolomics analysis (GC‐MS/LC‐MS) and deep learning algorithm.

**Appendix S2** Chemical structure, chromatographic peak intensity and mass spectra of the significant metabolites.

**Figure S1** GC–MS metabolomics analysis. Primary metabolites identified in the root exudates of different wheat genotypes originated from Austria and India. (a) Venn diagram. (b) Bar plots of each chemical class of the metabolites. The height of the bar represents the sum of the abundance of metabolites from each origin (Austria and India). (c) Principal Component Analysis. Principal Component 1 (PC1) ranked the metabolites in the root exudates. Loadings of PC1 are shown in rows. The bar colour indicates the origin of the genotypes (Austria and India), in which each metabolite is median.
**Figure S2** LC–MS metabolomics analysis. Secondary metabolites identified in the root exudates of different wheat genotypes originated from Austria and India. (a) Principal Component analysis. All detected features were used for PCA analysis. (b) Volcano plot. A volcano plot was generated with a 44‐genotype dataset (20 Austrian genotypes and 24 Indian genotypes, and 6632 metabolic features). Green circles represent metabolites with a fold change ≥2 that were statistically significant (*P* ≤ 0.01), indicating higher accumulation in Austrian genotypes than in Indian genotypes. Yellow circles represent metabolites with a fold change ≤ −2 that was statistically significant (*P* ≤ 0.01), indicating higher accumulation in Indian genotypes than in Austrian genotypes. Grey circles represent metabolites with a fold change ≤2 to ≥ −2 and lacking statistical significance (*P* > 0.01). (c) Upset plot. Three hundred and fifty‐seven annotated features (Annotation levels 1–4) were used for the upset diagram. (d, e) Principal Component Analysis of 357 annotated features (Annotation level 1–4). PC1 and PC2 are top‐ranked metabolites in root exudates. The highest loadings (15 highest and 15 lowest) of PC1 and PC2 are shown in rows for each principal component. Bar colours indicate the origin of the genotypes (Austria and India) in which each metabolite is a median.
**Figure S3** Spearman correlation analysis was used to distribute 44 diverse wheat genotypes, considering secondary metabolite abundance in root exudate (identified using GC–MS) and the inhibition of AOA and AOB strains. Pairwise correlations were calculated between all 56 identified metabolites and AOA and AOB inhibition capacity. (a) Volcano plot showing the NF inhibition. (b) Volcano plot showing the NV inhibition. (c) Volcano plot showing the NM inhibition. (d) Volcano plot showing the NU inhibition. A volcano plot was generated with 56 identified primary metabolites and inhibition activity. Blue circles represent metabolites with a Spearman's correlation *R*‐value higher than 0.35, which were statistically significant (*P* ≤ 0.05), indicating a positive correlation with inhibition activity. Red circles represent metabolites with a Spearman's correlation *R*‐value lower than −0.35, which ere statistically significant (*P* ≤ 0.05), indicating a negative correlation with inhibition activity. Grey circles represent metabolites with a Spearman's correlation *R*‐value ≤0.35 to ≥ −0.35 and lacking statistical significance (*P* > 0.05). Significant correlations between metabolites and inhibition activity were detected using Spearman's correlation approach (*P* < 0.05). Dots are colour‐coded by the origin of the genotype. The size of each circle denotes the relative value of the metabolites and the corresponding wheat genotype. Only two of the highly significant negatively correlated metabolites are shown.
**Figure S4** PLS‐based network to explore the relationship between metabolites and AOA inhibition. Node colour indicates the chemical class of the metabolites. AKL, alkaloids; AAS, amino acids, amines, and derivatives; CAR, carbohydrates; NBA, nitrogenous bases; FLA, flavonoids; LIP, lipids and fatty acids; CAA, carboxylic acids; PHE, phenylpropanoids; and OTH, other that include terpenoids, vitamins and quinones. (a) The PLS network of NF inhibition and metabolites. The correlation cut‐off was 0.45, and the edge colour represents positive (blue) or negative (red) interactions. (b) The PLS network of NV inhibition and metabolites. The correlation cut‐off was 0.35, and edge colour represents positive (blue) or negative (red) interactions. Different genotype sub‐networks on the right represent the metabolite abundance in each genotype. These sub‐networks represented the highest and lowest inhibition activity of the genotypes in AOA. The node colour indicates the relative abundance of each metabolite.
**Figure S5** PLS‐based network to explore the relationship between metabolites and AOB inhibition. Node colour indicates the chemical class of the metabolites. AKL, alkaloids; AAS, amino acids, amines, and derivatives; CAR, carbohydrates; NBA, nitrogenous bases; FLA, flavonoids; LIP, lipids and fatty acids; CAA, carboxylic acids; PHE, phenylpropanoids; OTH, other that include terpenoids, vitamins and quinones. (a) The PLS network of NM inhibition and metabolites. Correlation cut‐off was 0.45 and edge colour represents positive (blue) or negative (red) interactions. (b) The PLS network of NU inhibition and metabolites. The correlation cut‐off was 0.4, and edge colour represents positive (blue) or negative (red) interactions. Different genotype sub‐networks on the right represent the metabolite abundance in each genotype. These sub‐networks represented the highest and lowest inhibition activity of the genotypes in AOB. The node colour indicates the relative abundance of each metabolite.

**Table S1** (a) GC–MS metabolomic data. Peak areas of polar metabolites are identified in the root exudates. This sheet includes annotation, family classification, InchIKey, SMILE and molecular formula. For every compound, the peak area is normalized by the root weight, and log10 + 1 transformation is included. (b) Venn analysis. Venn analysis was conducted on the GC–MS data by splitting the data based on the origin of genotypes. (c) PCA results. Explained variance by PCA, loadings and annotations. (d) Statistical analysis of GC–MS data and Spearman's correlation analysis with inhibition activity results. Spearman's correlation analysis was conducted for all the genotypes by origin and independent origin (Austrian and Indian genotypes) and for different microorganisms: AOA (NF and NV) and AOB (NM and NU).
**Table S2** (a) LC–MS metabolomic data. Peak areas of polar metabolites are identified in the root exudates. This sheet includes annotation, family classification, InchIKey, SMILE and molecular formula. (b) Venn analysis. Venn analysis was conducted by splitting the data by the origin of the genotypes. (c) PCA results for all detected features. Variance by PCA, loadings and annotations. (e) Statistical analysis of LC–MS data and Spearman's correlation analysis with inhibition activity results. Spearman's correlation analysis was conducted for all genotypes and for different microorganisms: AOA (NF and NV) and AOB (NM and NU). (d) PCA results with annotated features. Variance by PCA, loadings and annotations. (f) Statistical analysis of LC–MS data (only annotated features) for genotype origin and Spearman's correlation with inhibition activity results. Spearman's correlation analysis was conducted for all genotypes by origin and independent origin (Austrian and Indian genotypes), and for different microorganisms: AOA (NF and NV) and AOB (NM and NU).
**Table S3** (a) Statistical analysis of the inhibitory activity of root exudates on different microorganisms. This sheet includes the average and standard deviation of each genotype and inhibition capacity. Tukey's test was applied, and the different genotypes were used to determine if there were differences in the microorganism's activity. Lowercase letters indicate differences among groups after the Kruskal–Wallis test with a post‐hoc test. (b) The inhibitory activities of different microorganisms were evaluated. In the different microorganisms, the genotypes were ranked from highest to lowest inhibition capacity. This resulted in four quartiles: Q1, genotypes with high inhibition capacity; and Q4, genotypes with lowest inhibition capacity.
**Table S4** (a) PLS network of NF‐inhibition and metabolites. This sheet contains information regarding the edges and nodes of the network. (b) PLS network of NV inhibition and metabolites. This sheet contains information regarding the edges and nodes of the network. (c) PLS network of NM inhibition and metabolites. This sheet contains information regarding the edges and nodes of the network. (d) PLS network between NU inhibition and metabolites. This sheet contains information regarding the edges and nodes of the network. (e) PLS‐DA networks with quartile classification of wheat genotypes. Quartile classifications are listed in Table S6b. This sheet contains information regarding the edges and nodes of the network. (f) Evaluation of Spearman correlation *R*‐values and PLS regression values in the different microorganisms studied. To determine the metabolites with higher cumulative correlations/regression, the *R*‐values were summed. A more positive *R*‐value indicates that the metabolite has a highly positive relationship with the inhibition activity. (g) The deep learning method results and Deep Canonical Correlation Analysis (DCCA) values. Metabolites with an occurrence frequency of 5 were highlighted. The genetic algorithm (GA) was run 50 times to obtain optimal results.

## Data Availability

The data that supports the findings of this study are available in the supplementary material of this article.

## References

[pbi70248-bib-0001] Alexandratos, N. and Bruinsma, J. (2012) World agriculture towards 2030/2050: the 2012 revision.

[pbi70248-bib-0002] Alves, R.J.E. , Minh, B.Q. , Urich, T. , von Haeseler, A. and Schleper, C. (2018) Unifying the global phylogeny and environmental distribution of ammonia‐oxidising archaea based on amoA genes. Nat. Commun. 9, 1517. 29666365 10.1038/s41467-018-03861-1 PMC5904100

[pbi70248-bib-0003] Andrade, M. , Benfeito, S. , Soares, P. , Magalhães e Silva, D. , Loureiro, J. , Borges, A. , Borges, F. *et al*. (2015) Fine‐tuning of the hydrophobicity of caffeic acid: studies on the antimicrobial activity against *Staphylococcus aureus* and *Escherichia coli* . RSC Adv. 5, 53915–53925.

[pbi70248-bib-0004] Andrew, G. , Arora, R. , Bilmes, J. and Livescu, K. (2013) Deep canonical correlation analysis. In Proceedings of the 30th International Conference on International Conference on Machine Learning – Volume 28, pp. III–1247–III–1255. Atlanta, GA, USA: JMLR.org.

[pbi70248-bib-0005] Bachtsevani, E. , Papazlatani, C.V. , Rousidou, C. , Lampronikou, E. , Menkissoglu‐Spiroudi, U. , Nicol, G.W. , Karpouzas, D.G. *et al*. (2021) Effects of the nitrification inhibitor 3,4‐dimethylpyrazole phosphate (DMPP) on the activity and diversity of the soil microbial community under contrasting soil pH. Biol. Fertil. Soils 57, 1117–1135.

[pbi70248-bib-0006] Badri, D.V. , Chaparro, J.M. , Zhang, R. , Shen, Q. and Vivanco, J.M. (2013) Application of natural blends of phytochemicals derived from the root exudates of Arabidopsis to the soil reveal that phenolic‐related compounds predominantly modulate the soil microbiome. J. Biol. Chem. 288, 4502–4512. 23293028 10.1074/jbc.M112.433300 PMC3576057

[pbi70248-bib-0007] Beeckman, F. , Drozdzecki, A. , De Knijf, A. , Corrochano‐Monsalve, M. , Bodé, S. , Blom, P. , Goeminne, G. *et al*. (2023) Drug discovery‐based approach identifies new nitrification inhibitors. J. Environ. Manage. 346, 118996. 37725864 10.1016/j.jenvman.2023.118996

[pbi70248-bib-0008] Beeckman, F. , Motte, H. and Beeckman, T. (2018) Nitrification in agricultural soils: impact, actors and mitigation. Curr. Opin. Biotechnol. 50, 166–173. 29414056 10.1016/j.copbio.2018.01.014

[pbi70248-bib-0009] Brazier‐Hicks, M. , Evans, K.M. , Gershater, M.C. , Puschmann, H. , Steel, P.G. and Edwards, R. (2009) The C‐glycosylation of flavonoids in cereals. J. Biol. Chem. 284, 17926–17934. 19411659 10.1074/jbc.M109.009258 PMC2709393

[pbi70248-bib-0010] Byrnes, R.C. , Nùñez, J. , Arenas, L. , Rao, I. , Trujillo, C. , Alvarez, C. , Arango, J. *et al*. (2017) Biological nitrification inhibition by *Brachiaria grasses* mitigates soil nitrous oxide emissions from bovine urine patches. Soil Biol. Biochem. 107, 156–163.

[pbi70248-bib-0011] Canarini, A. , Kaiser, C. , Merchant, A. , Richter, A. and Wanek, W. (2019) Root exudation of primary metabolites: mechanisms and their roles in plant responses to environmental stimuli. Front. Plant Sci. 10, 157. 30881364 10.3389/fpls.2019.00157 PMC6407669

[pbi70248-bib-0012] Caranto, J.D. and Lancaster, K.M. (2017) Nitric oxide is an obligate bacterial nitrification intermediate produced by hydroxylamine oxidoreductase. Proc. Natl. Acad. Sci. USA 114, 8217–8222. 28716929 10.1073/pnas.1704504114 PMC5547625

[pbi70248-bib-0013] Cesco, S. , Neumann, G. , Tomasi, N. , Pinton, R. and Weisskopf, L. (2010) Release of plant‐borne flavonoids into the rhizosphere and their role in plant nutrition. Plant and Soil 329, 1–25.

[pbi70248-bib-0014] Chambers, M.C. , Maclean, B. , Burke, R. , Amodei, D. , Ruderman, D.L. , Neumann, S. , Gatto, L. *et al*. (2012) A cross‐platform toolkit for mass spectrometry and proteomics. Nat. Biotechnol. 30, 918–920. 23051804 10.1038/nbt.2377 PMC3471674

[pbi70248-bib-0015] Chaturvedi, P. , Pierides, I. , Lopez‐Hidalgo, C. , Garg, V. , Zhang, S. , Barmukh, R. , Bellaire, A. *et al*. (2024a) Natural variation in the chickpea metabolome under drought stress. Plant Biotechnol. J. 22, 3278–3294. 39411896 10.1111/pbi.14447 PMC11606430

[pbi70248-bib-0016] Chaturvedi, P. , Pierides, I. , Zhang, S. , Schwarzerova, J. , Ghatak, A. and Weckwerth, W. (2024b) Multiomics for crop improvement. In Frontier technologies for crop improvement( Pandey, M.K. , Bentley, A. , Desmae, H. , Roorkiwal, M. and Varshney, R.K. , eds), pp. 107–141. Singapore: Springer Nature Singapore.

[pbi70248-bib-0017] Dawar, K. , Khan, A. , Sardar, K. , Fahad, S. , Saud, S. , Datta, R. and Danish, S. (2021) Effects of the nitrification inhibitor nitrapyrin and mulch on N_2_O emission and fertilizer use efficiency using 15N tracing techniques. Sci. Total Environ. 757, 143739. 33229088 10.1016/j.scitotenv.2020.143739

[pbi70248-bib-0018] Egenolf, K. , Conrad, J. , Schöne, J. , Braunberger, C. , Beifuß, U. , Walker, F. , Nuñez, J. *et al*. (2020) Brachialactone isomers and derivatives of *Brachiaria humidicola* reveal contrasting nitrification inhibiting activity. Plant Physiol. Biochem. 154, 491–497. 32663650 10.1016/j.plaphy.2020.06.004

[pbi70248-bib-0019] Ghatak, A. , Chaturvedi, P. , Waldherr, S. , Subbarao, G.V. and Weckwerth, W. (2023) PANOMICS at the interface of root‐soil microbiome and BNI. Trends Plant Sci. 28, 106–122. 36229336 10.1016/j.tplants.2022.08.016

[pbi70248-bib-0020] Ghatak, A. , Chaturvedi, P. and Weckwerth, W. (2018) Metabolomics in plant stress physiology. Plant Genetics Mol. Biol. 164, 187–236. 10.1007/10_2017_55 29470599

[pbi70248-bib-1002] Ghatak, A. , Pierides, I. , Singh, R.K. , Srivastava, R.K. , Varshney, R.K. , Prasad, M. , Chaturvedi, P. *et al*. (2024) Millets for a sustainable future. J. Exp. Bot. 76(6), 1534–1545. 10.1093/jxb/erae507 PMC11981904 39724286

[pbi70248-bib-0021] Ghatak, A. , Schindler, F. , Bachmann, G. , Engelmeier, D. , Bajaj, P. , Brenner, M. , Fragner, L. *et al*. (2022) Root exudation of contrasting drought‐stressed pearl millet genotypes conveys varying biological nitrification inhibition (BNI) activity. Biol. Fertil. Soils 58, 291–306. 35399158 10.1007/s00374-021-01578-w PMC8938368

[pbi70248-bib-0022] Han, S. , Kim, S. , Sedlacek, C.J. , Farooq, A. , Song, C. , Lee, S. , Liu, S. *et al*. (2024) Adaptive traits of Nitrosocosmicus clade ammonia‐oxidizing archaea. MBio 15, e0216924. 39360821 10.1128/mbio.02169-24 PMC11559005

[pbi70248-bib-0023] Heuckeroth, S. , Damiani, T. , Smirnov, A. , Mokshyna, O. , Brungs, C. , Korf, A. , Smith, J.D. *et al*. (2024) Reproducible mass spectrometry data processing and compound annotation in MZmine 3. Nat. Protoc. 19, 2597–2641. 38769143 10.1038/s41596-024-00996-y

[pbi70248-bib-1004] Hu, H. , Yuan, X. , Saini, D.K. , Yang, T. , Wu, X. , Wu, R. , Liu, Z. *et al*. (2025) A panomics‐driven framework for the improvement of major food legume crops: advances, challenges, and future prospects. Hortic. Res. 12(7). 10.1093/hr/uhaf091 PMC12064956 40352287

[pbi70248-bib-0024] Huang, L. , Chakrabarti, S. , Cooper, J. , Perez, A. , John, S.M. , Daroub, S.H. and Martens‐Habbena, W. (2021) Ammonia‐oxidizing archaea are integral to nitrogen cycling in a highly fertile agricultural soil. ISME Commun 1, 19. 37938645 10.1038/s43705-021-00020-4 PMC9723749

[pbi70248-bib-0025] Iizumi, T. , Mizumoto, M. and Nakamura, K. (1998) A bioluminescence assay using Nitrosomonas europaea for rapid and sensitive detection of nitrification inhibitors. Appl. Environ. Microbiol. 64, 3656–3662. 9758781 10.1128/aem.64.10.3656-3662.1998 PMC106494

[pbi70248-bib-0026] Issifu, S. , Acharya, P. , Schone, J. , Kaur‐Bhambra, J. , Gubry‐Rangin, C. and Rasche, F. (2024) Metabolome fingerprinting reveals the presence of multiple nitrification inhibitors in biomass and root exudates of Thinopyrum intermedium. Plant Environ. Interact. 5, e70012. 39345302 10.1002/pei3.70012 PMC11431351

[pbi70248-bib-0027] Kanellopoulos, A.E. , Malits, A. , Ribeiro, H. , Kerou, M. , Ghatak, A. , Chaturvedi, P. , Weckwerth, W. *et al*. (2024) A fast‐track, high‐throughput screening platform for biological nitrification inhibitors discovery based on soil relevant ammonia oxidizing strains. *bioRxiv* . 2024.2012.2003.626636.

[pbi70248-bib-0028] Kaur‐Bhambra, J. , Wardak, D.L.R. , Prosser, J.I. and Gubry‐Rangin, C. (2022) Revisiting plant biological nitrification inhibition efficiency using multiple archaeal and bacterial ammonia‐oxidising cultures. Biol. Fertil. Soils 58, 241–249.

[pbi70248-bib-0029] Kolovou, M. , Panagiotou, D. , Susse, L. , Loiseleur, O. , Williams, S. , Karpouzas, D.G. and Papadopoulou, E.S. (2023a) Assessing the activity of different plant‐derived molecules and potential biological nitrification inhibitors on a range of soil ammonia‐ and nitrite‐oxidizing strains. Appl. Environ. Microbiol. 89, e0138023. 37916825 10.1128/aem.01380-23 PMC10686072

[pbi70248-bib-0030] Kolovou, M. , Panagiotou, D. , Süße, L. , Loiseleur, O. , Williams, S. , Karpouzas Dimitrios, G. and Papadopoulou Evangelia, S. (2023b) Assessing the activity of different plant‐derived molecules and potential biological nitrification inhibitors on a range of soil ammonia‐ and nitrite‐oxidizing strains. Appl. Environ. Microbiol. 89, e01380‐01323. 37916825 10.1128/aem.01380-23 PMC10686072

[pbi70248-bib-0031] Koops, H.P. , B?her, B. , M?r, U.C. , Pommerening‐R? A. and Stehr, G. (1991) Classification of eight new species of ammonia‐oxidizing bacteria: *Nitrosomonas communis* sp. nov., *Nitrosomonas ureae* sp. nov., *Nitrosomonas aestuarii* sp. nov., *Nitrosomonas marina* sp. nov., *Nitrosomonas nitrosa* sp. nov., *Nitrosomonas eutropha* sp. nov., *Nitrosomonas oligotropha* sp. nov. and *Nitrosomonas halophila* sp. nov. Microbiology 137, 1689–1699.

[pbi70248-bib-0032] Lambora, A. , Gupta, K. and Chopra, K. (2019) Genetic algorithm – a literature review.

[pbi70248-bib-0033] Lan, M. , Yin, Q. , Wang, J. , Li, M. , Li, Y. and Li, B. (2023) Heterotrophic nitrification‐aerobic denitrification performance of a novel strain, *Pseudomonas* sp. B‐1, isolated from membrane aerated biofilm reactor. Environ. Res. 220, 115199. 36592808 10.1016/j.envres.2022.115199

[pbi70248-bib-0034] Lan, T. , Li, M. , He, X. , Deng, O. , Zhou, W. , Luo, L. , Chen, G. *et al*. (2022) Effects of synthetic nitrification inhibitor (3,4‐dimethylpyrazole phosphate; DMPP) and biological nitrification inhibitor (methyl 3‐(4‐hydroxyphenyl) propionate; MHPP) on the gross N nitrification rate and ammonia oxidizers in two contrasting soils. Biol. Fertil. Soils 58, 333–344.

[pbi70248-bib-0035] Lancaster, K.M. , Caranto, J.D. , Majer, S.H. and Smith, M.A. (2018) Alternative bioenergy: updates to and challenges in nitrification metalloenzymology. Joule 2, 421–441.

[pbi70248-bib-0036] Lee, U.J. , Gwak, J.H. , Choi, S. , Jung, M.Y. , Lee, T.K. , Ryu, H. , Imisi Awala, S. *et al*. (2024) “Ca. Nitrosocosmicus” members are the dominant archaea associated with plant rhizospheres. mSphere 9, e0082124. 39530672 10.1128/msphere.00821-24 PMC11656794

[pbi70248-bib-0037] Lehmann, J. , Bossio, D.A. , Kögel‐Knabner, I. and Rillig, M.C. (2020) The concept and future prospects of soil health. Nature Reviews Earth & Environment 1, 544–553. 10.1038/s43017-020-0080-8 PMC7116140 33015639

[pbi70248-bib-0038] Lu, Y. , Hua, Y. , Lv, N. , Zu, W. , Kronzucker, H.J. , Dong, G. and Shi, W. (2022) Syringic acid from rice roots inhibits soil nitrification and N(2)O emission under red and paddy soils but not a calcareous soil. Front. Plant Sci. 13, 1099689. 36605956 10.3389/fpls.2022.1099689 PMC9808040

[pbi70248-bib-0039] Lu, Y. , Zhang, X. , Jiang, J. , Kronzucker, H.J. , Shen, W. and Shi, W. (2019) Effects of the biological nitrification inhibitor 1,9‐decanediol on nitrification and ammonia oxidizers in three agricultural soils. Soil Biol. Biochem. 129, 48–59.

[pbi70248-bib-0040] Majumdar, D. (2002) Suppression of nitrification and N_2_O emission by karanjin—a nitrification inhibitor prepared from karanja (*Pongamia glabra* Vent.). Chemosphere 47, 845–850. 12079079 10.1016/s0045-6535(01)00287-9

[pbi70248-bib-0041] Matson, P.A. , Naylor, R. and Ortiz‐Monasterio, I. (1998) Integration of environmental, agronomic, and economic aspects of fertilizer management. Science 280, 112–115. 9525856 10.1126/science.280.5360.112

[pbi70248-bib-0042] McGeough, K.L. , Watson, C.J. , Müller, C. , Laughlin, R.J. and Chadwick, D.R. (2016) Evidence that the efficacy of the nitrification inhibitor dicyandiamide (DCD) is affected by soil properties in UK soils. Soil Biol. Biochem. 94, 222–232.

[pbi70248-bib-1003] Mishra, S. , Srivastava, A.K. , Khan, A.W. , Tran, L.‐S.P. and Nguyen, H.T. (2024) The era of panomics‐driven gene discovery in plants. Trends Plant Sci. 29(9), 995–1005. 38658292 10.1016/j.tplants.2024.03.007

[pbi70248-bib-0043] Nardi, P. , Akutsu, M. , Pariasca‐Tanaka, J. and Wissuwa, M. (2013) Effect of methyl 3‐4‐hydroxyphenyl propionate, a Sorghum root exudate, on N dynamic, potential nitrification activity and abundance of ammonia‐oxidizing bacteria and archaea. Plant and Soil 367, 627–637.

[pbi70248-bib-0044] Nardi, P. , Laanbroek, H.J. , Nicol, G.W. , Renella, G. , Cardinale, M. , Pietramellara, G. , Weckwerth, W. *et al*. (2020) Biological nitrification inhibition in the rhizosphere: determining interactions and impact on microbially mediated processes and potential applications. FEMS Microbiol. Rev. 44, 874–908. 32785584 10.1093/femsre/fuaa037

[pbi70248-bib-0045] Oburger, E. and Jones, D.L. (2018) Sampling root exudates – Mission impossible? Rhizosphere 6, 116–133.

[pbi70248-bib-0046] O'Sullivan, C. , Fillery, I. , Roper, M. and Richards, R. (2016) Identification of several wheat landraces with biological nitrification inhibition capacity. Plant Soil 404, 61–74.

[pbi70248-bib-0047] Otaka, J. , Subbarao, G.V. , Ono, H. and Yoshihashi, T. (2022) Biological nitrification inhibition in maize—isolation and identification of hydrophobic inhibitors from root exudates. Biol. Fertil. Soils 58, 251–264.

[pbi70248-bib-0048] Papadopoulou, E.S. , Bachtsevani, E. , Lampronikou, E. , Adamou, E. , Katsaouni, A. , Vasileiadis, S. , Thion, C. *et al*. (2020) Comparison of novel and established nitrification inhibitors relevant to agriculture on soil ammonia‐ and nitrite‐oxidizing isolates. Front. Microbiol. 11, 581283. 33250872 10.3389/fmicb.2020.581283 PMC7672009

[pbi70248-bib-0049] Pluskal, T. , Castillo, S. , Villar‐Briones, A. and Oresic, M. (2010) MZmine 2: modular framework for processing, visualizing, and analyzing mass spectrometry‐based molecular profile data. BMC Bioinformatics 11, 395. 20650010 10.1186/1471-2105-11-395 PMC2918584

[pbi70248-bib-1005] R Core Team (2024) R: a language and environment for statistical computing. Vienna, Austria: R Foundation for Statistical Computing.

[pbi70248-bib-0050] Rasmussen, I.S. and Thorup‐Kristensen, K. (2016) Does earlier sowing of winter wheat improve root growth and N uptake? Field Crop Res 196, 10–21.

[pbi70248-bib-0051] Raun, W.R. and Johnson, G.V. (1999) Improving nitrogen use efficiency for cereal production. Agron. J. 91, 357–363.

[pbi70248-bib-0052] Raza, A. , Li, Y. , Prakash, C.S. and Hu, Z. (2025) Panomics to manage combined abiotic stresses in plants. Trends Plant Sci. 10.1016/j.tplants.2025.03.001 40148151

[pbi70248-bib-0053] Reyes, C. , Hodgskiss, L.H. , Baars, O. , Kerou, M. , Bayer, B. , Schleper, C. and Kraemer, S.M. (2020) Copper limiting threshold in the terrestrial ammonia oxidizing archaeon *Nitrososphaera viennensis* . Res. Microbiol. 171, 134–142. 31991171 10.1016/j.resmic.2020.01.003

[pbi70248-bib-0054] Rohart, F. , Gautier, B. , Singh, A. and Le Cao, K.A. (2017) mixOmics: An R package for 'omics feature selection and multiple data integration. PLoS Comput. Biol. 13, e1005752. 29099853 10.1371/journal.pcbi.1005752 PMC5687754

[pbi70248-bib-0055] Roychowdhury, R. , Ghatak, A. , Kumar, M. , Samantara, K. , Weckwerth, W. and Chaturvedi, P. (2024) Accelerating wheat improvement through trait characterization: advances and perspectives. Physiol. Plant. 176, e14544. 39360330 10.1111/ppl.14544

[pbi70248-bib-0056] Schindler, F. , Fragner, L. , Herpell, J.B. , Berger, A. , Brenner, M. , Tischler, S. , Bellaire, A. *et al*. (2021) Dissecting metabolism of leaf nodules in *Ardisia crenata* and *Psychotria punctata* . Front. Mol. Biosci. 8, 683671. 34395523 10.3389/fmolb.2021.683671 PMC8362603

[pbi70248-bib-0057] Sedlacek, C.J. , McGowan, B. , Suwa, Y. , Sayavedra‐Soto, L. , Laanbroek, H.J. , Stein, L.Y. , Norton, J.M. *et al*. (2019) A physiological and genomic comparison of Nitrosomonas cluster 6a and 7 ammonia‐oxidizing bacteria. Microb. Ecol. 78, 985–994. 30976841 10.1007/s00248-019-01378-8

[pbi70248-bib-0058] Shannon, P. , Markiel, A. , Ozier, O. , Baliga, N.S. , Wang, J.T. , Ramage, D. , Amin, N. *et al*. (2003) Cytoscape: a software environment for integrated models of biomolecular interaction networks. Genome Res. 13, 2498–2504. 14597658 10.1101/gr.1239303 PMC403769

[pbi70248-bib-0059] Shinn, M.B. (1941) Colorimetric method for determination of nitrate. Industrial & Engineering Chemistry Analytical Edition 13, 33–35.

[pbi70248-bib-0060] Skinner, F.A. and Walker, N. (1961) Growth of *Nitrosomonas europaea* in batch and continuous culture. Arch. Mikrobiol. 38, 339–349. 10.1007/BF00446615 4884200

[pbi70248-bib-0061] Strehmel, N. , Böttcher, C. , Schmidt, S. and Scheel, D. (2014) Profiling of secondary metabolites in root exudates of *Arabidopsis thaliana* . Phytochemistry 108, 35–46. 25457500 10.1016/j.phytochem.2014.10.003

[pbi70248-bib-0062] Subbarao, G.V. , Ishikawa, T. , Ito, O. , Nakahara, K. , Wang, H.Y. and Berry, W.L. (2006) A bioluminescence assay to detect nitrification inhibitors released from plant roots: a case study with *Brachiaria humidicola* . Plant and Soil 288, 101–112.

[pbi70248-bib-0063] Subbarao, G.V. , Kishii, M. , Bozal‐Leorri, A. , Ortiz‐Monasterio, I. , Gao, X. , Ibba, M.I. , Karwat, H. *et al*. (2021) Enlisting wild grass genes to combat nitrification in wheat farming: a nature‐based solution. Proc. Natl. Acad. Sci. USA 118, e2106595118. 34426500 10.1073/pnas.2106595118 PMC8536370

[pbi70248-bib-0064] Subbarao, G.V. , Nakahara, K. , Hurtado, M.P. , Ono, H. , Moreta, D.E. , Salcedo, A.F. , Yoshihashi, A.T. *et al*. (2009) Evidence for biological nitrification inhibition in Brachiaria pastures. Proc. Natl. Acad. Sci. USA 106, 17302–17307. 19805171 10.1073/pnas.0903694106 PMC2752401

[pbi70248-bib-0065] Subbarao, G.V. , Nakahara, K. , Ishikawa, T. , Ono, H. , Yoshida, M. , Yoshihashi, T. , Zhu, Y.Y. *et al*. (2013) Biological nitrification inhibition (BNI) activity in sorghum and its characterization. Plant and Soil 366, 243–259.

[pbi70248-bib-0066] Subbarao, G.V. , Rondon, M. , Ito, O. , Ishikawa, T. , Rao, I.M. , Nakahara, K. , Lascano, C. *et al*. (2007a) Biological nitrification inhibition (BNI)—is it a widespread phenomenon? Plant and Soil 294, 5–18.

[pbi70248-bib-0067] Subbarao, G.V. and Searchinger, T.D. (2021) Opinion: A “more ammonium solution” to mitigate nitrogen pollution and boost crop yields. Proc. Natl. Acad. Sci. USA 118, e2107576118. 34039714 10.1073/pnas.2107576118 PMC8179215

[pbi70248-bib-0068] Subbarao, G.V. , Tomohiro, B. , Masahiro, K. , Osamu, I. , Samejima, H. , Wang, H.Y. , Pearse, S.J. *et al*. (2007b) Can biological nitrification inhibition (BNI) genes from perennial *Leymus racemosus* (Triticeae) combat nitrification in wheat farming? Plant and Soil 299, 55–64.

[pbi70248-bib-0069] Subbarao, G.V. , Yoshihashi, T. , Worthington, M. , Nakahara, K. , Ando, Y. , Sahrawat, K.L. , Rao, I.M. *et al*. (2015) Suppression of soil nitrification by plants. Plant Sci. 233, 155–164. 25711823 10.1016/j.plantsci.2015.01.012

[pbi70248-bib-0070] Sumner, L.W. , Urbanczyk‐Wochniak, E. and Broeckling, C.D. (2007) Metabolomics data analysis, visualization, and integration. Methods Mol. Biol. 406, 409–436. 18287705 10.1007/978-1-59745-535-0_20

[pbi70248-bib-0071] Sun, L. , Lu, Y. , Yu, F. , Kronzucker, H.J. and Shi, W. (2016) Biological nitrification inhibition by rice root exudates and its relationship with nitrogen‐use efficiency. New Phytol. 212, 646–656. 27292630 10.1111/nph.14057

[pbi70248-bib-0072] Sutton, M. , Raghuram, N. , Adhya, T. , Baron, J. , Cox, C. , Vries, W. , Hicks, K. *et al*. (2019) The Nitrogen Fix: From nitrogen cycle pollution to nitrogen circular economy.

[pbi70248-bib-0073] Thorup‐Kristensen, K. , Salmerón Cortasa, M. and Loges, R. (2009) Winter wheat roots grow twice as deep as spring wheat roots, is this important for N uptake and N leaching losses? Plant and Soil 322, 101–114.

[pbi70248-bib-0074] Tilman, D. , Cassman, K.G. , Matson, P.A. , Naylor, R. and Polasky, S. (2002) Agricultural sustainability and intensive production practices. Nature 418, 671–677. 12167873 10.1038/nature01014

[pbi70248-bib-0075] Vajrala, N. , Martens‐Habbena, W. , Sayavedra‐Soto, L.A. , Schauer, A. , Bottomley, P.J. , Stahl, D.A. and Arp, D.J. (2013) Hydroxylamine as an intermediate in ammonia oxidation by globally abundant marine archaea. Proc. Natl. Acad. Sci. 110, 1006–1011. 23277575 10.1073/pnas.1214272110 PMC3549078

[pbi70248-bib-0076] Valentinuzzi, F. , Cesco, S. , Tomasi, N. and Mimmo, T. (2015) Influence of different trap solutions on the determination of root exudates in *Lupinus albus* L. Biol. Fertil. Soils 51, 757–765.

[pbi70248-bib-0077] Wang, X. , Zhang, J. , Lu, X. , Bai, Y. and Wang, G. (2024) Two diversities meet in the rhizosphere: root specialized metabolites and microbiome. J. Genet. Genomics 51, 467–478. 37879496 10.1016/j.jgg.2023.10.004

[pbi70248-bib-0078] Wang, Z.‐L. , Gao, H.‐M. , Wang, S. , Zhang, M. , Chen, K. , Zhang, Y.‐Q. , Wang, H.‐D. *et al*. (2020) Dissection of the general two‐step di‐C−glycosylation pathway for the biosynthesis of (iso)schaftosides in higher plants. Proc. Natl. Acad. Sci. 117, 30816–30823. 33199630 10.1073/pnas.2012745117 PMC7720146

[pbi70248-bib-1001] Weckwerth, W. , Chaturvedi, P. , Ghatak, A. , Kerou, M. , Garg, V. , Bohra, A. , Subbarao, G.V. *et al*. (2025) Natural variation of the holobiont for sustainable agroecosystems. Trends Plant Sci. 27, S1360–S1385(25)00134‐7. 10.1016/j.tplants.2025.05.006 40579258

[pbi70248-bib-0079] Weckwerth, W. , Ghatak, A. , Bellaire, A. , Chaturvedi, P. and Varshney, R.K. (2020) PANOMICS meets germplasm. Plant Biotechnol. J. 18, 1507–1525. 32163658 10.1111/pbi.13372 PMC7292548

[pbi70248-bib-0080] Weckwerth, W. , Wenzel, K. and Fiehn, O. (2004) Process for the integrated extraction identification, and quantification of metabolites, proteins and RNA to reveal their co‐regulation in biochemical networks. Proteomics 4, 78–83. 14730673 10.1002/pmic.200200500

[pbi70248-bib-0081] Winiwarter, W. , Höglund‐Isaksson, L. , Klimont, Z. , Schoepp, W. and Amann, M. (2018) Technical opportunities to reduce global anthropogenic emissions of nitrous oxide. Environ. Res. Lett. 13, 014011.

[pbi70248-bib-0082] Wolt, J.D. (2004) A meta‐evaluation of nitrapyrin agronomic and environmental effectiveness with emphasis on corn production in the Midwestern USA. Nutr. Cycl. Agroecosyst. 69, 23–41.

[pbi70248-bib-0083] Zhang, S. , Ghatak, A. , Bazargani, M.M. , Bajaj, P. , Varshney, R.K. , Chaturvedi, P. , Jiang, D. *et al*. (2021) Spatial distribution of proteins and metabolites in developing wheat grain and their differential regulatory response during the grain filling process. Plant J. 107, 669–687. 34227164 10.1111/tpj.15410 PMC9291999

[pbi70248-bib-0084] Zhang, S. , Ghatak, A. , Mohammadi Bazargani, M. , Kramml, H. , Zang, F. , Gao, S. , Ramšak, Ž. *et al*. (2024) Cell‐type proteomic and metabolomic resolution of early and late grain filling stages of wheat endosperm. Plant Biotechnol. J. 22, 555–571. 38050335 10.1111/pbi.14203 PMC12047074

