# Supplementary material for: Natural variation of the wheat root exudate metabolome and its influence on biological nitrification inhibition activity
Source: Plant Biotechnol J. 2025 Jul 21;23(11):4755–72. doi: 10.1111/pbi.70248 (PMC12576471; doi:10.1111/pbi.70248)
Supplement: Supplementary file 1 — Appendix S1 Details and validation of the Fast‐track screening system using SNIs and BNIs, metabolomics analysis (GC‐MS/LC‐MS) and deep learning algorithm. [file PBI-23-4755-s002.docx]

**Appendix S1.** Details and validation of the fast-track screening system using SNIs and BNIs, metabolomics analysis (GC-MS/LC-MS) and deep learning algorithm.

*This document contains figures, tables and references.

## **High-throughput nitrification inhibition screening**

## **AOM strains and growth conditions**

*Nitrosospira multiformis* ATCC25196 and *Nitrosomonas ureae* Nm10 (provided by Graeme Nicol, University of Lyon, France), were cultivated in Skinner and Walker (SW) medium (1). *Nitrosomonas communis* Nm2 (obtained from Lisa Stein, University of Alberta, Canada) was grown in NCOM medium 2), adapted to a 3mM NH_4_^+^ substrate, with phenol red as pH indicator. All AOB cultures were incubated statically, in the dark, at 28^o^C, in 30 mL Sterilin™ sterile polystyrene bottles (ThermoFischer Scientific, Waltham, Massachusetts, USA) at a working volume of 20 mL.

Axenic liquid cultures of the terrestrial AOA *Nitrososphaera viennensis* EN76 and “*Candidatus* Nitrosocosmicus franklandianus C13” were grown in freshwater medium (FWM) buffered with HEPES to pH of 7.5 and supplied with 2mM NH_4_^+^ following Reyes et al. (3) with slight modifications. *N. viennensis* was incubated without vitamin solution, but with 1 mM pyruvate to scavenge reactive oxygen species. “*Ca.* N. franklandianus” was cultured with 1mM thiamine HCl and without pyruvate. The AOA were grown in the dark in sterile Duran® borosilicate bottles or 30 mL polystyrene tubes, filled up to two-thirds and shaken at 80 rpm.

## **Development of the fast-track screening system**

The workflow of the AOB and AOA fast – track system is presented in main Figure 3. Regarding AOB, three 60-mL fractions of cultures at the late logarithmic phase were filtered through hydrophilic 0.22 μm polyethersulphone (PES) membrane filters (Frisenette ApS, Knebel, Denmark), the filter was rotated upside-down and bacterial cells were detached through tapping of the filter for 60 sec. Subsequently, 2 mL of sterile SW medium for *N. multiformis* and *N. ureae*, or sterile NCOM medium for *N. communis*, were passed through the filter to resuspend the harvested cells. The filtrates were centrifugated at 11,000 g for 10 min at room temperature. 1.8 mL of the supernatant was discarded and all inocula were combined into a final volume of 2 mL. This concentrated inoculum was then diluted with sterile SW or NCOM medium, at ratios of 1:10 for *N. multiformis* and *N. communis* and 0.7:10 for *N. ureae*, gently mixed and 200 μL of the culture were dispersed to the wells of a cellGrade^TM^ 96 – well plate (BRAND GMBH & CO KG, Wertheim, Germany). The plate was incubated with a lid, under the same conditions as the respective AOB cultures. Nitrite production was determined colourimetrically at 540 nm by diazotizing and coupling with Griess reagent (4) at specific time-points for 24 hours, or until sufficient consumption of the ammonium substrate in the control treatments (0.1% v/v ddH_2_O or DMSO) was observed. Samples for measuring the abundance of the *amoA* gene were collected at specific time-points as outlined in main Figure 3.

For AOA, *N. viennensis* cultures at the late logarithmic phase were centrifuged at 21,000 g for 30 min at 4°C. The supernatant was discarded, and the pellets resuspended with FWM and centrifuged again to wash out residual nitrite. Again, the supernatant was discarded, and the pellets were resuspended in FWM and kept at 4°C until further processing. “*Ca.* N. franklandianus*”* cultures were concentrated on Millipore® sterile hydrophilic 0.22 μm mixed cellulose esters (MCE) membrane filters using a sterile filtration unit. The cells on the filter were rinsed with FWM to wash the residual nitrite, the filter was placed into a sterile centrifuge tube and FWM was added to achieve the desired cell density. The tube was shaken to detach the cells from the filter, the filter was removed, and the culture concentrate was stored at 4°C until further processing. AOA culture concentrates were allowed to prewarm at 42°C before 200 µL were added in the wells of a 96-well plate, which was incubated with a lid, statically, at 42°C. Samples were taken at the beginning of incubation and at several time points, until approximately half of the provided NH_4_^+^ substrate was consumed in the control treatments (0.1% v/v ultra-pure water or 0.02% v/v DMSO), to quantify NO_2_^-^ production via a scaled down Griess reaction method. At the beginning of incubation, samples for determination of cell density using flow cytometry were taken.

## **Validation of the fast-track screening system with known SNIs and BNIs**

To evaluate the performance of the fast-track screening system a range of known SNIs and BNIs were used. The SNIs included the hydrophilic 3,4-dimethylpyrazole (phosphate) (DMPP) (5) and the hydrophobic 2-chloro-6-(trichloromethyl) pyridine (nitrapyrin) (6) and 1,2-dihydro-6-ethoxy-2,2,4-trimethylquinoline (ethoxyquin) (7). The BNIs tested included, the hydrophilic methyl 3-(4-hydroxyphenyl) propionate (MHPP) (8) and the hydrophobic sakuranetin (9) and 1,9-decanediol (10). Providers and purities of analytical standards are summarised in Table 1 below. The selected NIs were utilized at a range of concentrations, expected to impose from slight to full inhibition of the activity of AOM strains. Different concentration levels were employed for the different reporter strains (Table 2), based on their sensitivity as depicted from earlier studies (7,11).

For AOB, 200 μL of the concentrated culture were added at each well, followed by addition of each inhibitor from stock solutions. Control cultures of 0.1% v/v ddH_2_O and 0.1% v/v DMSO were included as well. The final DMSO concentration was not expected to impose significant inhibition on AOB (12,13). Controls and hydrophilic NIs (DMPP and MHPP) were initially diluted in sterile growth medium before adding 20 μL to the wells. For the rest of the NIs, 0.2 μL were applied directly to the wells, followed by addition of 19.8 μL of growth medium. Triplicate cultures for each inhibitor x concentration combination were prepared, with each well representing one biological replication. Nitrite determination at different time points was used to determine the Ammonia Oxidation Inhibition percentage (AOI%) and the EC_50_ value.

For AOA, 200µL concentrated cultures were incubated with the addition of the respective NI or control in triplicates in 96-well plates for 3-5 hours. Control cultures of 0.1% v/v ddH_2_O and 0.02% v/v DMSO were also established, following the DMSO tolerance limit for the highly sensitive concentrated AOA cultures. NI stocks in DMSO were diluted with filter-sterilized ddH_2_O to achieve a range of NI concentrations and not to exceed 1% (v/v) agent in the culture. Nitrite determination at a single time point was used to determine the Ammonia Oxidation Inhibition percentage (AOI%) and the EC_50_ value.

## **Cell density determination**

For AOB, DNA from collected samples was extracted using the NucleoSpin® Tissue kit (Macherey – Nagel GmbH & Co. KG, Düren, Germany), following the manufacturer’s protocol. The *amoA* gene was quantified via qPCR in a Biorad (Hercules, California, USA) CFX Real–Time PCR system, at 40 cycles, with the amoA-1F/amoA-2R set of primers (14) as described in Bachtsevani et al. (15). qPCR efficiency was > 80% and the R^2^ of the standard curve was > 0.98. For cell density determination, it was considered that the *amoA* gene is present in three copies in *N. multiformis* (16) and *N. ureae* (17), and in two copies in *N. communis* (18).

For AOA, cell abundance was quantified by flow cytometry as in Malits et al. (19). Samples were analysed using a FACSAria II (BD Biosciences, Franklin Lakes, New Jersey, USA) flow cytometer and AOA populations were discriminated and enumerated according to their specific signature in plots of 90° light scatter versus green DNA fluorescence.

## **Statistical approach for BNI assay**

Data processing and visualisation were conducted using the RStudio software (20). Plot generation utilized either the “ggplot2” package v3.4.2 (21) or base R functions. In the duration of the analyses, the R packages “readxl” v1.4.3 (22), “dplyr” v1.1.4 (23) and “tibble” v3.2.1 (24) have been used for general data processing purposes, and the “ggplot2” v3.5.0 (21), “ggpubr” v0.6.0 (25) and “patchwork” v1.2.0 (26) R packages have been used for the generation of figures.

Modelling of the activity of AOB routine cultures was performed by fitting a single sigmoidal curve to nitrite data obtained over consecutive generations. The modelling employed the “sicegar” package v0.2.4 (27), using the equation that follows in [1], according to (27). AOB μ_max_ was determined from the semi logarithmic plot of nitrite concentration against time, as performed in others (28,29). Activity rates of AOB concentrated cultures were calculated using the same approach, utilizing data from the entire incubation period.

$$I\left( t \right)=f_{sig}\left( t \right)= \frac{I_{max}}{1+ e^{-a_{1}(t-t_{mid})}} [1]$$

EC_50_ values represent the concentrations that inhibit ammonia oxidation activity by 50% compared to the respective control treatments. Ammonia Oxidation Inhibition percentage (AOI%) for pure NIs refers to the proportional inhibition of AOM activity compared to the control. For dose-response curves fitting a traditional single sigmoidal pattern, normalized nitrite production data were used with the “drc” v3.0.1 package (30) to determine EC_50_ values, as described in Papadopoulou et al. (7). EC_50_ values were generated by the algorithm for each time point, and an average value ± the standard error was calculated from those with a satisfactory p – value and R^2^ ≥ 0.95. For dose schemes that produced a linear or polynomial AOB response, the slopes of the AOM activity linear regression curves at each dose level were utilised to determine the AOI% for each inhibitor concentration, with calculations performed as shown in [2]. In this case, the AOI% values were plotted against the respective concentrations. If a satisfactory linear regression (adjusted R^2^ > 0.9, p – value < 0.05) could be produced with at least 4 out of 5 data points, the EC_50_ concentration was calculated from the equation of the best linear regression, for y = 50. A similar approach was utilized by Kaur – Bhambra et al. (29) with μ_max_ replacing the AOI%. The standard error of the EC_50_ value was considered to coincide with the standard error produced for the unknown predictor value by the *calibrate ()* function of the “investr” v1.4.2 R package (31) at a 95% confidence level, when the observed variable was set to 50. If no satisfactory linear relationship was observed, a third-degree polynomial fit was utilized instead, with the EC_50_ calculated from the equation of the curve for y = 50. The standard error was estimated through the Delta Method. Briefly, the first derivative of the 3^rd^ degree polynomial equation at the root for y = 50 was calculated and the sensitivity of the polynomial function with respect to its coefficients at the root was computed, in order to calculate variance propagation and deduct the standard error.

$$AOI \left( \% \right)=\frac{a_{control}- a_{i}}{a_{control}} x 100\% [2]$$

For AOA data, where one time – point was utilised, AOI% values calculated using the equations in [3-5] were plotted against the respective concentrations and a similar approach utilising a linear, a 3^rd^ degree polynomial or a log_10_ fit, with the model decided based on the best fit. Standard errors were estimated as described above.

AOI% for wheat root exudates refers to the proportional inhibition of AOM activity compared to the control. It was calculated for each treatment as follows in [5], after normalising all nitrite production values [3-4].

$${{Normalised NO}_{2}}_{control}=\frac{{{NO}_{2}}_{control}}{{{Average NO}_{2}}_{control}} [3]$$

$${{Normalised NO}_{2}}_{sample}=\frac{{{NO}_{2}}_{sample}}{{{Average NO}_{2}}_{control}} [4]$$

$$AOI \%= {{- (Normalised NO}_{2}}_{sample}-{{Normaised NO}_{2}}_{control}) [5]$$

For data distribution validation, the Shapiro-Wilk normality test was conducted, supplemented by the respective histogram and q-q plot using the “stats” base package in the R Software. Kruskal-Wallis test and the Dunn’s post-hoc test with the Bonferroni p-value adjustment method was performed using the “agricolae” package v1.3.7 (32) and the “rstatix” v0.7.2 (33) R packages for comparing cell density means obtained from qPCR. Differences in the normalised nitrite values for the different SNI and BNI doses tested against AOA were evaluated with Kruskal-Wallis tests, and the Dunn’s post-hoc test with the Bonferroni p-value adjustment method, employing the “agricolae” package v1.3.7 (32) and the “rstatix” v0.7.2 (33) R packages. The heatmap of the BNI and SNI EC_50_ values were generated with the utilisation of the “ComplexHeatmap” v2.14.0 (34) and “dendextend” v1.17.1 (35) R packages, with both column and row dendrograms based on hierarchical clustering and Euclidean distances. Comparisons of these mean EC_50_ values per NI and AOM strain were performed utilising the respective means and standard errors and calculating the 95% confidence interval range based on the equation in [6].

$$mean \mathrm{EC}_{50}-1.96 \times standard error \leq\mathrm{EC}_{50}\leq mean \mathrm{EC}_{50}+1.96 \times standard error [6]$$

Differences in the AOI% values of the root exudates were evaluated per AOM strain with Kruskal-Wallis tests, and the Dunn’s post-hoc test with the Bonferroni p-value adjustment method employing the “agricolae” package v1.3.7 (32) and the “rstatix” v0.7.2 (33) R packages. The effect of the AOM strain on the RE AOI% values was assessed through a Kruskal-Wallis test and the Dunn’s post-hoc test with the Bonferroni p-value adjustment method, using the “agricolae” package v1.3.7 (32) and the “rstatix” v0.7.2 (33) R packages. The effect of the AOM domain and wheat genotype origin on the RE AOI% values were assessed via a Wilcoxon test. The heatmap of the RE AOI% values was generated with the utilisation of the “ComplexHeatmap” v2.14.0 (34) and “dendextend” v1.17.1 (35) R packages, with both column and row dendrograms based on hierarchical clustering and Euclidean distances. Finally, Principal Component Analysis (PCA) to identify different inhibitory patterns, was conducted after AOI% values were scaled using the “vegan” v2.6.4 R package (36) and the plot was generated using the “factoextra” v1.0.7 R package (37).

**Metabolome Profiling of root exudates**

**Gas chromatography coupled to mass spectrometry (GC–MS)**

Metabolite extraction was performed according to Weckwerth and co-workers (Weckwerth et al., 2004), with slight modifications. 2 mL of root exudate solution was further concentrated to 200 µL using a speedvac (SCANVAC Cool Safe 110-4, Speed Vacuum concentrator, Labogene). The metabolites were extracted by addition of 750 µL pre-chilled extraction solution MeOH: water (MW) (2.5: 1.5 v: v) to the concentrated exudates, the mixture was vortexed thoroughly, followed by incubation on ice for 15 min with periodic vortexing. After incubation the samples were centrifuged at 20,000g for 4 min at 4 °C, and the supernatant was transferred to a new safe lock eppendorf tube. The remaining pellet was washed once again with 250 µL of pre-chilled MW solution. The supernatant was pooled with the previously collected MW solution, 300 µL water was added, and the samples were mixed and centrifuged for 2 min at 20,000g at 25 °C. The supernatant was collected and the samples were concentrated completely using a speedvac (SCANVAC Cool Safe 110-4, Speed Vacuum concentrator, Labogene). All analysis steps, including sample derivatization and GC-TOF-MS (gas chromatography coupled to time-of-flight mass spectrometry), were carried out as previously described (Weckwerth et al., 2004; Obermeyer et al., 2013)

**Liquid chromatography coupled to mass spectrometry (LC–MS)**

Root exudate analysis was performed using an UltiMate 3000 UHPLC system coupled to an Orbitrap Elite mass spectrometer (Thermo Fisher Scientific) according to the method described by Schindler and co-workers, with slight modifications (Schindler et al., 2021). Extracts were separated on an UHPLC column (Accucore Vanquish C18+, 100 × 2.1 mm; 1.5 μm particle size, Thermo Fisher Scientific, Country), equipped with a guard column (Accucore Defender guards pk4 C18, 10 × 2.1 mm, 2.6 μm particle size, Thermo Fisher Scientific, Country). The mobile phase system consisted of a mixture of solvent A, an aqueous solution of 0.1% (v/v) formic acid (FA), and solvent B, MeOH, containing 0.1% (v/v) FA. A gradient elution method was used for the analysis, 0–5 min 99% A, 5–65 min linear gradient to 1% A, 65–104 min 1% A, 104–105 min linear gradient to 99% A and 105–125 min 99% A. Sample volumes of 10 µL were injected at a flowrate of 0.1 ml/min using a 25 µL sample loop. To ensure system equilibrium, a two-column washing step was performed before the samples were measured by injecting a solution composed of 98% (v/v) ddH_2_O, 2% (v/v) MeOH), and 0.1% (v/v) FA using the following gradient: 0–2 min 99% A; 2–22 min linear gradient to 1% A; 22–41 min 1% A; 41–42 min linear gradient to 99% A; and 42–62 min 99% A. After each measurement, the same column washing step was performed.

MS analysis was performed in the positive mode using an Orbitrap Elite mass spectrometer (Thermo Fisher Scientific). The following parameters were used: resolution, 60,000; spray voltage, 3.5 kV; capillary temperature, 350°C; source heater temperature, 300°C; sheath gas, 35 arbitrary units; auxiliary gas, 10 arbitrary units. The mass scanning range of the MS1 full can was set at 50–1,800 m/z. Cyclomethicone N5 was used as the lock mass with an m/z value of 371.10123. The MS1 full scan was followed by a maximum of five data-dependent MS2 fragmentation spectra of the most abundant ion species. The dynamic exclusion duration of the previously measured analytes was set at 30 s. The collision energy for collision-induced dissociation (CID) was set to 35 eV. The samples were measured in a randomized order to reduce bias from measurement conditions. All samples for LC-MS measurement contained the internal standard ampicillin sodium salt (BioScience grade, Carl Roth GmbH + Co. KG, Country) at a concentration of 1×10^−5^ mol/L.

**Data processing and annotation of metabolites**

For untargeted LC-MS analysis, Xcalibur (Thermo Fisher Scientific) RAW files were converted to mzML files using MSconvert (ProteoWizard) (Chambers et al., 2012), followed by feature extraction using MZmine 4 (Pluskal et al., 2010; 43. Heuckeroth et al., 2024) using the following parameters: for mass detection, the noise level was 1e-04 for MS1 and 0 for MS2. For chromatogram building, the mass tolerance was set as 0.002 m/z or 10 ppm, the minimum number of consecutive scans as 4, and the minimum height as 5E4. Chromatograms were smoothed with the Savitzky Golay algorithm, followed by a local minimum search for chromatographic deconvolution with a minimum search range of 0.1 min, minimum ratio of peak top to side of 1.8, and maximum peak duration of 3 min. For MS/MS scan pairing, the absolute tolerance of the MS1 to MS2 precursor was 0.05 Da. The peaks were de-isotope within 3 ppm m/z and 0.08 min retention time tolerances, aligned with 0.0015 m/z or 5 ppm mass tolerance and 0.5 min retention time tolerance, then gap-filled with 5 ppm m/z tolerance and 0.4 min retention time tolerance. The feature list was filtered by removing duplicates with 3 ppm mass tolerance and 0.1 min retention time tolerance. The feature list was exported as a feature quantification table (.csv). The feature quantification table was subsequently pre-processed using R scripts.

The features were tentatively annotated using different strategies: i) Features with MS2 spectra were searched against spectral libraries using the NIST MS Search program. The spectral libraries employed were the MSP format public databases from MassBank (Europe and North America), RIKEN, and GNPS platforms (Horai et al., 2010; Sawada et al., 2012), converted to NIST format using the NIST library conversion tool LIB2NIST Converter version 1.0.4.19. The minimum cosine similarity score (dot product) was employed to evaluate the annotation: 0.05 Da precursor and fragment ion mass tolerance and library search with a cosine score threshold of 0.7 and a minimum of four matched peaks. ii) Previous step-non-annotated features were searched by KEGG API using the function ‘keggFind’ in the KEGGREST R package. The database was searched using the estimated compound mass [M] (considering all our features as [M+H]+) within the default 5-ppm mass error. The obtained candidates were manually revised for their current KEGG REACTION and COMPOUND downloaded using the function get.kegg.all in the KEGGREST R package. iii) The tentative annotated features with MS2 were checked by manually screening the raw files for candidate metabolites using exact mass and comparing the MS2 fragmentation spectra with in-house reference library data. Some features were tentatively annotated using fragmentation pattern analysis (with an in-house database and public database). To confirm the results of manual annotation, standard compounds were measured as a reference for RT, exact mass, and MS2 fragmentation spectra, if available. iv) All annotated features were evaluated based on their chemical information, such as molecular formula, ontology, INCHIKEY, and SMILES. The MS1, MS2, and reference spectra of annotated metabolites are summarized in Table S2a. and MSP files (data excluded). Manual annotation of the fragmentation spectra of the m/z features is provided in Supporting Document 2. The level of identification was determined for each LC-MS m/z feature according to the Metabolomics Standard Initiative (Sunner et al., 2007) and is listed in main Table S2a.

**Deep Learning (DL) method**

We developed a deep learning-based pipeline that integrates Deep Canonical Correlation Analysis (DCCA) with a Genetic Algorithm (GA) to identify key metabolites driving BNI activity from high-dimensional metabolomic data. This method leverages the global search capability of GA and the representation power of deep neural networks for interpretable feature selection.

**1. Data Preprocessing**

Raw metabolomic data were normalized to the range [−1, 1] using Min-Max scaling to eliminate the effect of scale differences. The dataset was then divided into metabolite matrix X and a target vector Y representing BNI activity and converted into tensors for model input.

**2. Model Structure**

A Multi-Layer Perceptron (MLP) serves as the fitness function in the GA. It includes an input layer, several hidden layers with ReLU activations, and an output layer that predicts BNI activity.

**3. Custom Weighted Loss Function**

To enhance the model's sensitivity to important features, we designed a custom Weighted Mean Squared Error loss function defined as:$L(Y',Y,weights)=-\sum\lambda i$, where $\lambda i$ represents the feature correlation calculated via the covariance matrix. The loss function incorporates a regularization term to prevent numerical instability in the covariance matrix. Furthermore, the sample weights are dynamically adjusted based on the residuals between the predicted and target values, enabling the model to focus more on samples with smaller errors.

**4. Genetic Algorithm Optimization**

The GA identifies informative metabolite subsets by simulating an evolutionary search process. The population of the GA consists of 140 triplets of metabolites, where each triplet is encoded as a vector, evaluated using a deep neural network (MLP) to predict BNI activity, and assigned a fitness score based on prediction accuracy, as shown in Fig. 1. Individuals with higher fitness are more likely to be propagated into the GA’s next generation. Through repeated cycles of selection, crossover (recombining features), and mutation (random alteration), the GA explores a diverse range of features. Over multiple generations, the algorithm converges toward metabolite groups that consistently yield strong predictive performance, enabling robust and interpretable feature selection.


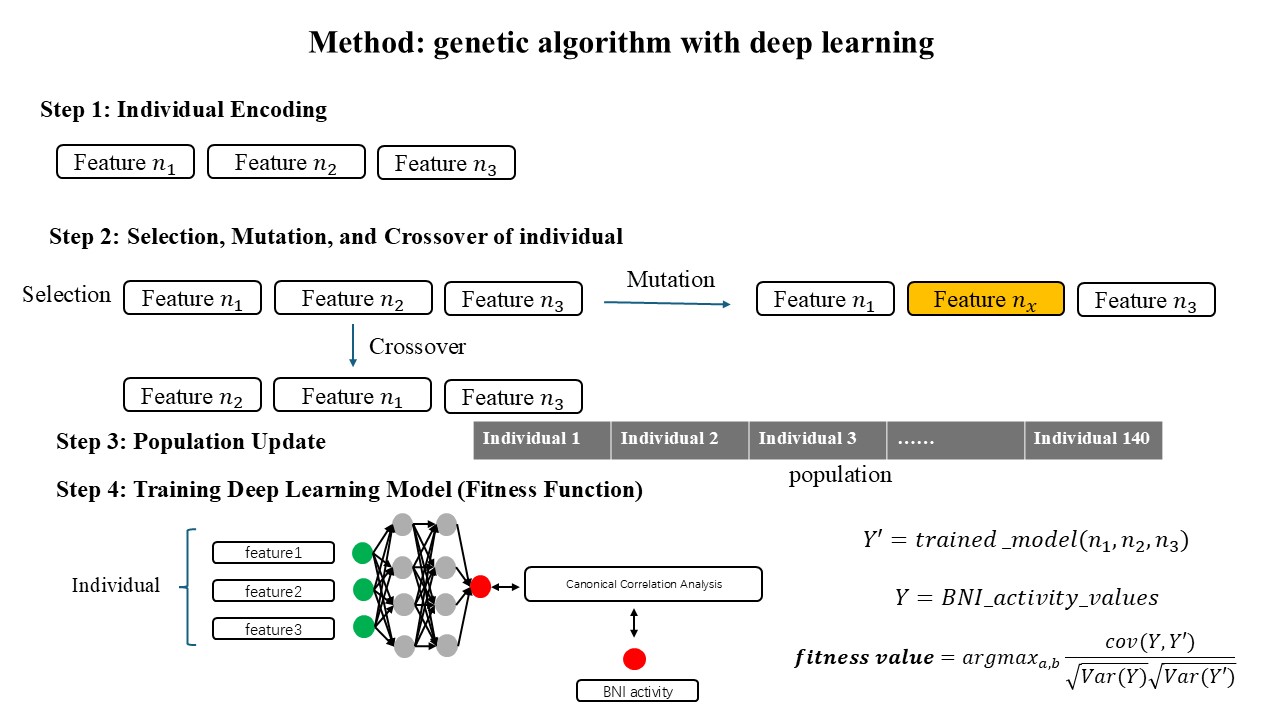


**5. Multi-Round Iterative Optimization**

To ensure robustness, the GA is run independently for multiple rounds, with each round generating an optimal feature group and its fitness value. The results from all rounds are aggregated, and the most frequently selected features are determined as the final feature set.

**6. Result Output and Cross-Validation**

Feature subsets and fitness values are saved per round for downstream analysis. To evaluate model stability, we performed 5 repetitions of cross-validation, where in each run, 80% of the samples were randomly selected without replacement, and the DCCA-GA algorithm was repeated independently. The consistency of highly ranked feature subsets is used to assess robustness.

**Supplementary Results**

## **Fast – track system validation and application to wheat root exudates BNI screening**

Cell concentration procedures significantly increased the cell densities and activity rates of AOM strains compared to routine cultures (Table 3, Figures 1-3, 5-6). The concentrated cultures exhibited stable cell densities and consistently higher activity rates across all five AOM strains, surpassing the maximum specific growth rates of the routine AOB cultures (Figure 4 and Table 4). When applied in different dose-schemes, the majority of the pure SNIs and BNIs yielded prevalent dose-response patterns for both the AOB (Figures 7A and 9-14) and the AOA (Figures 7B and 15-20). Ιnhibition thresholds revealed differences in efficacy and strain sensitivity, consistent with literature (Table 5 and Figures 21-23). Specifically, nitrapyrin was the most potent NI against AOB, also active towards AOA, while ethoxyquin was the most effective against AOA (Figure 8). DMPP, MHPP and 1,9-decanediol primarily targeted AOB, whereas the efficacy of sakuranetin was more strain- than domain-dependent (Figure 8). *N. ureae* was the most sensitive AOM strain against four of the six NIs tested (nitrapyrin, sakuranetin, 1,9-decanediol, and MHPP) (Figure 8). Comparison of these inhibition thresholds to literature showed complete data congruency for *N. multiformis*, with the sole exception of ethoxyquin, while larger variations were observed for the AOA, specifically for the less potent AOA NIs, MHPP and 1,9-decanediol (Table 5, Figures 21-23).

This strain-specific response remained consistent when screening the ammonia oxidation inhibition by wheat root exudates, with a wheat genotype-specific effect. Genotype g32 was the most inhibitory against three AOM reporter strains (main Figure 4A), but did not significantly inhibit *N. viennensis*, which was mainly inhibited by root exudates of g34 (main Figure 4A). Overall, “*Ca.* N. franklandianus” was the most sensitive strain to the REs tested, followed by AOB, with *N. viennensis* being the least sensitive AOM strain (main Figure 4B). AOB were slightly more sensitive compared to AOA (W-statistic = 27022, *p-value* < 0.05) (main Figure 4C), and REs from Austrian genotypes exhibited significantly higher potency than those from Indian genotypes (W-statistic = 36041, *p-value* *<* 0.001) (main Figure 4D). Hierarchical clustering revealed distinct inhibitory patterns grouping root exudates into several clusters (main Figure 4E). Notable clusters include Clusters A and B, where g32 and g9 were the most potent inhibitors and Cluster C, where g34 primarily inhibited AOA (main Figure 4E). Clusters D, H and I encompass REs of Austrian genotypes, with significant inhibitory activity on “*Ca.* N. franklandianus”, while Clusters J and K featured g36 and g28 with targeted inhibitory activity on *N. multiformis* (main Figure 4E). Clusters E and F consisted of root exudates with low inhibitory potential (main Figure 4E). Principal Component Analysis (PCA) reaffirmed g9, g32 and g34 as the most potent REs and distinctly separated root exudates targeting AOB (g9, g28, g36) or AOA (g34, g3, g10, g17), explaining 78.6% of the variation (Figure 24).

# **Tables**

**Table 1.** Summary of the provider and purity of each compound used in the study.

| Compound | Provider | Purity |
| --- | --- | --- |
| DMPP | Cayman Chemical (1180 East Ellsworth Road, Ann Arbor, Michigan, 48108, USA) | ≥ 98% |
| Nitrapyrin | Sigma – Aldrich (Chemie GmbH, Eschenstr. 5, 82024, Taufkirchen, Germany) | ≥ 98% |
| Ethoxyquin | Sigma – Aldrich (Chemie GmbH, Eschenstr. 5, 82024, Taufkirchen, Germany) | 96.9% |
| MHPP | BLDpharm (No.1 Wangdong Middle Road, Building 5, Songjiang District, Shanghai 201601, China) | 99.21% |
| Sakuranetin | Syngenta Crop Protection AG (Rosentalstr. 67, 4058 Basel, Switzerland) | > 95% |
| 1,9-decanediol | WuXi AppTec (288 Fute Zhong Road, Shanghai, 200131, China) | 95% |

Abbreviations: DMPP – 3,4-dimethylpyrazole (phosphate), MHPP - methyl 3-(4-hydroxyphenyl) propionate

**Table 2.** Overview of the concentrations of the nitrification inhibitors used for the validation of the fast-track testing system.

| *Nitrosospira multiformis* | | | | | | | |
| --- | --- | --- | --- | --- | --- | --- | --- |
|  | Compound | Tested concentrations (μM) | | | | | |
| SNIs | DMPP | 0.25 | 0.5 | 1 | 10 |  |  |
|  | Nitrapyrin | 0.25 | 0.5 | 1 | 5 |  |  |
|  | Ethoxyquin | 50 | 100 | 250 | 500 |  |  |
| BNIs | MHPP | 50 | 100 | 250 | 500 |  |  |
|  | Sakuranetin | 25 | 75 | 150 | 300 |  |  |
|  | 1,9-decanediol | 50 | 100 | 400 | 800 |  |  |
| *Nitrosomonas ureae* | | | | | | | |
|  | Compound | Tested concentrations (μM) | | | | | |
| SNIs | DMPP | 0.25 | 0.5 | 1 | 10 |  |  |
|  | Nitrapyrin | 0.1 | 0.25 | 0.5 | 5 |  |  |
|  | Ethoxyquin | 5 | 25 | 50 | 100 | 150 | 250 |
| BNIs | MHPP | 25 | 50 | 100 | 250 |  |  |
|  | Sakuranetin | 1 | 5 | 25 | 75 |  |  |
|  | 1,9-decanediol | 50 | 100 | 400 | 800 |  |  |
| *Nitrosomonas communis* | | | | | | | |
|  | Compound | Tested concentrations (μM) | | | | | |
| SNIs | DMPP | 0.5 | 1 | 2 | 4 |  |  |
|  | Nitrapyrin | 0.5 | 1 | 2 | 4 |  |  |
|  | Ethoxyquin | 50 | 150 | 300 | 450 | 600 |  |
| BNIs | MHPP | 100 | 250 | 350 | 500 |  |  |
|  | Sakuranetin | 25 | 50 | 150 | 300 | 600 |  |
|  | 1,9-decanediol | 400 | 600 | 800 | 1000 |  |  |
| “*Candidatus* Nitrosocosmicus franklandianus” | | | | | | | |
|  | Compound | Tested concentrations (μM) | | | | | |
| SNIs | DMPP | 500 | 1000 | 2000 | 3000 |  |  |
|  | Nitrapyrin | 5 | 10 | 20 | 40 |  |  |
|  | Ethoxyquin | 1.2 | 2.5 | 5 | 10 |  |  |
| BNIs | MHPP | 500 | 1000 | 2000 | 4000 |  |  |
|  | Sakuranetin | 10 | 20 | 40 | 80 |  |  |
|  | 1,9-decanediol | 500 | 1000 | 2000 | 4000 |  |  |
| *Nitrososphaera viennensis* | | | | | | | |
|  | Compound | Tested concentrations (μM) | | | | | |
| SNIs | DMPP | 500 | 1000 | 1500 | 2000 |  |  |
|  | Nitrapyrin | 10 | 20 | 40 | 80 |  |  |
|  | Ethoxyquin | 1.2 | 2.5 | 5 | 10 |  |  |
| BNIs | MHPP | 250 | 500 | 1000 | 2000 |  |  |
|  | Sakuranetin | 10 | 20 | 40 | 80 |  |  |
|  | 1,9-decanediol | 250 | 500 | 1000 | 2000 |  |  |

Abbreviations: SNIs – Synthetic Nitrification Inhibitors, BNIs – Biological Nitrification Inhibitors, DMPP – 3,4-dimethylpyrazole (phosphate), MHPP - methyl 3-(4-hydroxyphenyl) propionate

**Table 3.** Overview of cell densities at different points of the AOM routine cultures and harvesting protocol. For AOB, data are available for two extra steps referring to the cell harvesting procedure. Values represent the average cells mL⁻¹ of three consecutive tests (± standard deviation) (SD). Values designated by different lowercase letters belong to groups statistically different at the 5% level, as determined by the Kruskal-Wallis test. “Routine culture initial” corresponds to the starting point of the routine cultures. “Routine culture final” corresponds to the late logarithmic growth phase of the routine cultures, just before AOB cell harvesting. The term “Inocula after cell harvesting” refers to the “After cell harvesting” step in Figures 1-3.

| *Nitrosospira multiformis* | |
| --- | --- |
| Step | Cell density (cells mL^-1^) (± 1SD) |
| Routine culture initial | 2.86×10^5^ (± 1.45×10^5^) d |
| Routine culture final | 3.02×10^7^ (± 9.55×10^6^) c |
| Inocula after cell harvesting | 1.69×10^8^ (± 7.09×10^7^) b |
| Merged inoculum | 4.35×10^8^ (± 4.17×10^7^) a |
| Concentrated culture initial | 3.57×10^7^ (± 2.10×10^6^) c |
| *Nitrosomonas ureae* | |
| Step | Cell density (cells mL^-1^) (± 1SD) |
| Routine culture initial | 3.62×10^5^ (± 1.76×10^5^) e |
| Routine culture final | 3.95×10^7^ (± 4.41×10^6^) d |
| Inocula after cell harvesting | 5.12×10^8^ (± 1.15×10^8^) b |
| Merged inoculum | 1.62×10^8^ (± 1.75×10^8^) a |
| Concentrated culture initial | 9.43×10^7^ (± 1.16×10^7^) c |
| *Nitrosomonas communis* | |
| Step | Cell density (cells mL^-1^) (± 1SD) |
| Routine culture initial | 1.14×10^3^ (± 6.02×10^2^) d |
| Routine culture final | 3.96×10^4^ (± 2.35×10^4^) c |
| Inocula after cell harvesting | 1.36×10^6^ (± 4.46×10^5^) a |
| Merged inoculum | 2.79×10^6^ (± 1.24×10^6^) a |
| Concentrated culture initial | 1.54×10^5^ (± 8.79×10^4^) b |
| “*Candidatus* Nitrosocosmicus franklandianus” | |
| Step | Cell density (cells mL^-1^) (± 1SD) |
| Routine culture initial | 5.15×10^4^ (± 6.20×10^3^) c |
| Routine culture final | 5.15×10^6^ (± 5.07×10^5^) b |
| Concentrated culture initial | 1.28×10^7^ (± 1.79×10^6^) a |
| *Nitrososphaera viennensis* | |
| Step | Cell concentration (cells mL^-1^) (± 1SD) |
| Routine culture initial | 7.20×10^3^ (± 5.27×10^2^) c |
| Routine culture final | 1.44×10^7^ (± 1.05×10^6^) b |
| Concentrated culture initial | 8.70×10^7^ (± 1.12×10^5^) a |

**Table 4.** Overview of AOB specific growth rates and concentrated cultures activity rates. μ_max_ and activity rates are presented (± standard error). R^2^ values refer to the linear regressions utilized for μ_max_ and activity rates (shown in Figure 4).

| *Nitrosospira multiformis* | | Reference |
| --- | --- | --- |
| Routine culture specific growth rate | 0.045 ± 0.002 h^-1^ (R^2^ = 0.84) | This study |
| Literature specific growth rate | 0.050 ± 0.004 h^-1^ | (29) |
|  | 0.036 | (38) |
| Concentrated cultures activity rate | 0.113 ± 0.004 (R^2^ = 0.90) | This study |
| *Nitrosomonas ureae* | |  |
| Routine culture specific growth rate | 0.036 ± 0.001 h^-1^ (R^2^ = 0.96) | This study |
| Concentrated cultures activity rate | 0.160 ± 0.007 (R^2^ = 0.84) | This study |
| *Nitrosomonas communis* | |  |
| Routine culture specific growth rate | 0.031 ± 0.001 h^-1^ (R^2^ = 0.85) | This study |
| Concentrated cultures activity rate | 0.132 ± 0.002 (R^2^ = 0.98) | This study |

**Table 5.** Comparison of the SNI and BNI inhibition thresholds obtained from the fast-track bioassay and the inhibition thresholds from liquid batch culture assays in literature. Mean EC_50_ values are presented accompanied by the standard error (SE). References for the literature inhibition thresholds are provided, alongside the statistical similarity of the mean values at a 95% confidence interval, with a “Comparable” label indicating values that are close enough to be comparable but not statistically similar. Visualisation of the comparison at a 95% confidence interval can be found in Supplementary Figures 21-23.

| *Nitrosospira multiformis* | | | | |
| --- | --- | --- | --- | --- |
| Nitrification Inhibitor | **Fast-track bioassay EC50 ± 1SE (μM)** | **Literature EC50 ± 1SE (μM)** | **Reference** | **Statistical similarity (α = 0.05)** |
| Nitrapyrin | 0.45 ± 0.03 | 0.8 ± 0.3 | (7) | YES |
| DMPP | 0.48 ± 0.05 | 0.6 ± 0.1 | (7) | YES |
| Ethoxyquin | > 500 | 214.8 ± 39.6 | (7) | NO |
| MHPP | 102.8 ± 6.41 | 104 ± 12.8 | (11) | YES |
| Sakuranetin | 120.3 ± 17.42 | 144.3 ± 19 | (11) | YES |
| 1,9-decanediol | 467.3 ± 18.83 | 428.1 ± 2.47 | (29) | YES |
| *“Candidatus* Nitrosocosmicus franklandianus*”* | | | | |
| Nitrification Inhibitor | **Fast-track bioassay EC50 ± 1SE (μM)** | **Literature EC50 ± 1SE (μM)** | **Reference** | **Statistical similarity (α = 0.05)** |
| Nitrapyrin | 33.6 ± 4.1 | 1.0 ± 0.3 | (7) | NO |
| DMPP | 1890 ± 278 | 1774 ± 440 | (7) | YES |
| Ethoxyquin | 6.37 ± 0.62 | 1.4 ± 0.3 | (7) | Comparable |
| MHPP | 2312 ± 280 | 877.7 ± 165.3 | (11) | NO |
| Sakuranetin | 44.9 ± 1.20 | 61 ± 10 | (29) | YES |
| 1,9-decanediol | > 4000 | 212 ± 0.9 | (29) | NO |
| *Nitrososphaera viennensis* | | | | |
| Nitrification Inhibitor | **Fast-track bioassay EC50 ± 1SE (μM)** | **Literature EC50 ± 1SE (μM)** | **Reference** | **Statistical similarity (α = 0.05)** |
| Nitrapyrin | **>** 80 | 118 ± 20 | (39) | Comparable |
| DMPP | 1847 ± 87 | *NO DATA* |  |  |
| Ethoxyquin | 2.18 ± 0.06 | *NO DATA* |  |  |
| MHPP | > 2000 | 647 ± 46 | (29) | NO |
| Sakuranetin | 17.5 ± 2 | 88 ± 7 | (29) | Comparable |
| 1,9-decanediol | > 2000 | 418 ± 19 | (29) | NO |

# **Figures**

*
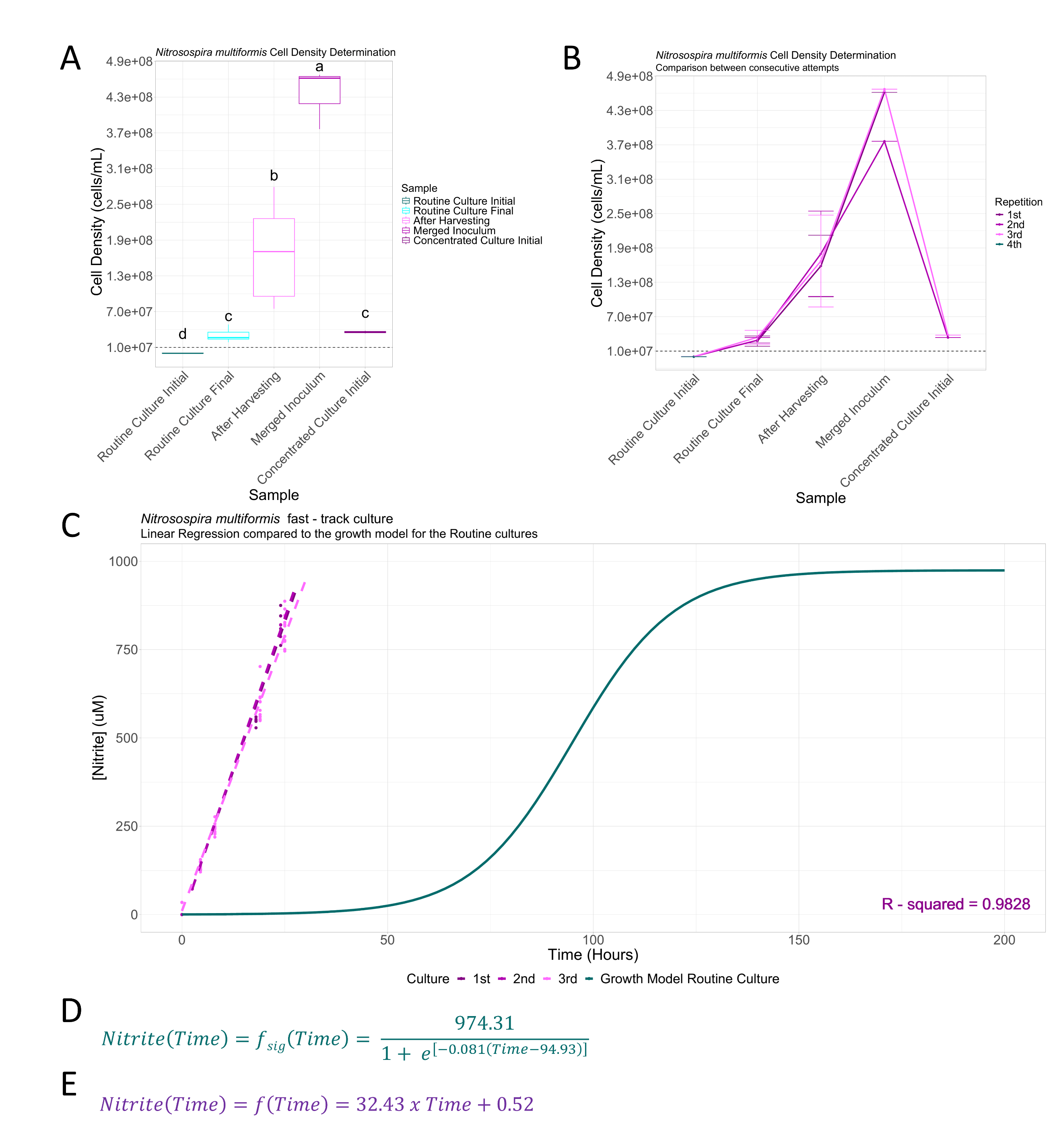
*

***Figure 1.*** ***Nitrosospira multiformis fast – track assay protocol standardisation. (A) Cell density across different protocol steps.*** *Box plot of pooled qPCR results for the different points of the cell harvesting procedure. The y – axis values correspond to cell density, while the five different points of the procedure are plotted on the x – axis. The black dashed line represents the limit below which the box plot is not to scale. Lowercase letters above the boxes indicate the grouping according to the Kruskal – Wallis test.* ***(B)*** ***Cell density across different protocol repetitions.*** *Line plots of qPCR results between consecutive tests of the cell harvesting procedure. Different tests are represented by different colours according to the legend. The coloured error bars represent the range between -1 and +1 standard deviation from the mean value. The black dashed line indicates the limit below which the line plot is not to scale.* ***(C) Activity comparison between routine and fast-track cultures.*** *Activity curve of N. multiformis fast – track culture (magenta lines) compared to the activity model curve of the respective routine cultures (blue line). The points represent individual nitrite values from the biological replicates at each time-point of each fast – track culture. The R^2^ value refers to the linear regression of the pooled fast – track culture data.*

*
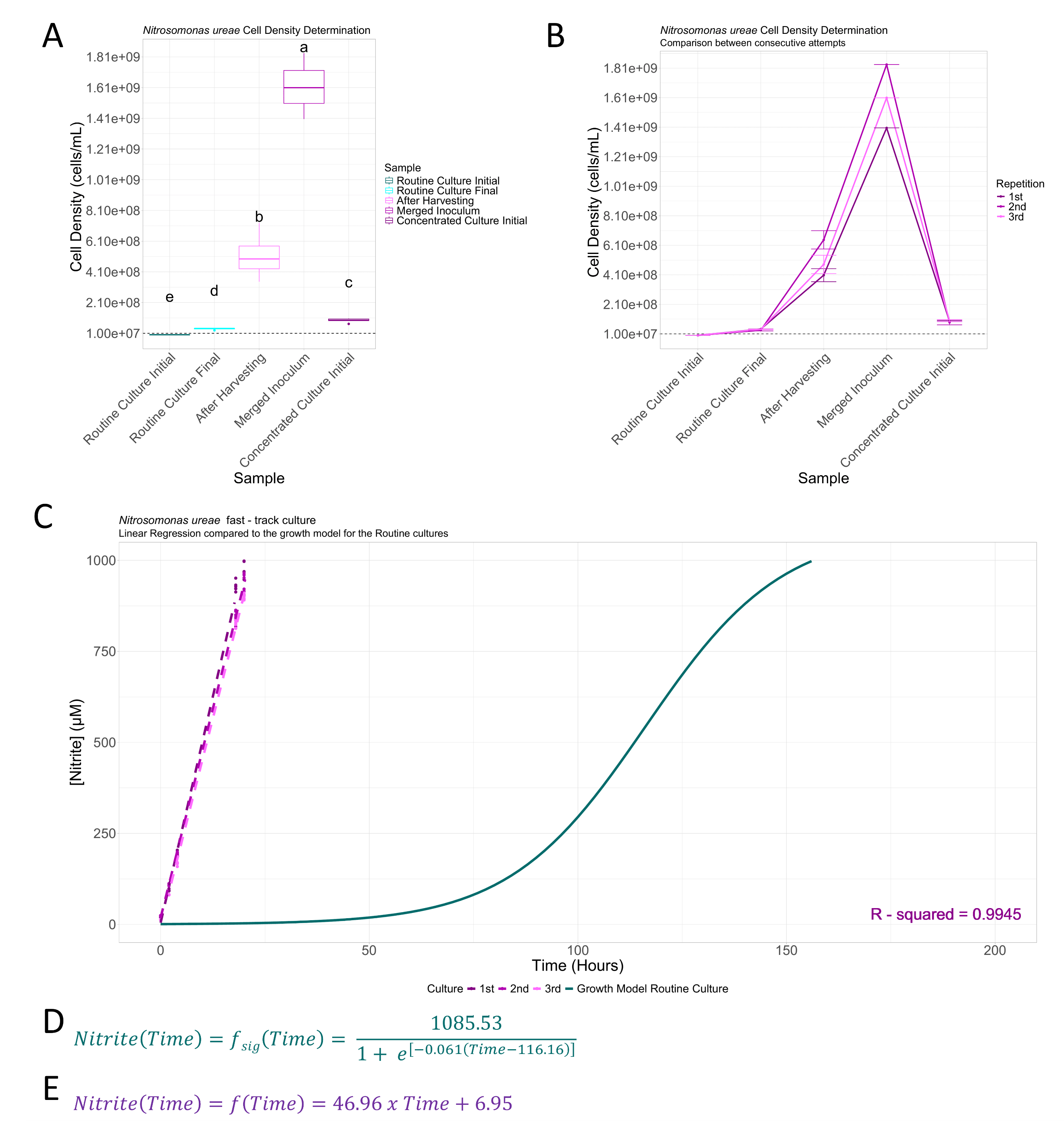
*

***Figure 2.*** ***Nitrosomonas ureae fast – track assay protocol standardisation. (A) Cell density across different protocol steps.*** *Box plot of pooled qPCR results for the different points of the cell harvesting procedure. The y – axis values correspond to cell density, while the five different points of the procedure are plotted on the x – axis. The black dashed line represents the limit below which the box plot is not to scale. Lowercase letters above the boxes indicate the grouping according to the Kruskal – Wallis test.* ***(B)*** ***Cell density across different protocol repetitions.*** *Line plots of qPCR results between consecutive tests of the cell harvesting procedure. Different tests are represented by different colours according to the legend. The coloured error bars represent the range between -1 and +1 standard deviation from the mean value. The black dashed line indicates the limit below which the line plot is not to scale.* ***(C) Activity comparison between routine and fast-track cultures.*** *Activity curve of N. ureae fast – track culture (magenta lines) compared to the activity model curve of the respective routine cultures (blue line). The points represent individual nitrite values from the biological replicates at each time-point of each fast – track culture. The R^2^ value refers to the linear regression of the pooled fast – track culture data.*

*
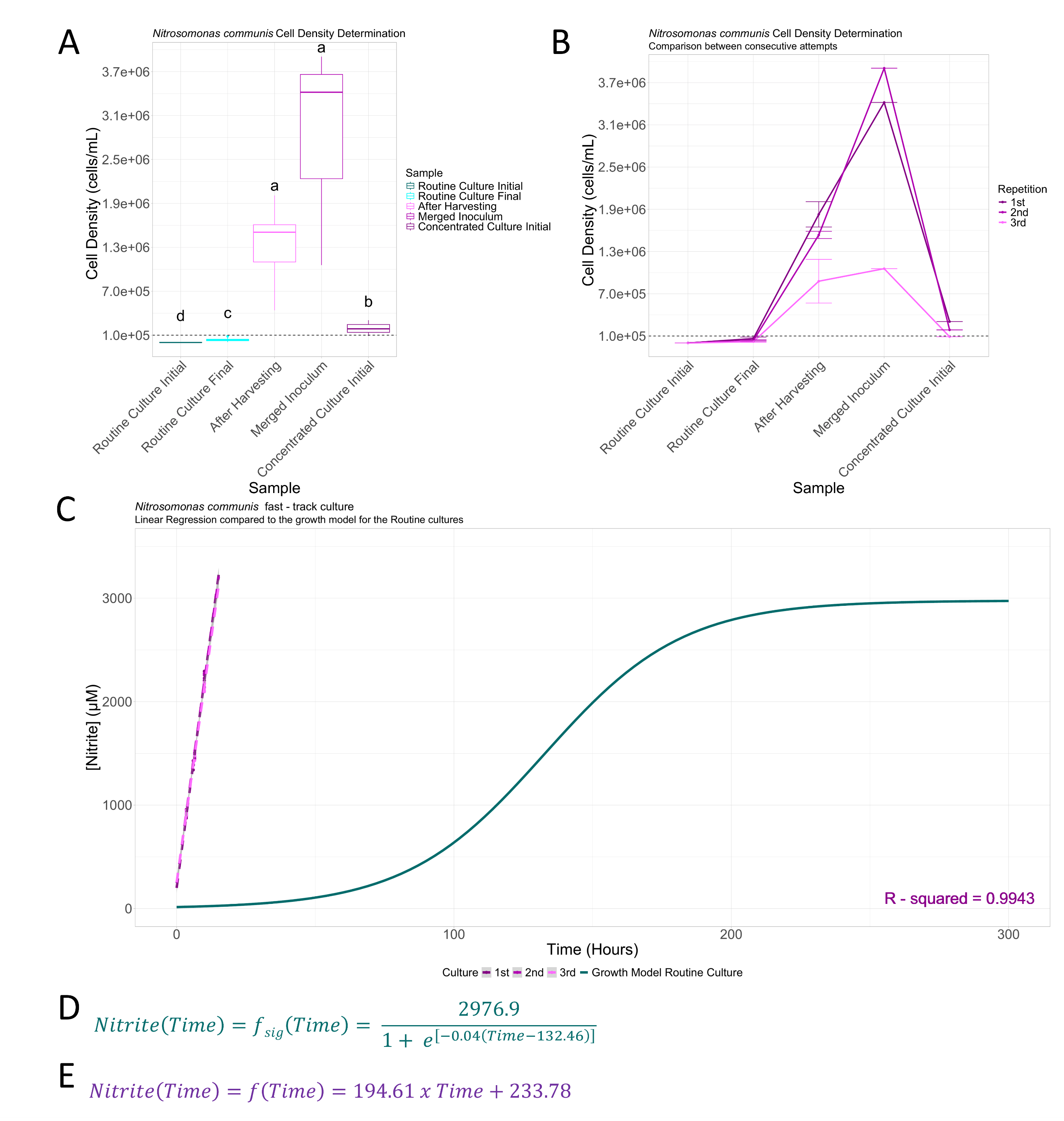
*

***Figure 3.*** ***Nitrosomonas communis fast – track assay protocol standardisation. (A) Cell density across different protocol steps.*** *Box plot of pooled qPCR results for the different points of the cell harvesting procedure. The y – axis values correspond to cell density, while the five different points of the procedure are plotted on the x – axis. The black dashed line represents the limit below which the box plot is not to scale. Lowercase letters above the boxes indicate the grouping according to the Kruskal – Wallis test.* ***(B)*** ***Cell density across different protocol repetitions.*** *Line plots of qPCR results between consecutive tests of the cell harvesting procedure. Different tests are represented by different colours according to the legend. The coloured error bars represent the range between -1 and +1 standard deviation from the mean value. The black dashed line indicates the limit below which the line plot is not to scale.* ***(C) Activity comparison between routine and fast-track cultures.*** *Activity curve of N. communis fast – track culture (magenta lines) compared to the activity model curve of the respective routine cultures (blue line). The points represent individual nitrite values from the biological replicates at each time-point of each fast – track culture. The R^2^ value refers to the linear regression of the pooled fast – track culture data.*

*
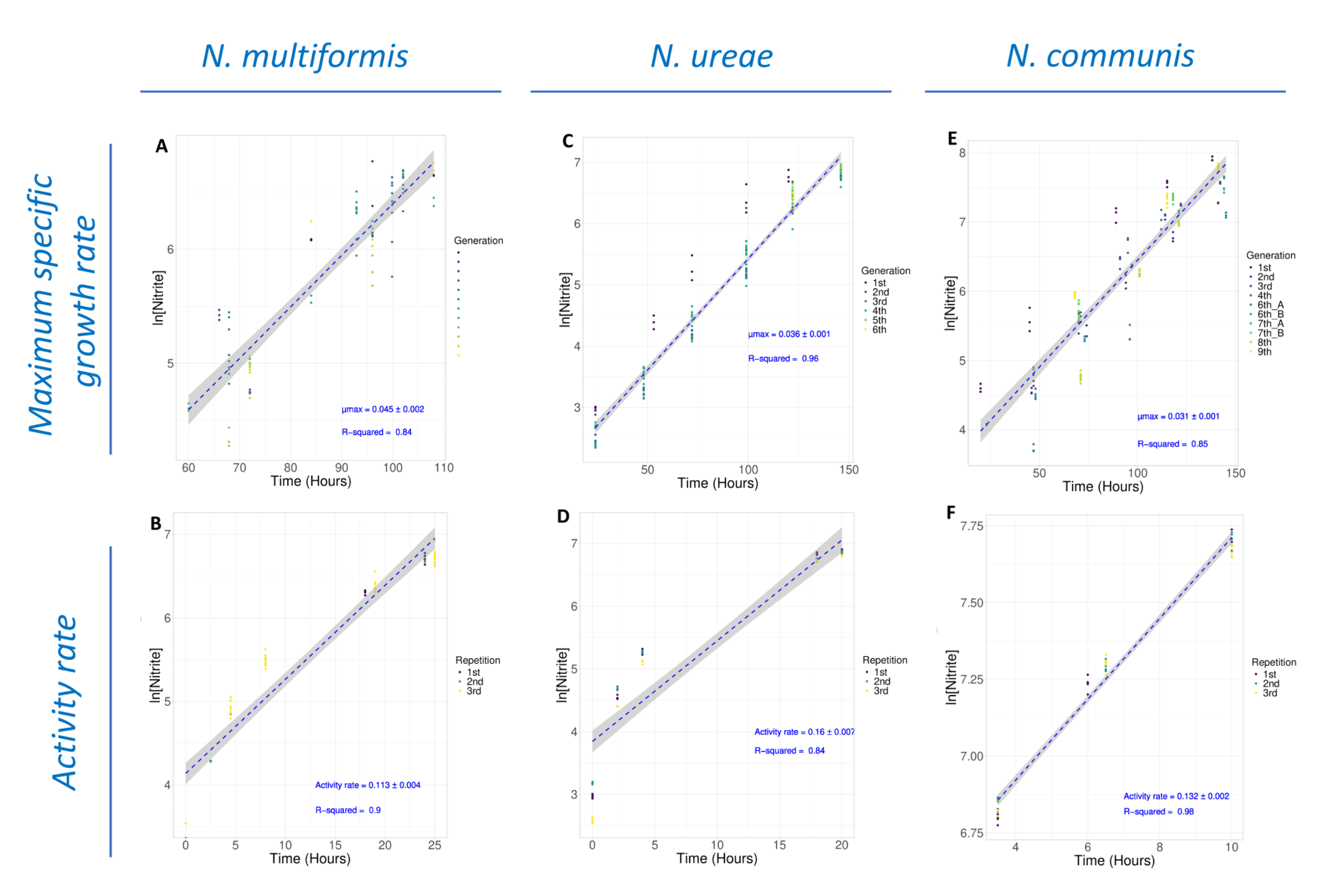
*

***Figure 4.*** ***Calculation of AOB maximum specific growth and activity rates.*** *Estimation of* ***(A,C,E)*** *the maximum specific growth rate (μ_max_) of AOB routine cultures and* ***(B, D, F)*** *the activity rate of the AOB concentrated cultures for N. multiformis* ***(A-B)****, N. ureae* ***(C-D)****, and N. communis* ***(E-F)****. Log-transformed (ln) nitrite production values are plotted against time for consecutive generations of the AOB routine cultures or consecutive repetitions of the fast – track assay. The dotted blue line represents the linear regression curve, with shaded areas indicating the standard error. Data points for each generation or repetition are denoted by different colour, as indicated in the legend. μ_max_ and activity rate values with the respective standard errors are presented on the right side of the graphs, accompanied by the adjusted R^2^ of the respective linear regression.*

*
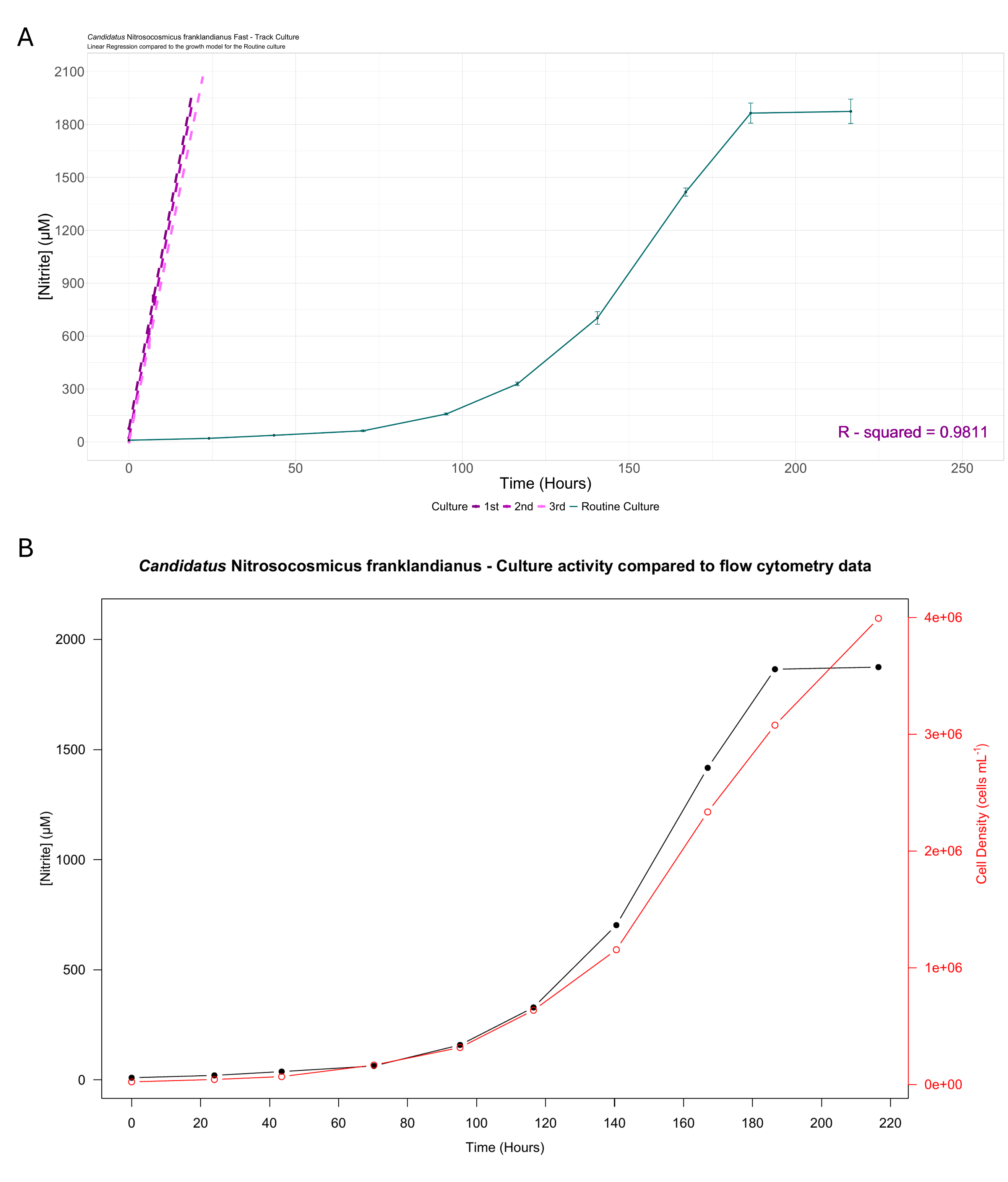
*

***Figure 5.*** ***“Ca.* Nitrosocosmicus franklandianus*” fast – track culture standardisation. (A) Activity comparison between routine and fast-track cultures.*** *Activity curves of “Ca.* N. franklandianus*” fast – track cultures (magenta lines) are compared to the activity model curve of the respective routine cultures (blue line). The points represent the individual nitrite values from biological replicates at each time-point of each fast – track culture. The R^2^ value refers to the linear regression of the pooled fast – track cultures data.* ***(B) Cellular activity and growth curve congruence.*** *Comparison of the cellular activity curve and the growth curve derived from flow cytometry. Points represent mean values and each y – axis, coloured differently, corresponds to the respective curve.*

*
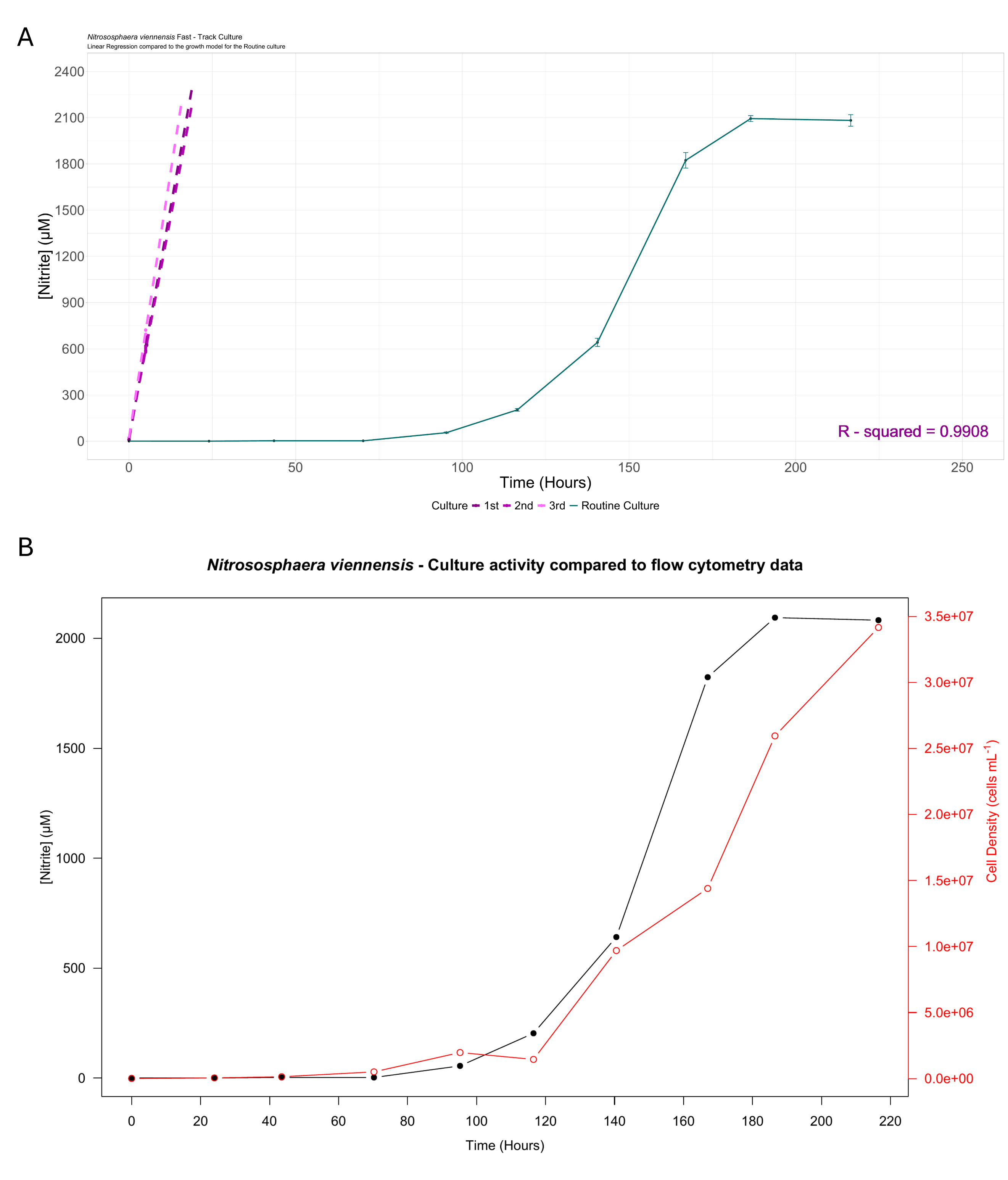
*

***Figure 6.*** ***Nitrososphaera viennensis fast – track culture standardisation. (A) Activity comparison between routine and fast-track cultures.*** *Activity curves of Nitrososphaera viennensis fast – track cultures (magenta lines) are compared to the activity model curve of the respective routine cultures (blue line). The points represent the individual nitrite values from biological replicates at each time-point of each fast – track culture. The R^2^ value refers to the linear regression of the pooled fast – track cultures data.* ***(B) Cellular activity and growth curve congruence.*** *Comparison of the cellular activity curve and the growth curve derived from flow cytometry. Points represent mean values and each y – axis, coloured differently, corresponds to the respective curve.*

*
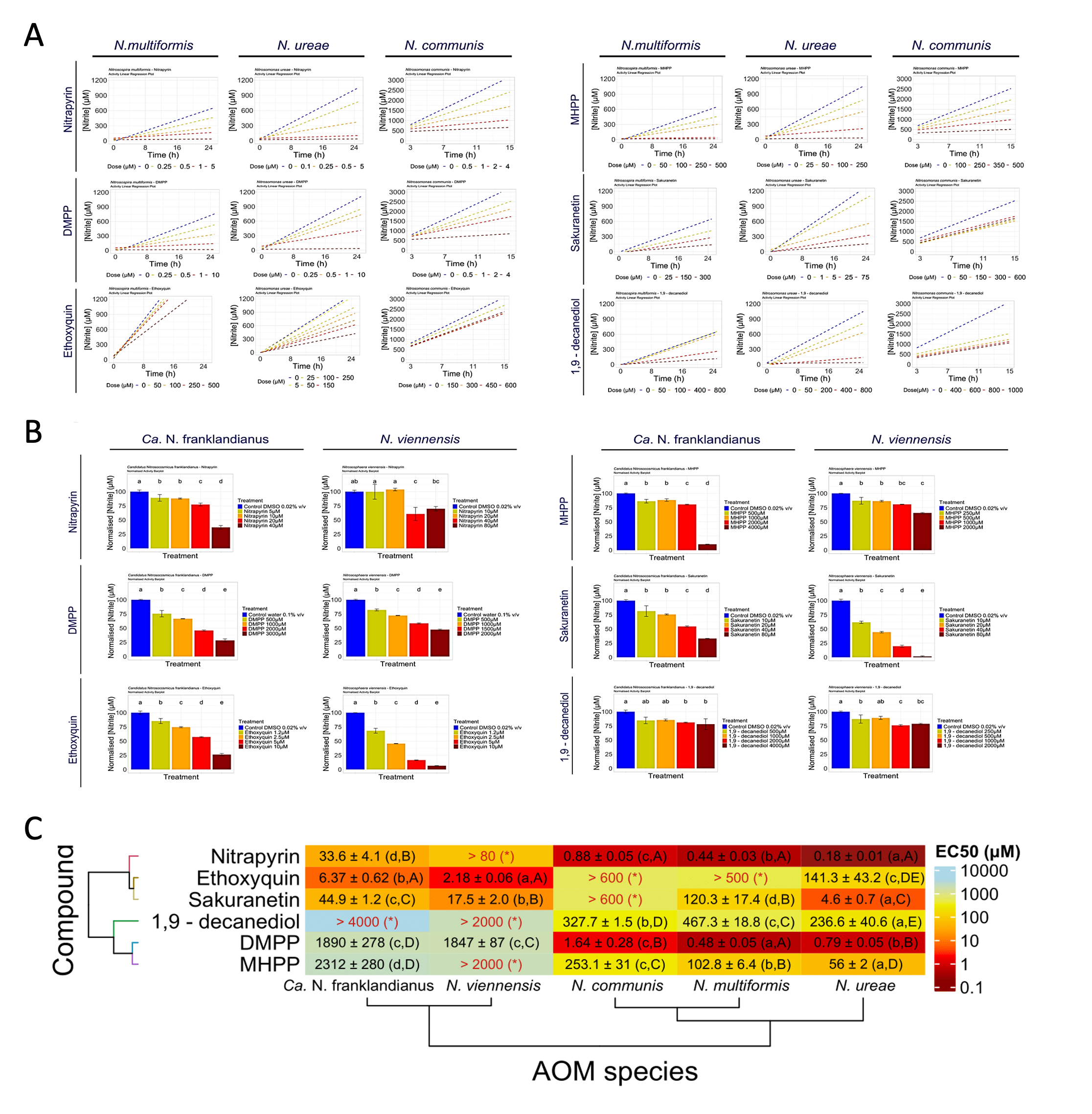
*

***Figure 7.*** ***Validation of the AOM fast-track assay with known SNIs and BNIs.*** ***(A-B)*** ***AOB and AOA strain responses to the tested inhibitors.*** *Each row represents one nitrification inhibitor (NI), with columns for individual AOM strains.* ***(A)*** *For AOB, the ammonia oxidation activity is shown via linear regression plots, comparing responses across dose rates and controls. Dashed lines indicate nitrite regression trends. Line colours correspond to different dose rates, as indicated in the legend on the right.* ***(B)*** *For AOA, the normalized ammonia oxidation activity data are presented in a bar plot, comparing responses across dose rates and controls. Bar colours correspond to different dose rates, as indicated in the legend on the right. Lowercase letters indicate Kruskal-Wallis groupings. EC_50_ modelling outputs are presented in Figures 9-20.*

*
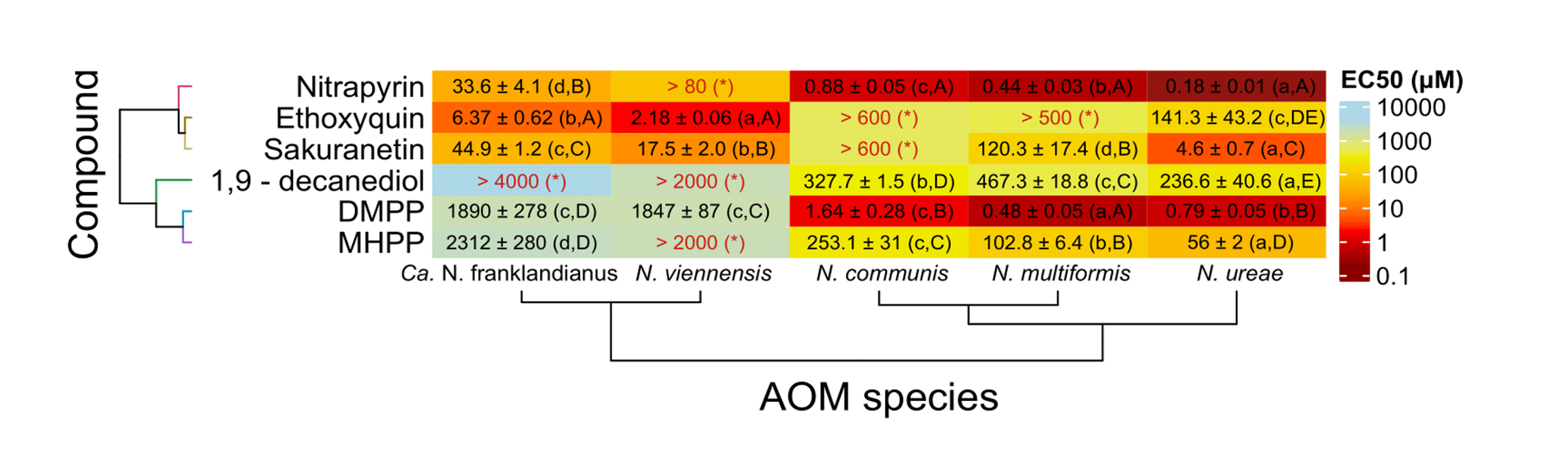
*

***Figure 8.*** ***EC_50_ values from fast-track assay with known SNIs and BNIs.*** *Heatmap of EC_50_ values for each NI across the five reporter AOM strains, with their respective standard errors. Box clours scale logarithmically by EC_50_ values, as indicted by the legend on the right. Red values indicate indefinite EC_50_ estimates. Lowercase letters in parentheses show groupings of EC_50_ values for each NI across strains, while uppercase letters indicate groupings across NIs per AOM strain. Asterisks (*) denote indefinite values, with box colours scaled based on the lowest limit value.*

*
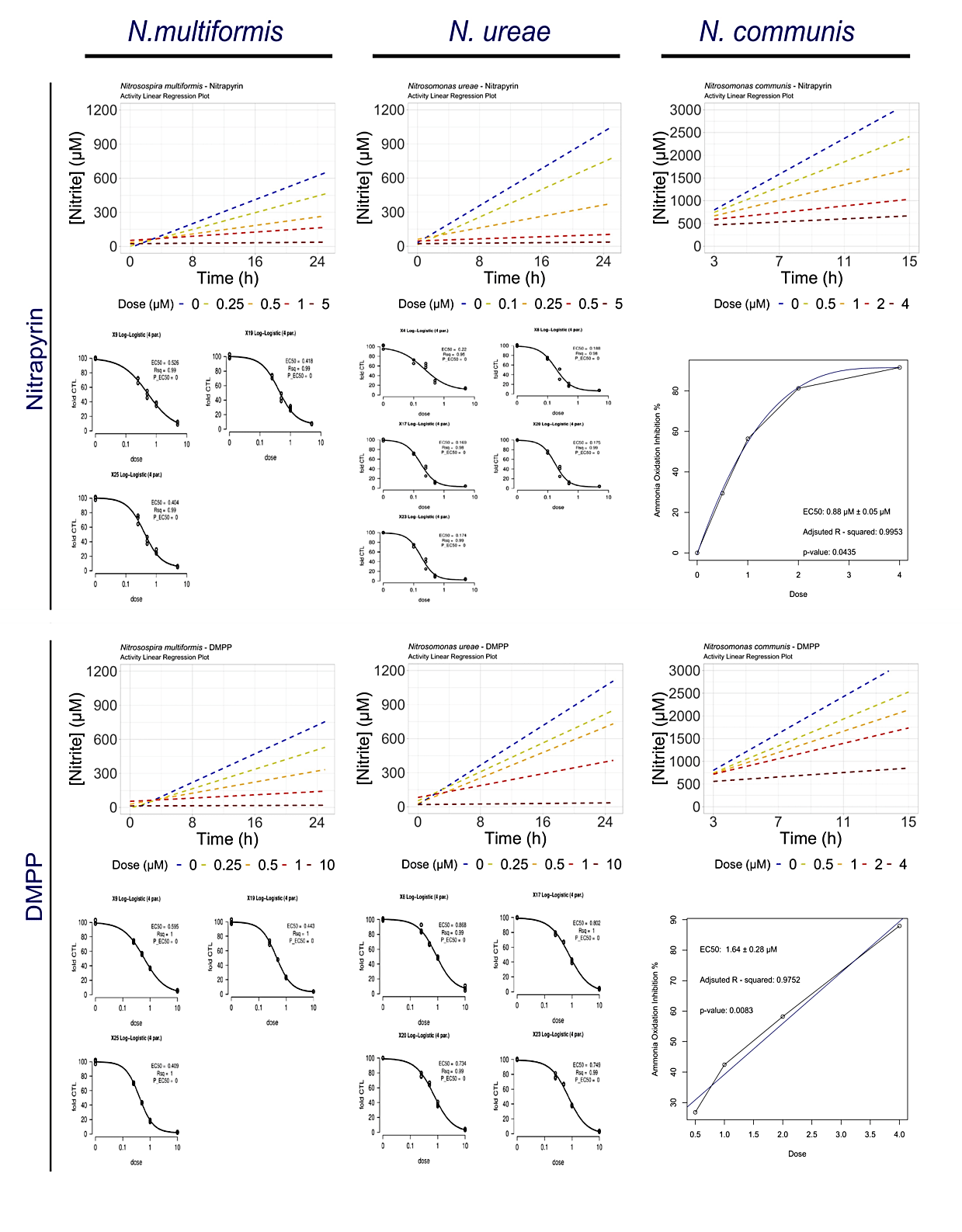
*

***Figure 9.*** ***Validation of the AOB fast – track assay with nitrapyrin.*** *The responses of the AOB strains to nitrapyrin are presented. The figure is divided into three columns, each representing one AOB strain. The ammonia oxidation activity is illustrated through a linear regression plot, comparing the AOB response to different dose rates, alongside the respective control treatment. The dashed lines in each plot represent the linear regression of nitrite values (data points not shown) at each time point for each treatment. Below the plot, a summary of the modeling of the AOB response, that led to the estimation of each EC_50_ value, is provided. For the log – logistic model, one model fit with an estimate of the EC_50_ is given for each time point. For polynomial or linear models, an aggregate model fit is presented with a final estimate of the EC_50_. Detailed information about the modeling procedure can be found in the Materials and Methods section.*

*
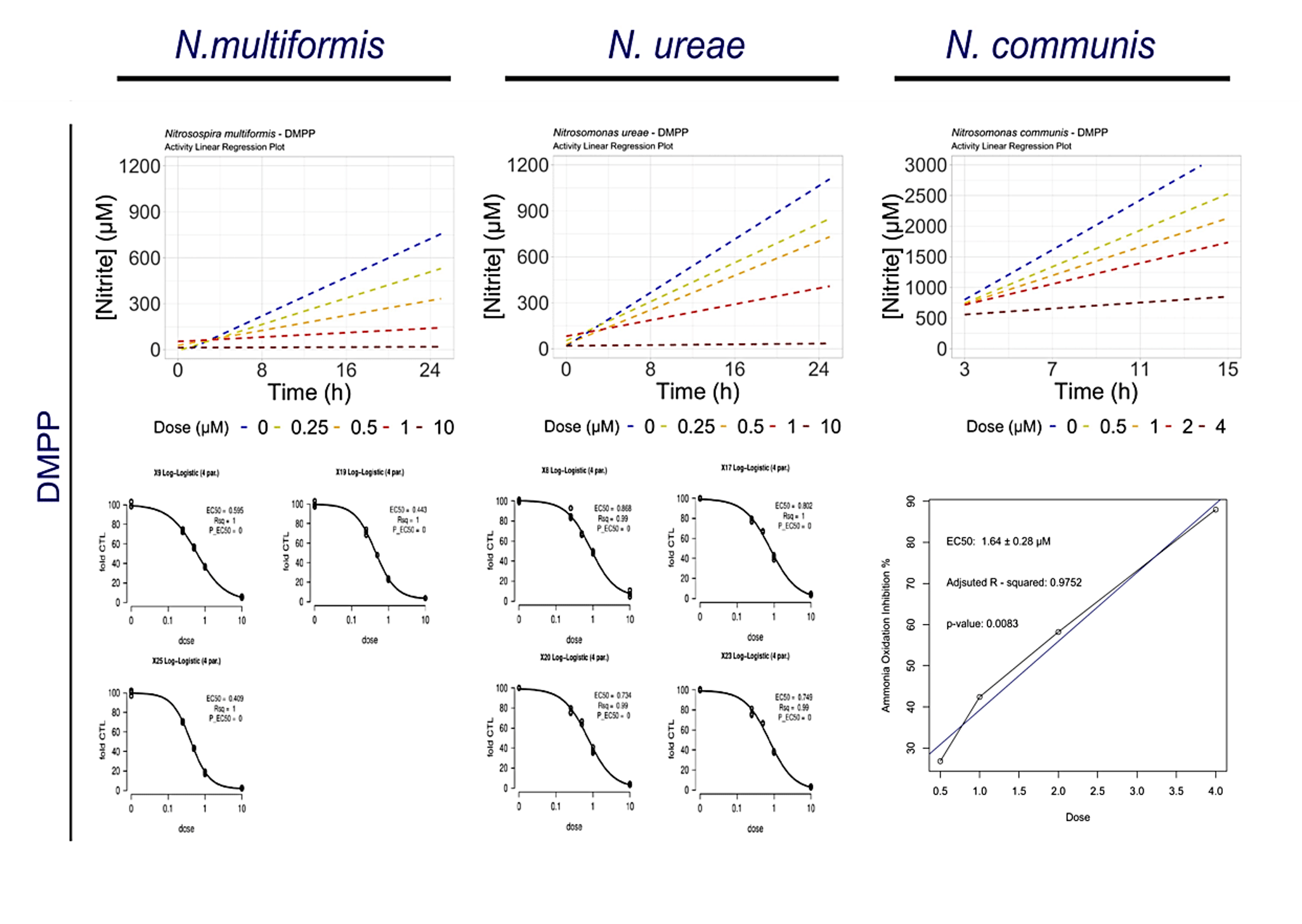
*

***Figure 10.*** ***Validation of the AOB fast – track assay with DMPP.*** *The responses of the AOB strains to DMPP are presented. The figure is divided into three columns, each representing one AOB strain. The ammonia oxidation activity is illustrated through a linear regression plot, comparing the AOB response to different dose rates, alongside the respective control treatment. The dashed lines in each plot represent the linear regression of nitrite values (data points not shown) at each time point for each treatment. Below the plot, a summary of the modeling of the AOB response, that led to the estimation of each EC_50_ value, is provided. For the log – logistic model, one model fit with an estimate of the EC_50_ is given for each time point. For polynomial or linear models, an aggregate model fit is presented with a final estimate of the EC_50_. Detailed information about the modeling procedure can be found in the Materials and Methods section.*

*
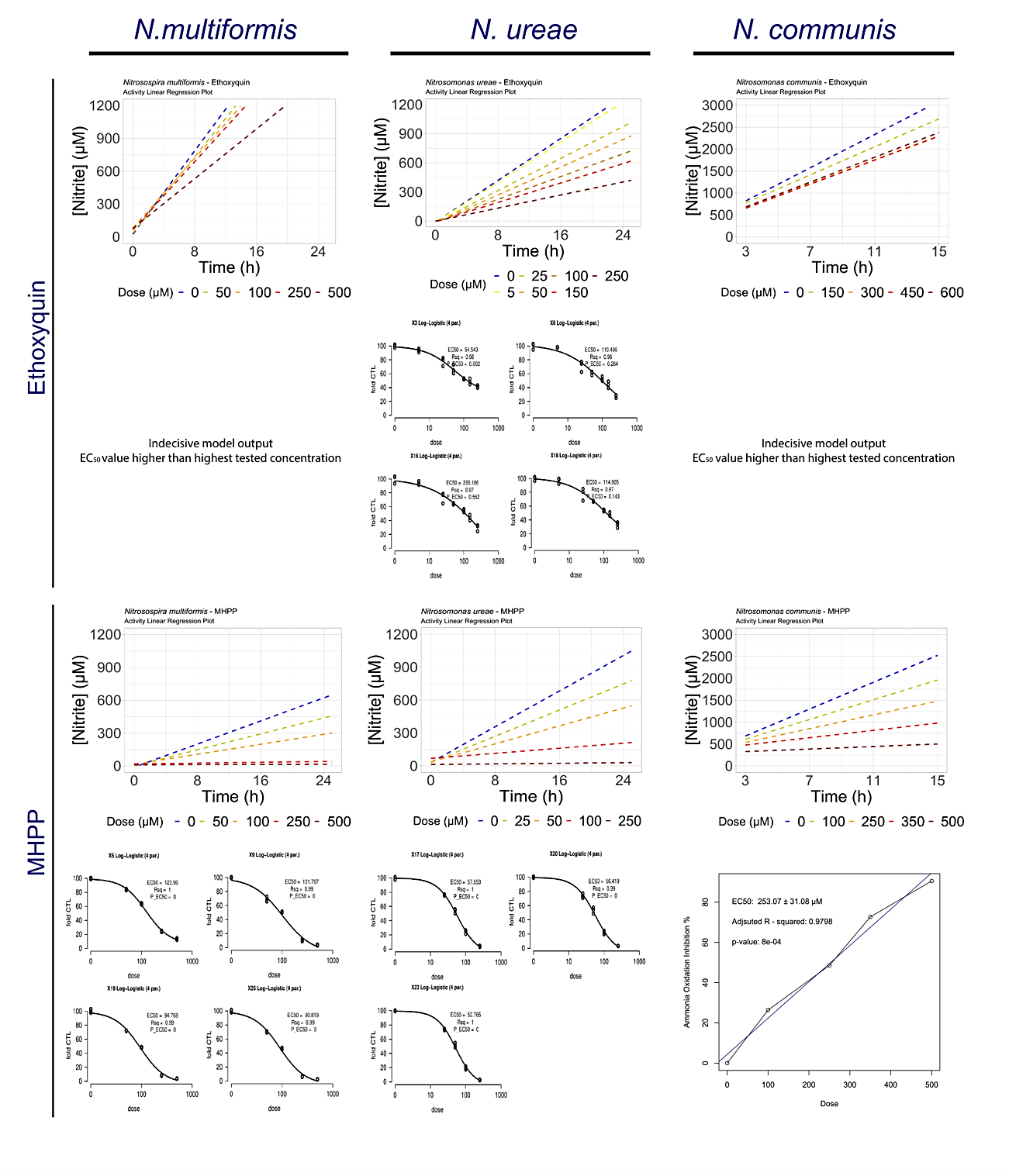
*

***Figure 11.*** ***Validation of the AOB fast – track assay with ethoxyquin.*** *The responses of the AOB strains to ethoxyquin are presented. The figure is divided into three columns, each representing one AOB strain. The ammonia oxidation activity is illustrated through a linear regression plot, comparing the AOB response to different dose rates, alongside the respective control treatment. The dashed lines in each plot represent the linear regression of nitrite values (data points not shown) at each time point for each treatment. Below the plot, a summary of the modeling of the AOB response, that led to the estimation of each EC_50_ value, is provided. For the log – logistic model, one model fit with an estimate of the EC_50_ is given for each time point. For polynomial or linear models, an aggregate model fit is presented with a final estimate of the EC_50_. Detailed information about the modeling procedure can be found in the Materials and Methods section.*

*
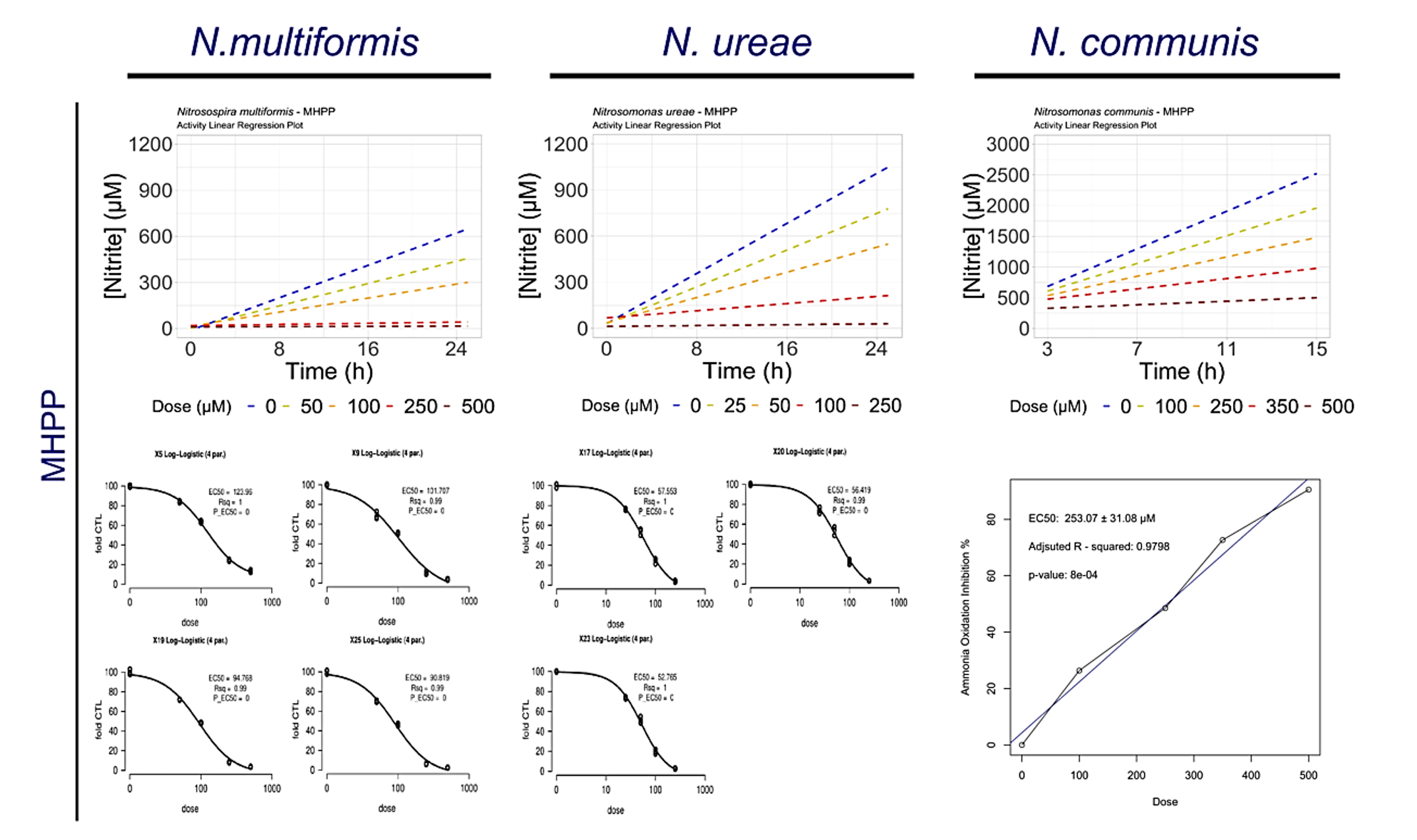
*

***Figure 12.*** ***Validation of the AOB fast – track assay with MHPP.*** *The responses of the AOB strains to MHPP are presented. The figure is divided into three columns, each representing one AOB strain. The ammonia oxidation activity is illustrated through a linear regression plot, comparing the AOB response to different dose rates, alongside the respective control treatment. The dashed lines in each plot represent the linear regression of nitrite values (data points not shown) at each time point for each treatment. Below the plot, a summary of the modeling of the AOB response, that led to the estimation of each EC_50_ value, is provided. For the log – logistic model, one model fit with an estimate of the EC_50_ is given for each time point. For polynomial or linear models, an aggregate model fit is presented with a final estimate of the EC_50_. Detailed information about the modeling procedure can be found in the Materials and Methods section.*

***
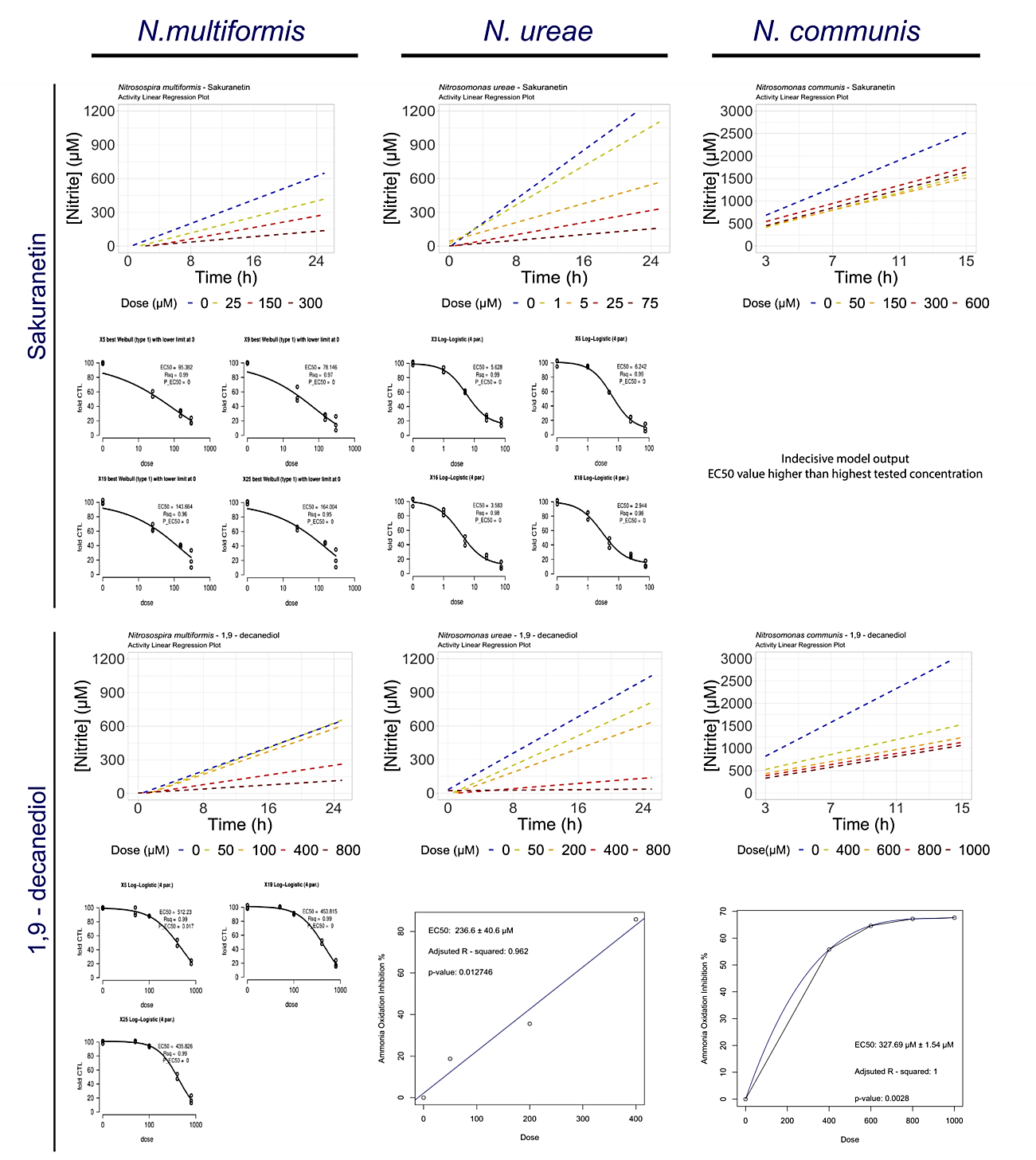
 Figure 13.*** ***Validation of the AOB fast – track assay with sakuranetin.*** *The responses of the AOB strains to sakuranetin are presented. The figure is divided into three columns, each representing one AOB strain. The ammonia oxidation activity is illustrated through a linear regression plot, comparing the AOB response to different dose rates, alongside the respective control treatment. The dashed lines in each plot represent the linear regression of nitrite values (data points not shown) at each time point for each treatment. Below the plot, a summary of the modeling of the AOB response, that led to the estimation of each EC_50_ value, is provided. For the log – logistic model, one model fit with an estimate of the EC_50_ is given for each time point. For polynomial or linear models, an aggregate model fit is presented with a final estimate of the EC_50_. Detailed information about the modeling procedure can be found in the Materials and Methods section.*

*
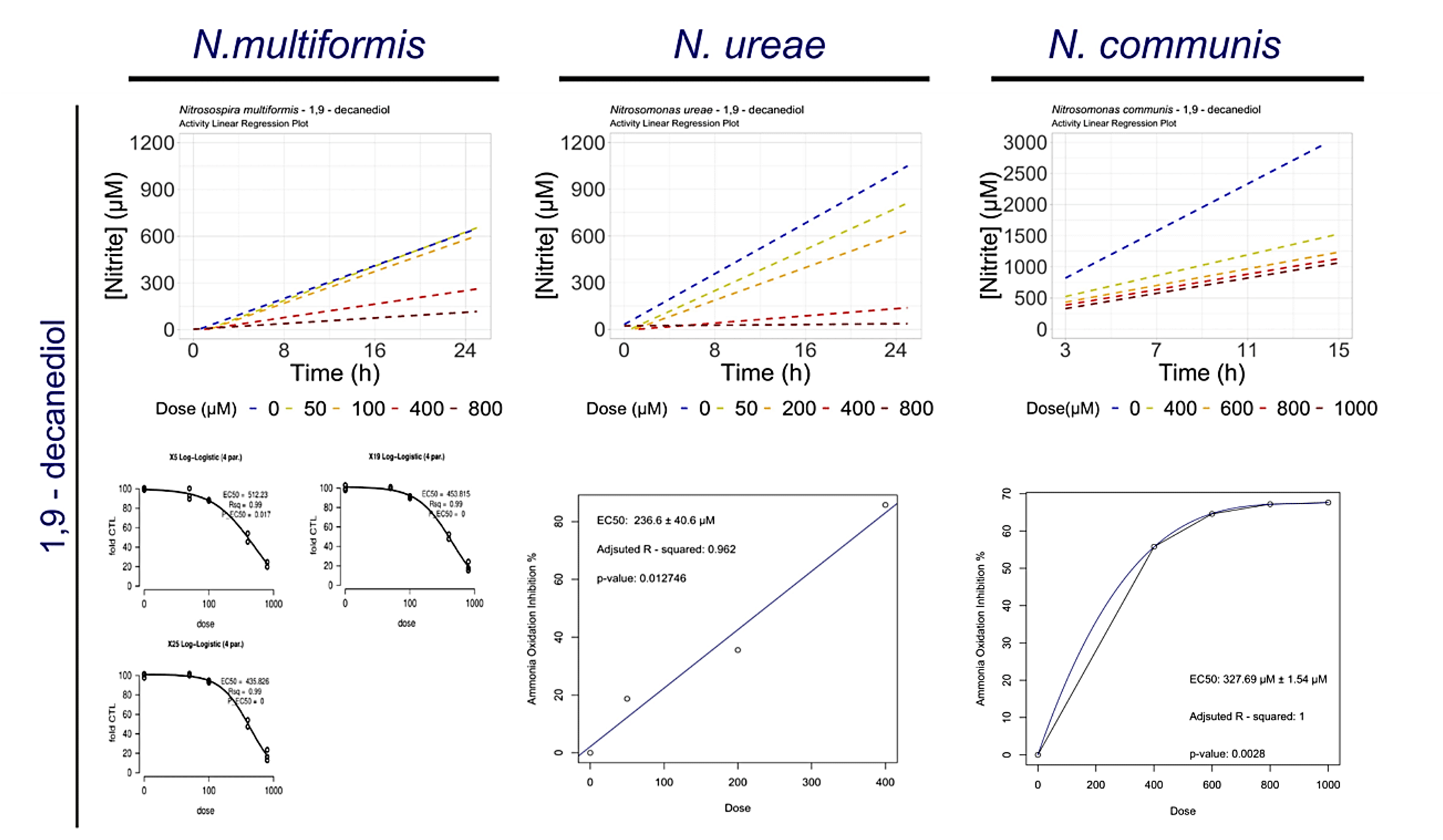
*

***Figure 14.*** ***Validation of the AOB fast – track assay with 1,9-decanediol.*** *The responses of the AOB strains to 1,9-decanediol are presented. The figure is divided into three columns, each representing one AOB strain. The ammonia oxidation activity is illustrated through a linear regression plot, comparing the AOB response to different dose rates, alongside the respective control treatment. The dashed lines in each plot represent the linear regression of nitrite values (data points not shown) at each time point for each treatment. Below the plot, a summary of the modeling of the AOB response, that led to the estimation of each EC_50_ value, is provided. For the log – logistic model, one model fit with an estimate of the EC_50_ is given for each time point. For polynomial or linear models, an aggregate model fit is presented with a final estimate of the EC_50_. Detailed information about the modeling procedure can be found in the Materials and Methods section.*

*
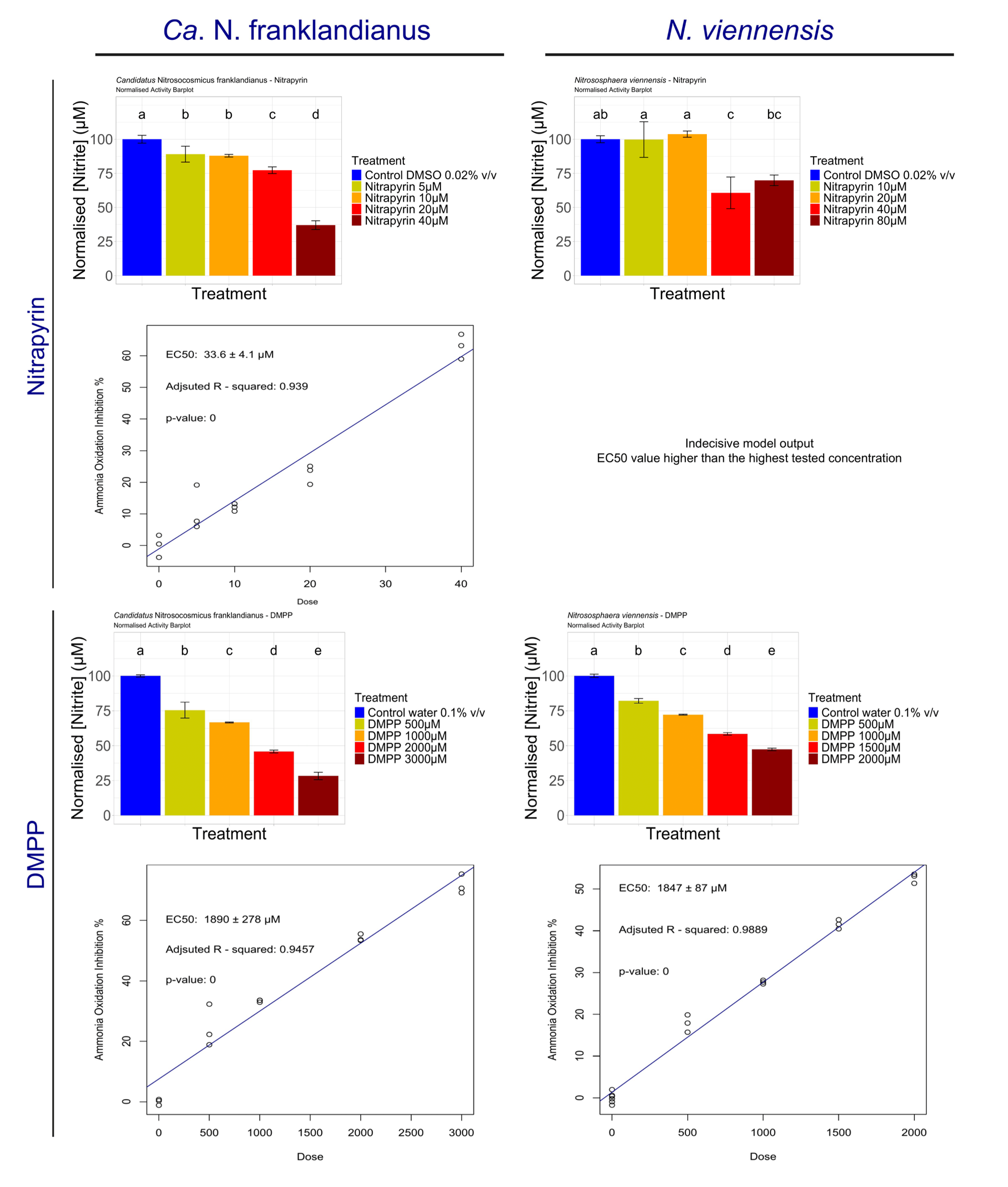
*

***Figure 15.*** ***Validation of the AOA fast – track assay with nitrapyrin.*** *The responses of the AOA strains to nitrapyrin are presented. The figure is divided into three columns, each representing one AOA strain. The normalized ammonia oxidation activity data are presented in a bar plot, comparing the AOA response to different dose rates, alongside the respective control treatment. The colours of the bars correspond to different dose rates, following the legend on the right, and lowercase letters indicate the grouping of the treatments according to the Kruskal – Wallis test. Below the plot, a summary of the modeling of the AOΑ response, that led to the estimation of each EC_50_ value, is provided, utilizing a single model fit to estimate the EC_50_. Detailed information about the modeling procedure can be found in the Materials and Methods section.*

*
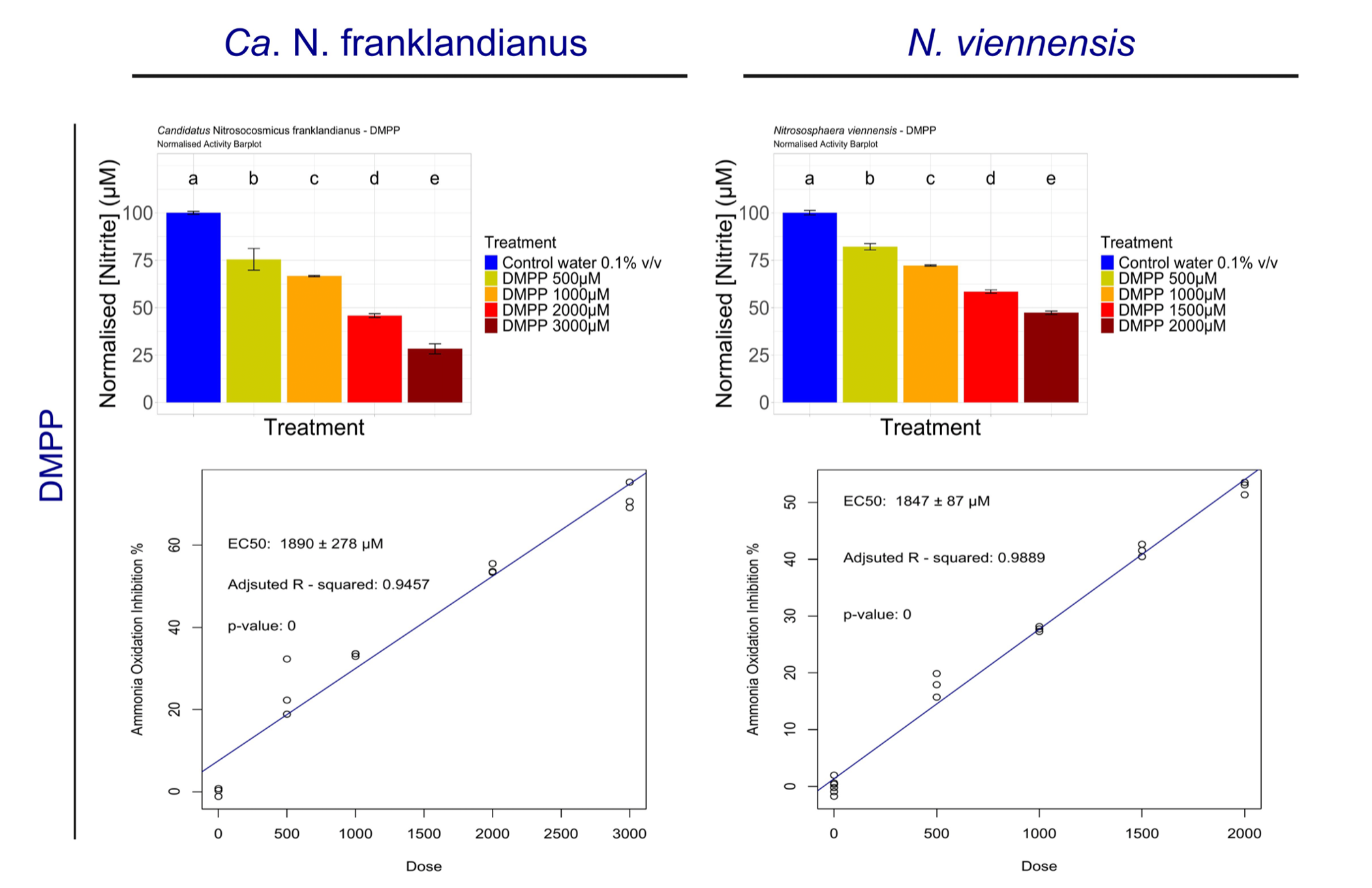
*

***Figure 16.*** ***Validation of the AOA fast – track assay with DMPP.*** *The responses of the AOA strains to DMPP are presented. The figure is divided into three columns, each representing one AOA strain. The normalized ammonia oxidation activity data are presented in a bar plot, comparing the AOA response to different dose rates, alongside the respective control treatment. The colours of the bars correspond to different dose rates, following the legend on the right, and lowercase letters indicate the grouping of the treatments according to the Kruskal – Wallis test. Below the plot, a summary of the modeling of the AOΑ response, that led to the estimation of each EC_50_ value, is provided, utilizing a single model fit to estimate the EC_50_. Detailed information about the modeling procedure can be found in the Materials and Methods section.*

*
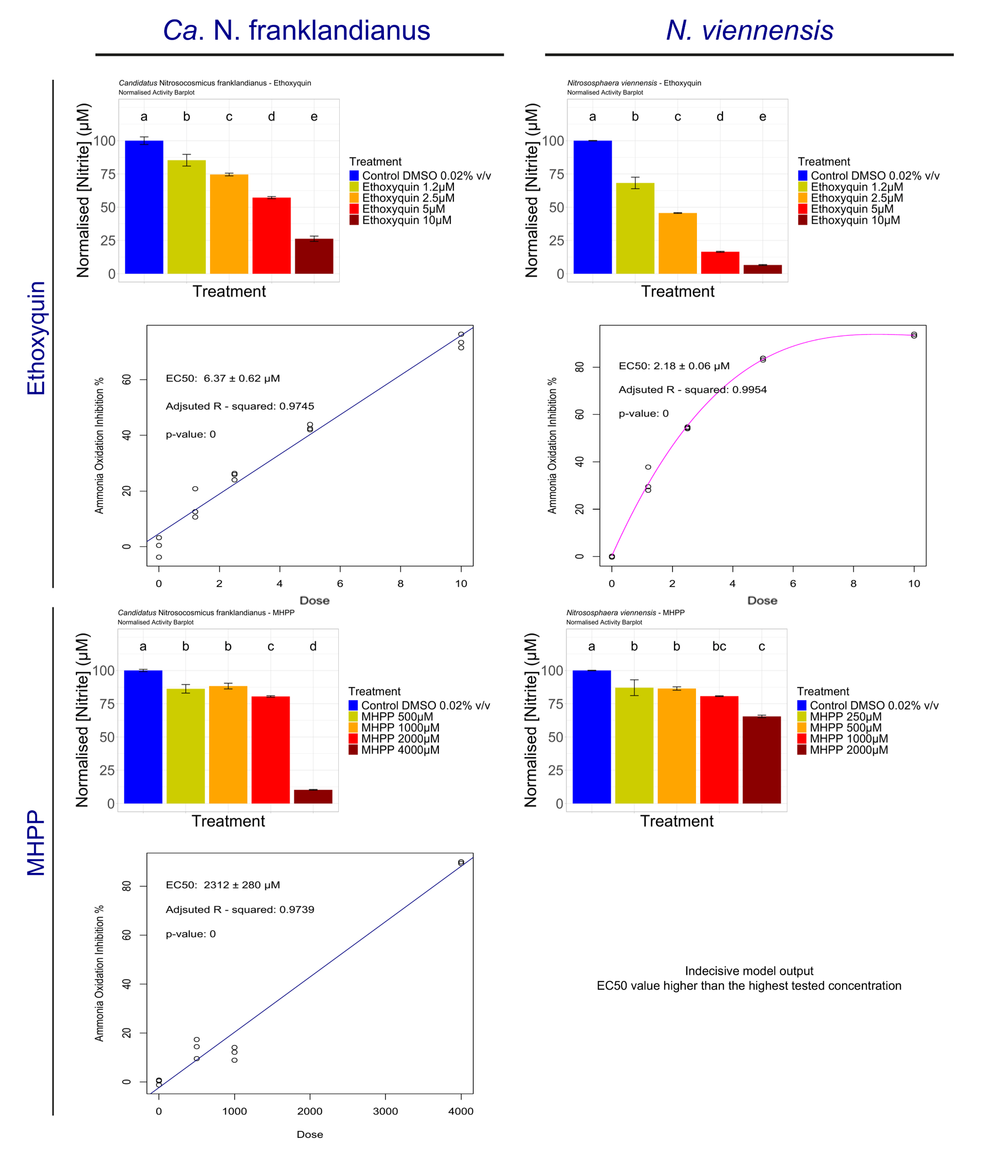
*

***Figure 17.*** ***Validation of the AOA fast – track assay with ethoxyquin.*** *The responses of the AOA strains to ethoxyquin are presented. The figure is divided into three columns, each representing one AOA strain. The normalized ammonia oxidation activity data are presented in a bar plot, comparing the AOA response to different dose rates, alongside the respective control treatment. The colours of the bars correspond to different dose rates, following the legend on the right, and lowercase letters indicate the grouping of the treatments according to the Kruskal – Wallis test. Below the plot, a summary of the modeling of the AOΑ response, that led to the estimation of each EC_50_ value, is provided, utilizing a single model fit to estimate the EC_50_. Detailed information about the modeling procedure can be found in the Materials and Methods section.*

*
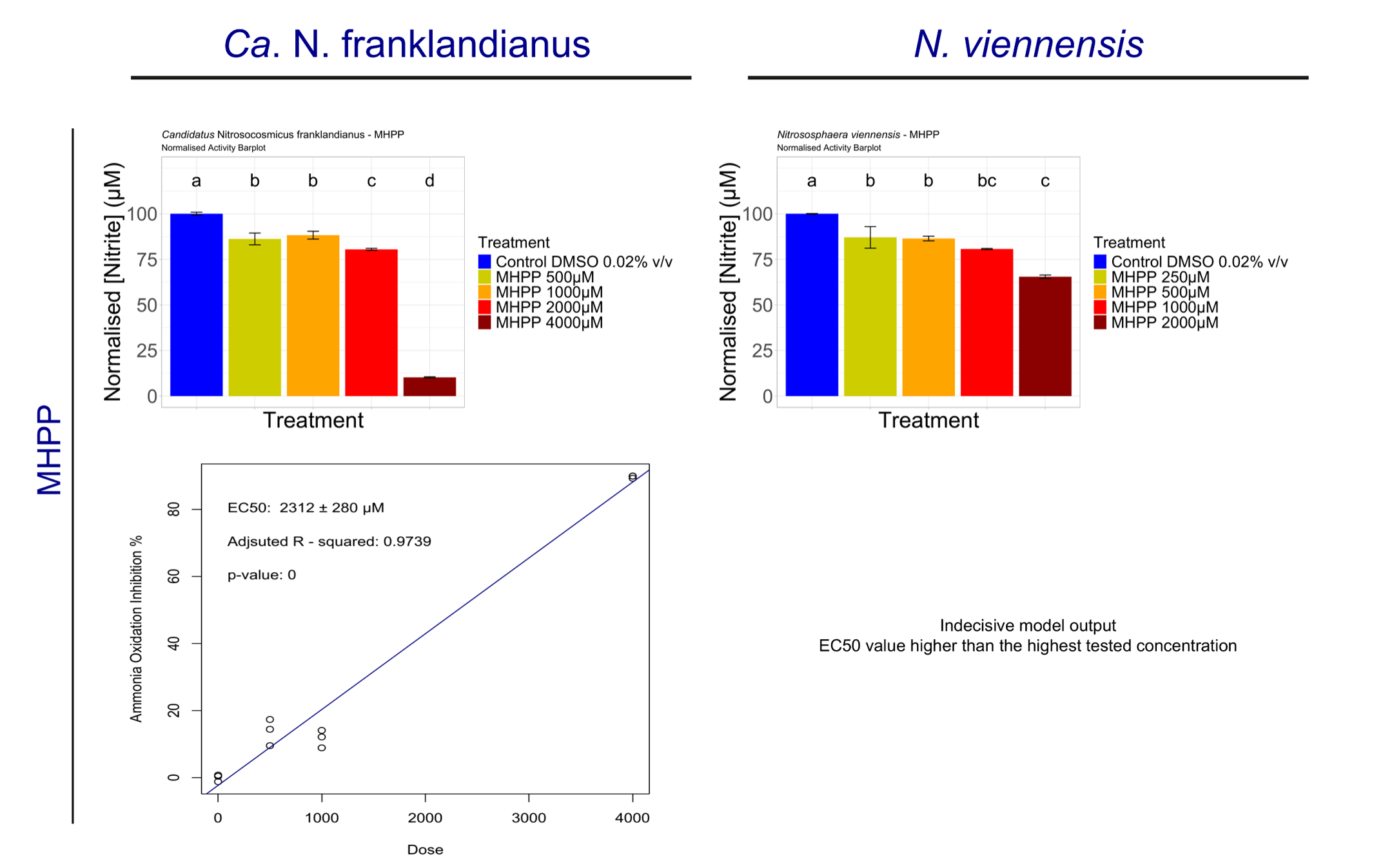
*

***Figure 18.*** ***Validation of the AOA fast – track assay with MHPP.*** *The responses of the AOA strains to MHPP are presented. The figure is divided into three columns, each representing one AOA strain. The normalized ammonia oxidation activity data are presented in a bar plot, comparing the AOA response to different dose rates, alongside the respective control treatment. The colours of the bars correspond to different dose rates, following the legend on the right, and lowercase letters indicate the grouping of the treatments according to the Kruskal – Wallis test. Below the plot, a summary of the modeling of the AOΑ response, that led to the estimation of each EC_50_ value, is provided, utilizing a single model fit to estimate the EC_50_. Detailed information about the modeling procedure can be found in the Materials and Methods section.*

*
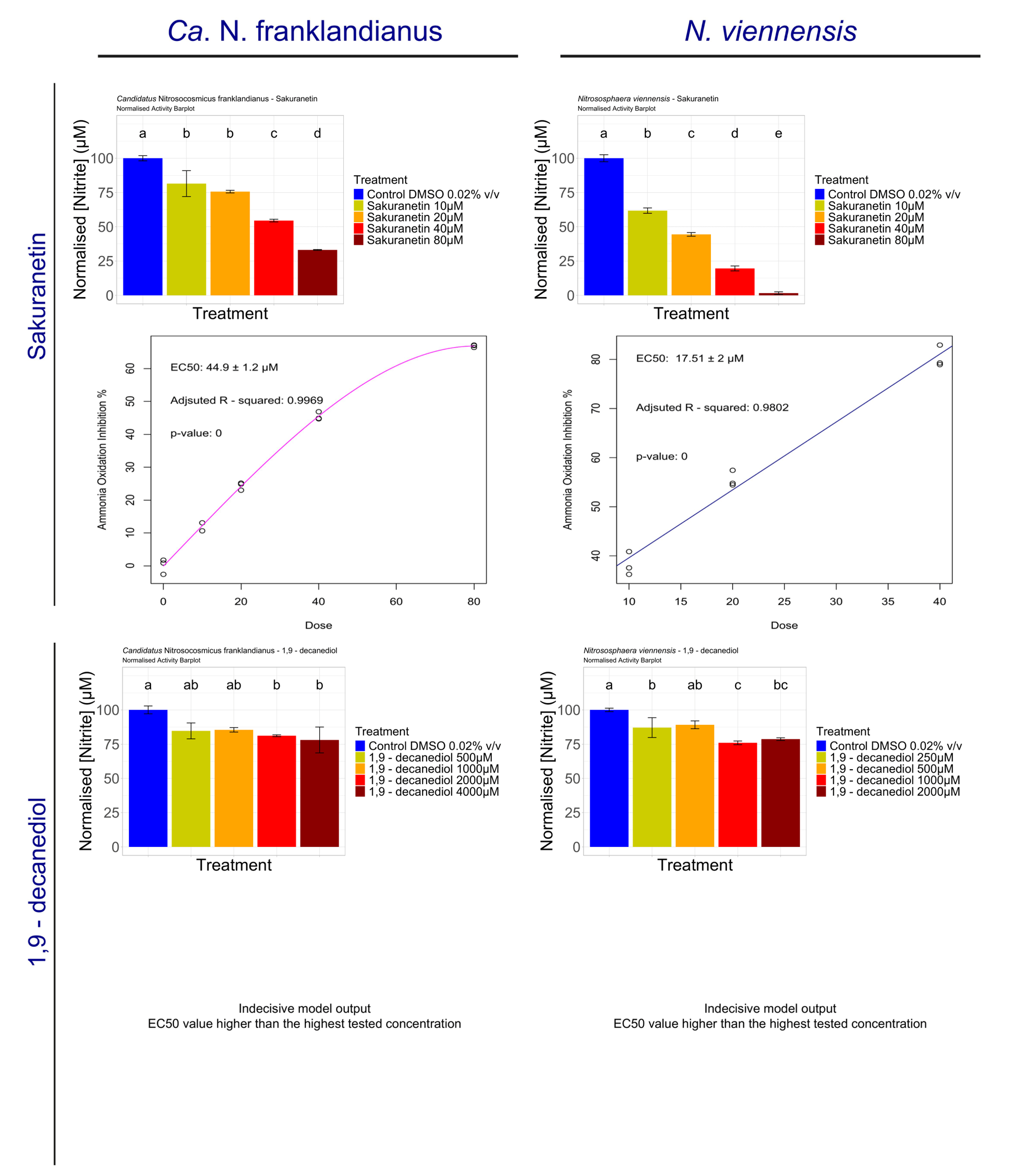
*

***Figure 19.*** ***Validation of the AOA fast – track assay with sakuranetin.*** *The responses of the AOA strains to sakuranetin are presented. The figure is divided into three columns, each representing one AOA strain. The normalized ammonia oxidation activity data are presented in a bar plot, comparing the AOA response to different dose rates, alongside the respective control treatment. The colours of the bars correspond to different dose rates, following the legend on the right, and lowercase letters indicate the grouping of the treatments according to the Kruskal – Wallis test. Below the plot, a summary of the modeling of the AOB response, that led to the estimation of each EC_50_ value, is provided, utilizing a single model fit to estimate the EC_50_. Detailed information about the modeling procedure can be found in the Materials and Methods section.*

*
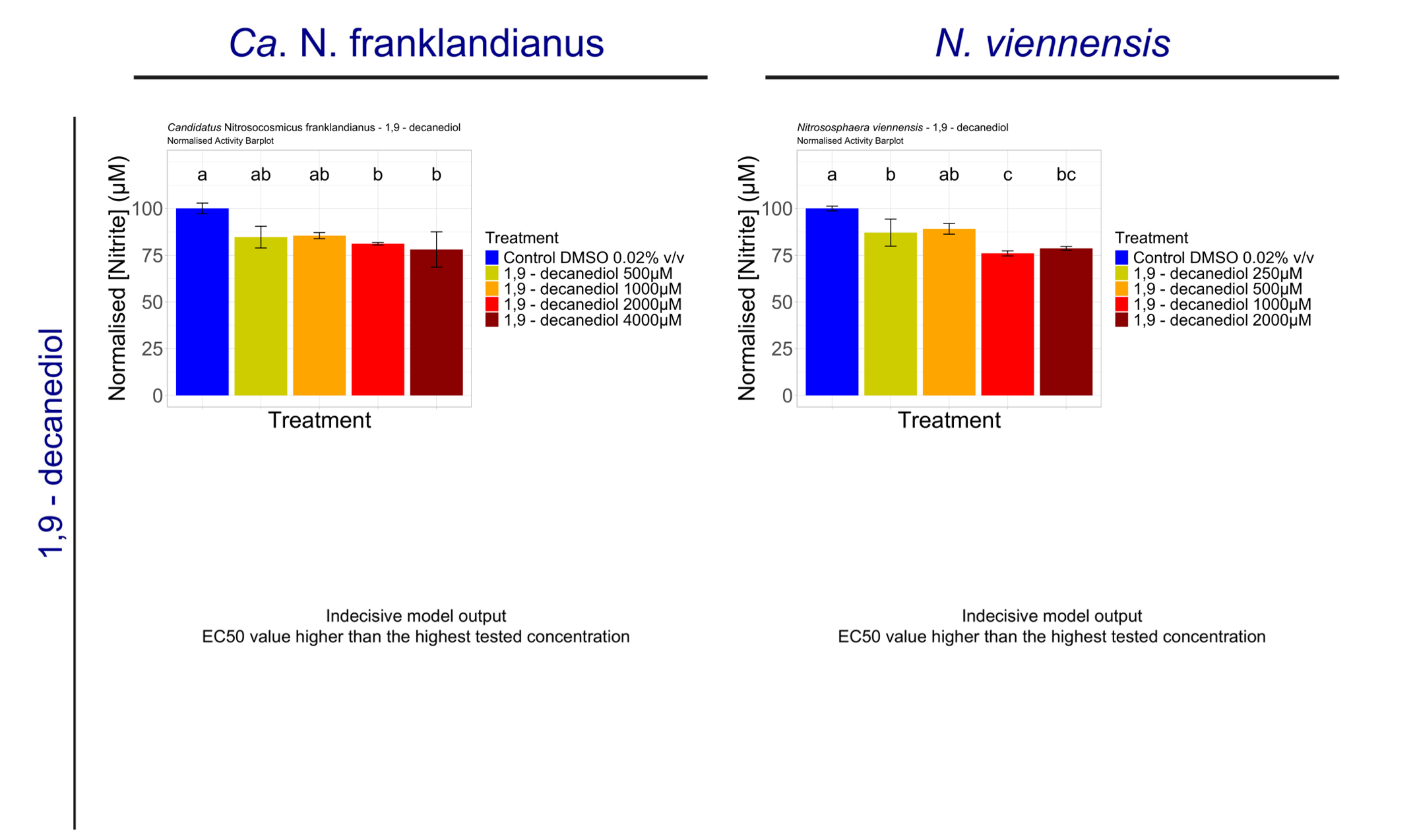
*

***Figure 20.*** ***Validation of the AOA fast – track assay with 1,9-decanediol.*** *The responses of the AOA strains to 1,9-decanediol are presented. The figure is divided into three columns, each representing one AOA strain. The normalized ammonia oxidation activity data are presented in a bar plot, comparing the AOA response to different dose rates, alongside the respective control treatment. The colours of the bars correspond to different dose rates, following the legend on the right, and lowercase letters indicate the grouping of the treatments according to the Kruskal – Wallis test. Below the plot, a summary of the modeling of the AOB response, that led to the estimation of each EC_50_ value, is provided, utilizing a single model fit to estimate the EC_50_. Detailed information about the modeling procedure can be found in the Materials and Methods section.*

*
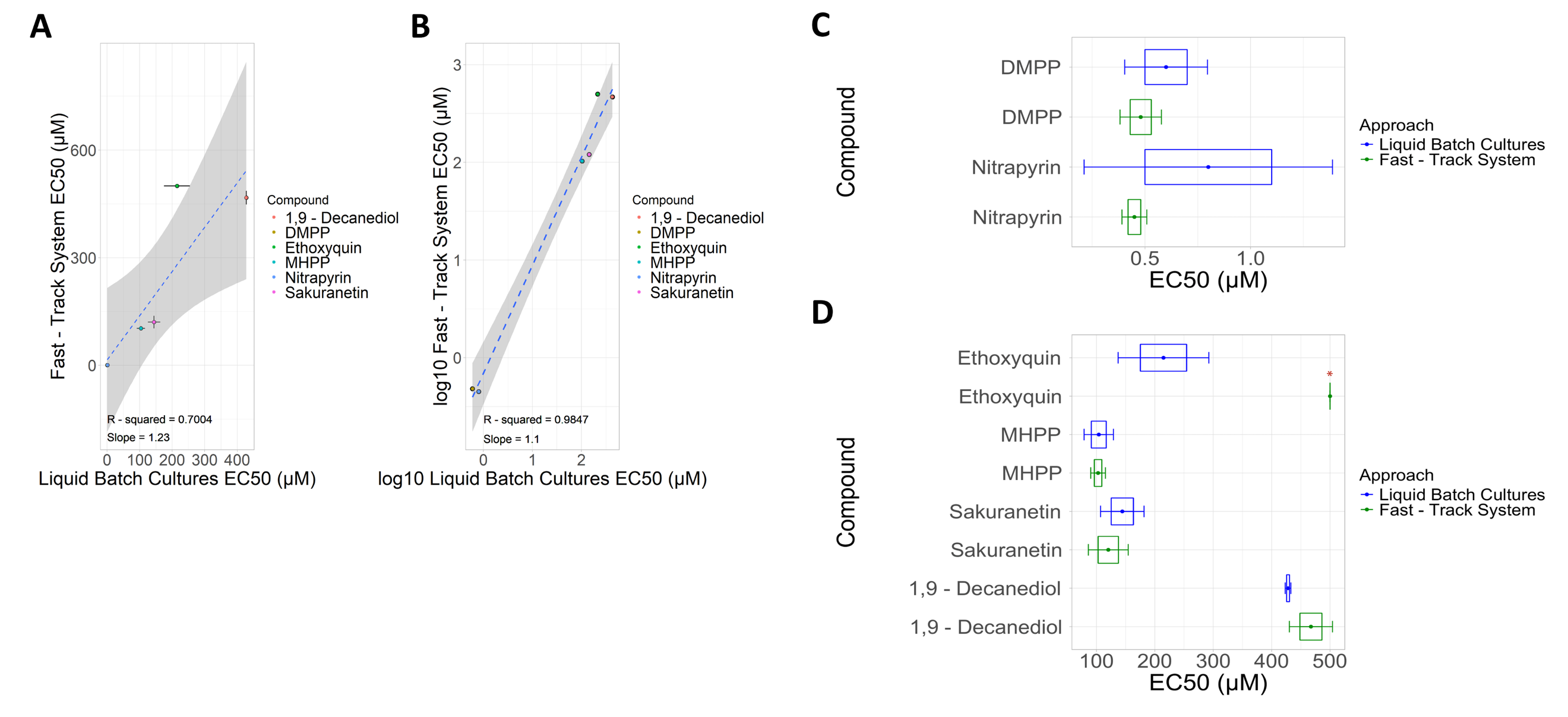
*

***Figure 21.*** ***Correlation of EC_50_ values between the fast-track and established liquid batch culture systems for N. multiformis. (A-B) Correlation between fast-track and literature-derived inhibition thresholds. (A)*** *Correlation plots of EC_50_ values for the tested nitrification inhibitors and* ***(B)*** *transformed plot based on the log10 of the EC_50_ values. The dashed line represents the linear regression of the fitted data, and shaded areas indicate the standard error. Error bars illustrate the –1SE < EC_50_ < +1SE range.* ***(C-D)*** ***Comparison of fast-track and literature-derived inhibition thresholds.*** *Comparison of EC_50_ values between the fast-track and established liquid batch culture systems for the tested SNIs and BNIs. Coloured points in the middle indicate the mean EC_50_ values, while blue and green boxes show the range –1SE < EC_50_ < +1SE. Error bars represent the range –1.96SE < EC_50_ < +1.96SE, providing a 95% confidence interval for comparison between the means. [SE: Standard Error]. Indefinite mean EC_50_ values are indicated with a red asterisk over the bar.*

*
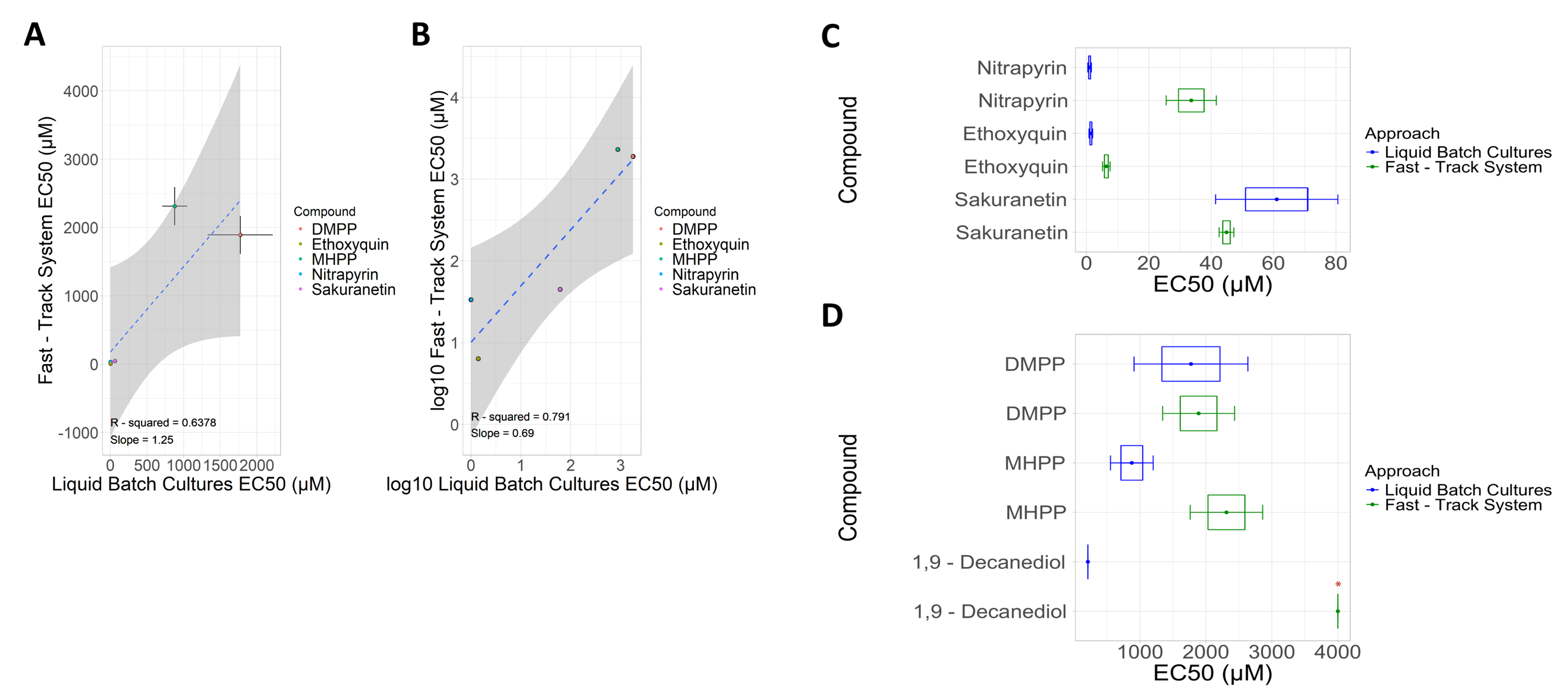
*

***Figure 22.*** ***Correlation of EC_50_ values between the fast-track and established liquid batch culture systems for “Ca.* N. franklandianus*”. (A-B) Correlation between fast-track and literature-derived inhibition thresholds. (A)*** *Correlation plots of EC_50_ values for the tested nitrification inhibitors and* ***(B)*** *transformed plot based on the log10 of the EC_50_ values. The dashed line represents the linear regression of the fitted data, and shaded areas indicate the standard error. Error bars illustrate the –1SE < EC_50_ < +1SE range.* ***(C-D)*** ***Comparison of fast-track and literature-derived inhibition thresholds.*** *Comparison of EC_50_ values between the fast-track and established liquid batch culture systems for the tested SNIs and BNIs. Coloured points in the middle indicate the mean EC_50_ values, while blue and green boxes show the range –1SE < EC_50_ < +1SE. Error bars represent the range –1.96SE < EC_50_ < +1.96SE, providing a 95% confidence interval for comparison between the means. [SE: Standard Error]. Indefinite mean EC_50_ values are indicated with a red asterisk over the bar. 1,9 – decanediol was excluded from the analysis, as inclusion of the respective data resulted in a low-quality regression.*

*
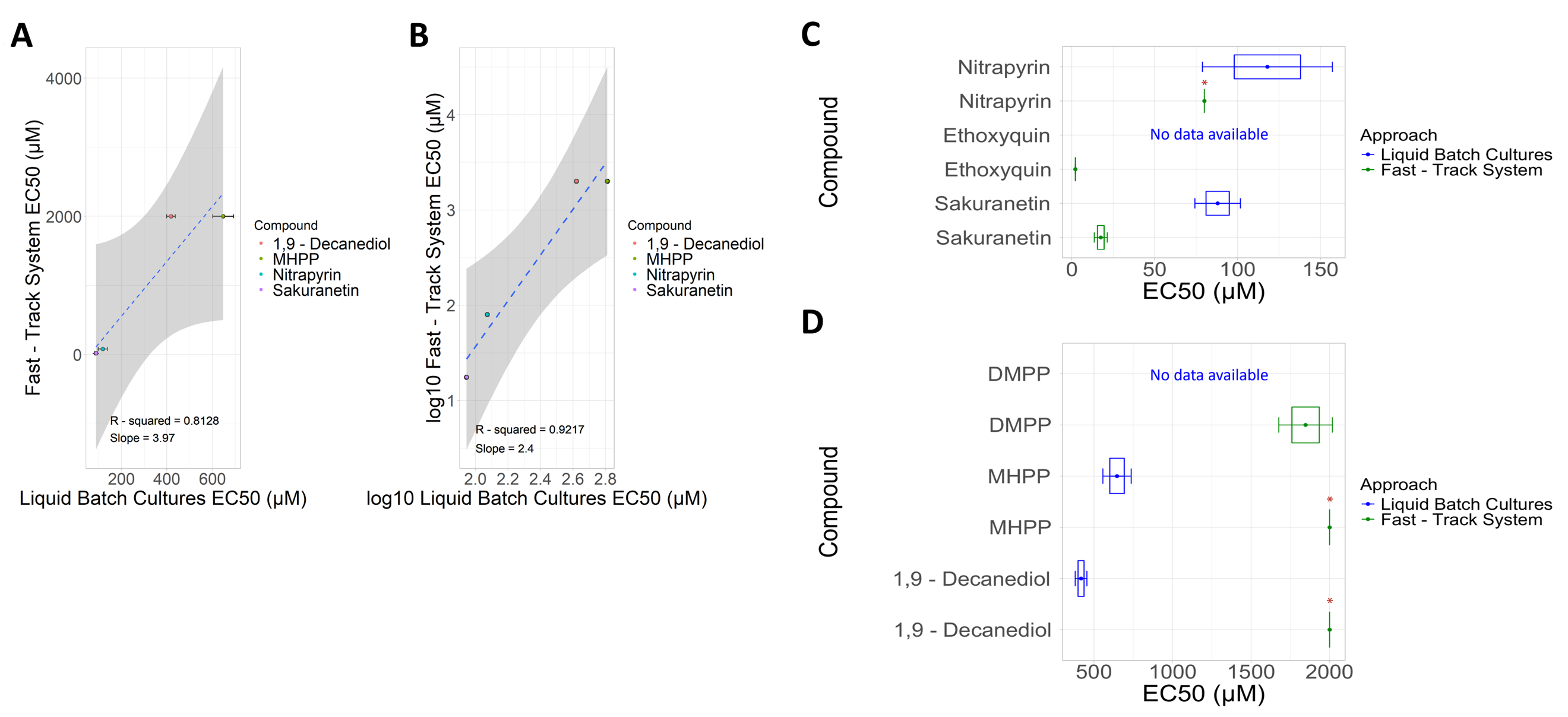
*

***Figure 23.*** ***Correlation of EC_50_ values between the fast-track and established liquid batch culture systems for N. viennensis. (A-B) Correlation between fast-track and literature-derived inhibition thresholds. (A)*** *Correlation plots of EC_50_ values for the tested nitrification inhibitors and (B) transformed plot based on the log10 of the EC_50_ values. The dashed line represents the linear regression of the fitted data, and shaded areas indicate the standard error. Error bars illustrate the –1SE < EC_50_ < +1SE range.* ***(C-D)*** ***Comparison of fast-track and literature-derived inhibition thresholds.*** *Comparison of EC_50_ values between the fast-track and established liquid batch culture systems for the tested SNIs and BNIs. Coloured points in the middle indicate the mean EC_50_ values, while blue and green boxes show the range –1SE < EC_50_ < +1SE. Error bars represent the range –1.96SE < EC_50_ < +1.96SE, providing a 95% confidence interval for comparison between the means. [SE: Standard Error]. Indefinite mean EC_50_ values are indicated with a red asterisk over the bar. DMPP and ethoxyquin were excluded from the analysis, as no inhibition thresholds were found in literature for this strain.*

*
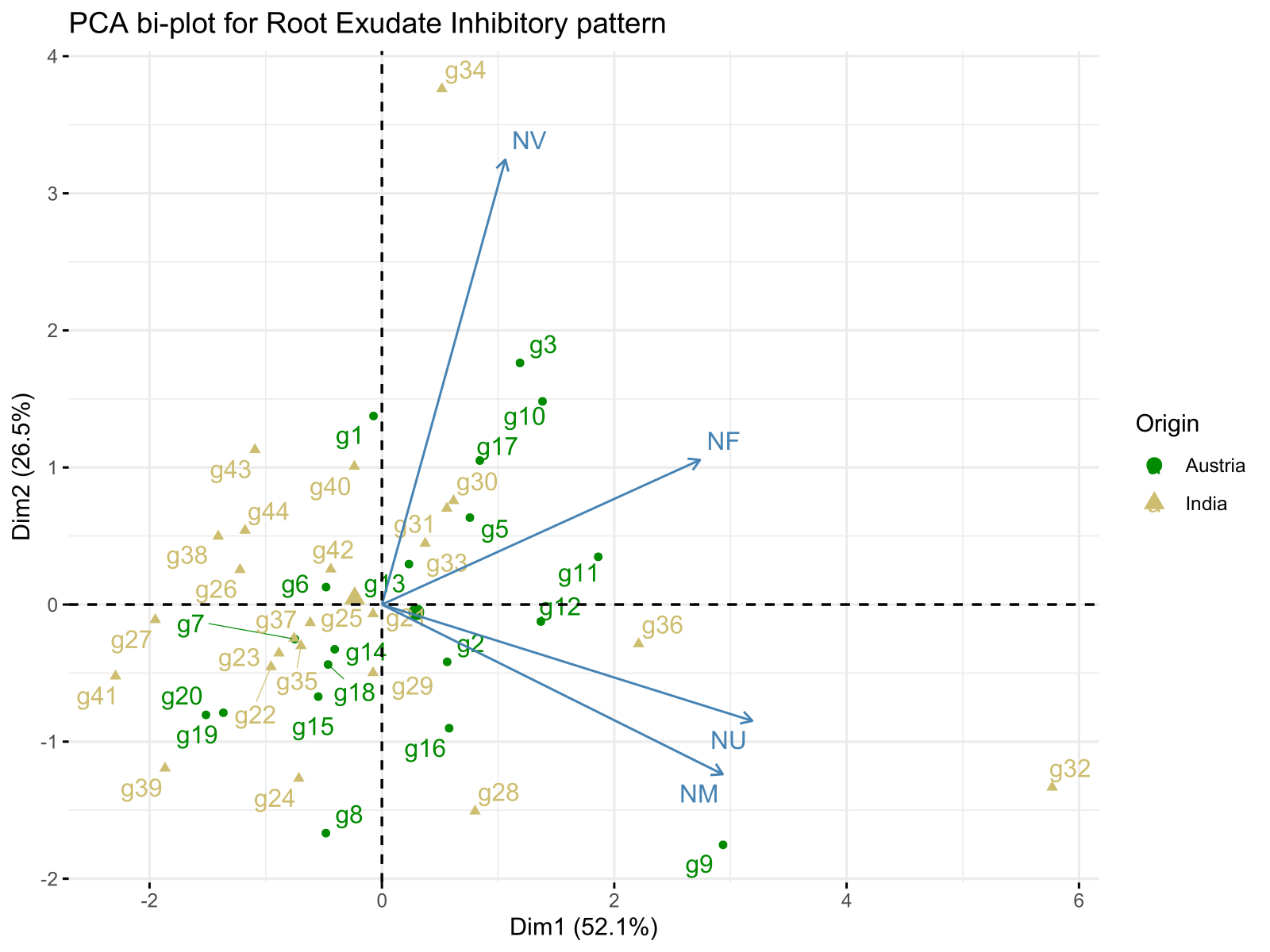
*

***Figure 24. Inhibitory activity patterns of root exudates on the tested ammonia-oxidizing microorganisms (AOM)****. Two-dimensional representation of the root exudate inhibitory activity, derived from a four-variable dataset of the ammonia oxidation inhibition percentage values (AOI%) of each root exudate (RE) across four reporter AOM strains. Data points are color-coded based on the geographical origin of the respective genotypes. The percentage values on the axes indicate the proportion of the initial variance explained by the two principal components. The length of the light blue arrows reflects the contribution of each variable (AOM strain inhibition) to the first two dimensions, while the direction of the arrows serves as a proxy for correlation; arrows and data points on the same side indicate positively correlated variable-individual pairs.* *Due to limited material availability, the two most sensitive AOB (NM – N. multiformis, NU – N. ureae) were included in the screening of wheat REs, along with the two AOA strains (NV – N. viennensis, NF – “Ca.* N. franklandianus*”).*

**References**

1. Skinner FA, Walker N. 1961. Growth of Nitrosomonas europaea in batch and continuous culture. Arch Für Mikrobiol 38(4):339–49.
2. Koops HP, Böttcher B, Möller UC, Pommerening-Röser A, Stehr G. 1991. Classification of eight new species of ammonia-oxidizing bacteria: *Nitrosomonas communis* sp. nov., *Nitrosomonas ureae* sp. nov., *Nitrosomonas aestuarii* sp. nov., *Nitrosomonas marina* sp. nov., *Nitrosomonas nitrosa* sp. nov., *Nitrosomonas eutropha* sp. nov., *Nitrosomonas oligotropha* sp. nov. and *Nitrosomonas halophila* sp. nov. Microbiology 137(7):1689–99.
3. Reyes C, Hodgskiss LH, Baars O, Kerou M, Bayer B, Schleper C, Kraemer SM. 2020. Copper limiting threshold in the terrestrial ammonia oxidizing archaeon N*itrososphaera viennensis.* Res Microbiol 171(3):134–42.
4. Shinn MB. 1941. ACS Publications. American Chemical Society. Colorimetric method for determination of nitrate.
5. Zerulla W, Barth T, Dressel J, Erhardt K, Horchler von Locquenghien K, Pasda G, Rädle M, Wissemeier A. 2001. 3,4-Dimethylpyrazole phosphate (DMPP) – a new nitrification inhibitor for agriculture and horticulture. Biol Fertil Soils 34(2):79–84.
6. Goring CA. 1962. Control of nitrification by 2-chloro-6-(trichloro-methyl) pyridine. Soil Sci 93(3):211.
7. Papadopoulou ES, Bachtsevani E, Lampronikou E, Adamou E, Katsaouni A, Vasileiadis S, Thion C, Menkissoglu-Spiroudi U, Nicol GW, Karpouzas DG. Comparison of Novel and Established Nitrification Inhibitors Relevant to Agriculture on Soil Ammonia- and Nitrite-Oxidizing Isolates. Front Microbiol 11:581283.
8. Zakir HAKM, Subbarao GV, Pearse SJ, Gopalakrishnan S, Ito O, Ishikawa T, Kawano N, Nakahara K, Yoshihashi AT, Ono H, Yoshida M. 2008. Detection, isolation and characterization of a root-exuded compound, methyl 3-(4-hydroxyphenyl) propionate, responsible for biological nitrification inhibition by sorghum (Sorghum bicolor). New Phytol 180(2):442–51.
9. Subbarao GV, Nakahara K, Ishikawa T, Ono H, Yoshida M, Yoshihashi T, Zhu Y, Zakir HAKM, Deshpande SP, Hash CT, Sahrawat KL. 2013. Biological nitrification inhibition (BNI) activity in sorghum and its characterization. Plant Soil 366(1):243–59.
10. Sun L, Lu Y, Yu F, Kronzucker HJ, Shi W. 2016. Biological nitrification inhibition by rice root exudates and its relationship with nitrogen-use efficiency. New Phytol 212(3):646–56.
11. Kolovou M, Panagiotou D, Süße L, Loiseleur O, Williams S, Karpouzas DG, Papadopoulou ES. 2023. Assessing the activity of different plant-derived molecules and potential biological nitrification inhibitors on a range of soil ammonia- and nitrite-oxidizing strains. Appl Environ Microbiol 89(11):e01380-23.
12. Wright CL, Schatteman A, Crombie AT, Murrell JC, Lehtovirta-Morley LE. 2020. Inhibition of Ammonia Monooxygenase from Ammonia-Oxidizing Archaea by Linear and Aromatic Alkynes. Appl Environ Microbiol 86(9):e02388-19.
13. Zhao J, Bello MO, Meng Y, Prosser JI, Gubry-Rangin C. 2020. Selective inhibition of ammonia oxidising archaea by simvastatin stimulates growth of ammonia oxidising bacteria. Soil Biol Biochem 141:107673.
14. Rotthauwe JH, Witzel KP, Liesack W. 1997. The ammonia monooxygenase structural gene amoA as a functional marker: molecular fine-scale analysis of natural ammonia-oxidizing populations. Appl Environ Microbiol 63(12):4704–12.
15. Bachtsevani E, Papazlatani CV, Rousidou C, Lampronikou E, Menkissoglu-Spiroudi U, Nicol GW, Karpouzas DG, Papadopoulou ES. 2021. Effects of the nitrification inhibitor 3,4-dimethylpyrazole phosphate (DMPP) on the activity and diversity of the soil microbial community under contrasting soil pH. Biol Fertil Soils 57(8):1117–35.
16. Norton JM, Klotz MG, Stein LY, Arp DJ, Bottomley PJ, Chain PSG, Hauser LJ, Land ML, Larimer FW, Shin MW, Starkenburg SR. 2008. Complete Genome Sequence of *Nitrosospira multiformis*, an Ammonia-Oxidizing Bacterium from the Soil Environment. Appl Environ Microbiol 74(11):3559–72.
17. Kozlowski JA, Kits KD, Stein LY. 2016a. Complete Genome Sequence of *Nitrosomonas ureae* Strain Nm10, an Oligotrophic Group 6a Nitrosomonad. Genome Announc 4(2):10.1128/genomea.00094-16.
18. Kozlowski JA, Kits KD, Stein LY. 2016b. Genome Sequence of *Nitrosomonas communis* Strain Nm2, a Mesophilic Ammonia-Oxidizing Bacterium Isolated from Mediterranean Soil. Genome Announc 4(1):10.1128/genomea.01541-15.
19. Malits A, Ibarbalz FM, Martín J, Flombaum P. 2023. Higher biotic than abiotic natural variability of the plankton ecosystem revealed by a time series along a subantarctic transect. J Mar Syst 238:103843.
20. R Core Team. 2020. R: A Language and Environment for Statistical Computing. Austria: R Foundation for Statistical Computing.
21. Wickham H. 2016. ggplot2: Elegant Graphics for Data Analysis. Springer-Verlag New York.
22. Wickham H, Bryan J. 2023. readxl: Read Excel Files. R package version 1.4.3. <https://CRAN.R-project.org/package=readxl>
23. Wickham H, François R, Henry L, Müller K, Vaughan, D. 2023. dplyr: A Grammar of Data Manipulation. R package version 1.1.4. <https://CRAN.R-project.org/package=dplyr>
24. Müller K, Wickham H. 2023. tibble: Simple Data Frames. R package version 3.2.1. <https://CRAN.R-project.org/package=tibble>
25. Kassambara A. 2023. ggpubr: 'ggplot2' Based Publication Ready Plots. R package version 0.6.0, <https://CRAN.R-project.org/package=ggpubr>
26. Pedersen T. 2024. patchwork: The Composer of Plots. R package version 1.2.0
27. Caglar MU, Teufel AI, Wilke CO. 2018. Sicegar: R package for sigmoidal and double-sigmoidal curve fitting. PeerJ 6:e4251. <https://doi.org/10.7717/peerj.4251>
28. Powell SJ, Prosser JI. 1986. Effect of copper on inhibition by nitrapyrin of growth of *Nitrosomonas europaea.* Current Microbiology *14(3):177–179.* 
    <https://doi.org/10.1007/bf01568371>
29. Kaur-Bhambra J, Wardak DLR, Prosser JI, Gubry-Rangin C. 2022. Revisiting plant biological nitrification inhibition efficiency using multiple archaeal and bacterial ammonia-oxidising cultures. Biol Fertil Soils 58(3):241–9.
30. Ritz C, Baty F, Streibig JC, Gerhard D. 2016. Dose-response analysis using R. PLoS One 10: e0146021. <https://doi.org/10.1371/journal.pone.0146021>
31. Greenwell BM, Kabban CMS. 2014. “investr: An R Package for Inverse Estimation.”. The R Journal 6(1):90–100. <https://doi.org/10.32614/RJ-2014-009>
32. de Mendiburu F. 2023. agricolae: Statistical Procedures for Agricultural Research. R package version 1.3-7, <https://CRAN.R-project.org/package=agricolae>
33. Kassambara A. 2023. rstatix: Pipe-Friendly Framework for Basic Statistical Tests. R package version 0.7.2
34. Gu Z, Eils R, Schlesner M. 2016. Complex heatmaps reveal patterns and correlations in multidimensional genomic data. Bioinformatics (Oxford, England) *32*(18):2847–2849. <https://doi.org/10.1093/bioinformatics/btw313>
35. Galili T. 2015. dendextend: an R package for visualizing, adjusting and comparing trees of hierarchical clustering. Bioinformatics (Oxford, England) *31*(22):3718–3720. <https://doi.org/10.1093/bioinformatics/btv428>
36. Oksanen J, Simpson G, Blanchet F, Kindt R, Legendre P, Minchin P, O'Hara R, Solymos P, Stevens M, Szoecs E, Wagner H, Barbour M, Bedward M, Bolker B, Borcard D, Carvalho G, Chirico M, De Caceres M, Durand S, Evangelista H, FitzJohn R, Friendly M, Furneaux B, Hannigan G, Hill M, Lahti L, McGlinn D, Ouellette M, Ribeiro Cunha E, Smith T, Stier A, Ter Braak C, Weedon J. 2022. vegan: Community Ecology Package. R package version 2.6-4, <https://CRAN.R-project.org/package=vegan>
37. Kassambara A, Mundt F 2020. factoextra: Extract and Visualize the Results of Multivariate Data Analyses. R package version 1.0.7. <https://CRAN.R-project.org/package=factoextra>
38. Weckwerth, W., Wenzel, K. & Fiehn, O. Process for the integrated extraction identification, and quantification of metabolites, proteins and RNA to reveal their co-regulation in biochemical networks. Proteomics 4, 78-83 (2004). https://doi.org/10.1002/pmic.200200500
39. Obermeyer, G., Fragner, L., Lang, V. & Weckwerth, W. Dynamic Adaption of Metabolic Pathways during Germination and Growth of Lily Pollen Tubes after Inhibition of the Electron Transport Chain. Plant Physiology 162, 1822-1833 (2013). https://doi.org/10.1104/pp.113.219857
40. Schindler, F. et al. Dissecting Metabolism of Leaf Nodules in Ardisia crenata and Psychotria punctata. Front Mol Biosci 8, 683671 (2021). https://doi.org/10.3389/fmolb.2021.683671
41. Chambers, M. C. et al. A cross-platform toolkit for mass spectrometry and proteomics. Nat Biotechnol 30, 918-920 (2012). https://doi.org/10.1038/nbt.2377
42. Pluskal, T., Castillo, S., Villar-Briones, A. & Oresic, M. MZmine 2: modular framework for processing, visualizing, and analyzing mass spectrometry-based molecular profile data. BMC Bioinformatics 11, 395 (2010). https://doi.org/10.1186/1471-2105-11-395
43. Heuckeroth, S. et al. Reproducible mass spectrometry data processing and compound annotation in MZmine 3. Nat Protoc 19, 2597-2641 (2024). https://doi.org/10.1038/s41596-024-00996-y
44. Horai, H. et al. MassBank: a public repository for sharing mass spectral data for life sciences. J Mass Spectrom 45, 703-714 (2010). https://doi.org/10.1002/jms.1777
45. Sawada, Y. et al. RIKEN tandem mass spectral database (ReSpect) for phytochemicals: a plant-specific MS/MS-based data resource and database. Phytochemistry 82, 38-45 (2012). https://doi.org/10.1016/j.phytochem.2012.07.007
46. Sumner, L. W., Urbanczyk-Wochniak, E. & Broeckling, C. D. Metabolomics data analysis, visualization, and integration. Methods Mol Biol 406, 409-436 (2007). [https://doi.org/10.1007/978-1-59745-535-0_2](https://doi.org/10.1007/978-1-59745-535-0_20)0
