# Supplementary material for: Natural variation of the wheat root exudate metabolome and its influence on biological nitrification inhibition activity
Source: Plant Biotechnol J. 2025 Jul 21;23(11):4755–72. doi: 10.1111/pbi.70248 (PMC12576471; doi:10.1111/pbi.70248)

Appendix S2 - Chemical structure, chromatographic peak intensity and mass spectra of the significant metabolites.

Uric acid

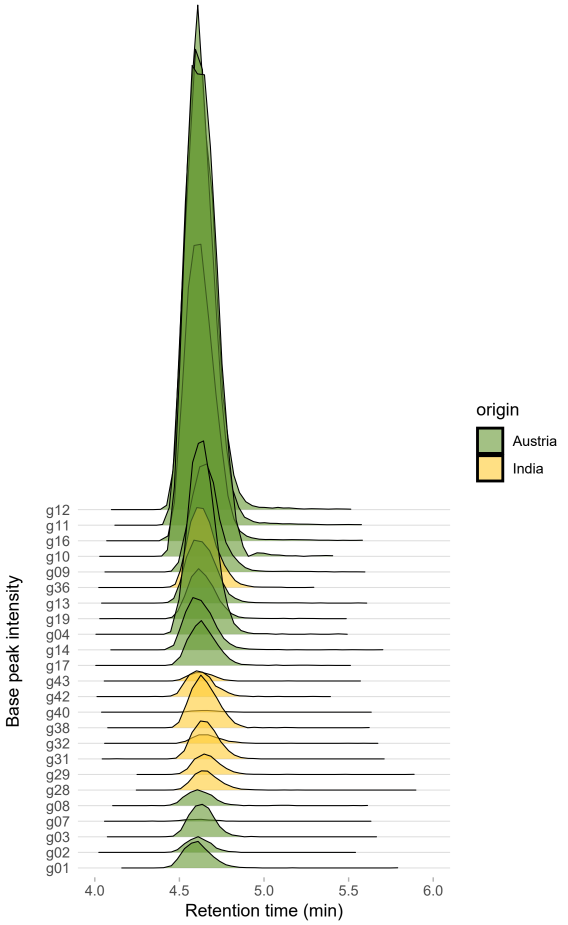

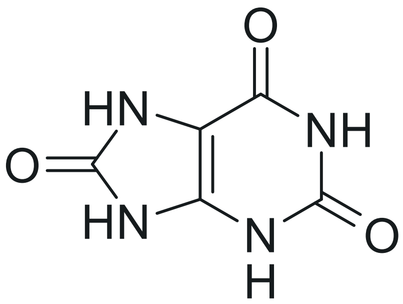

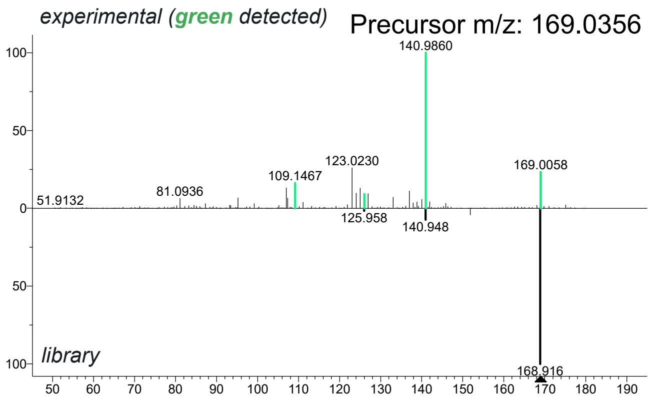

Homoserine

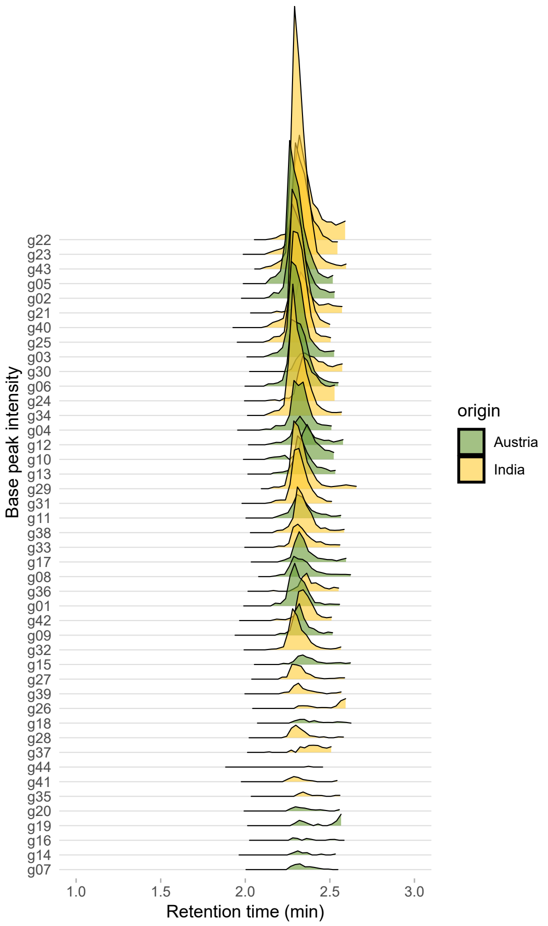

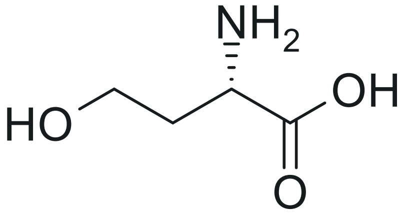

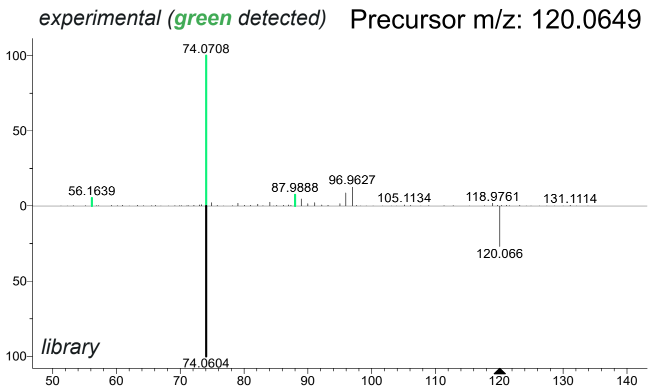

L−Histidine

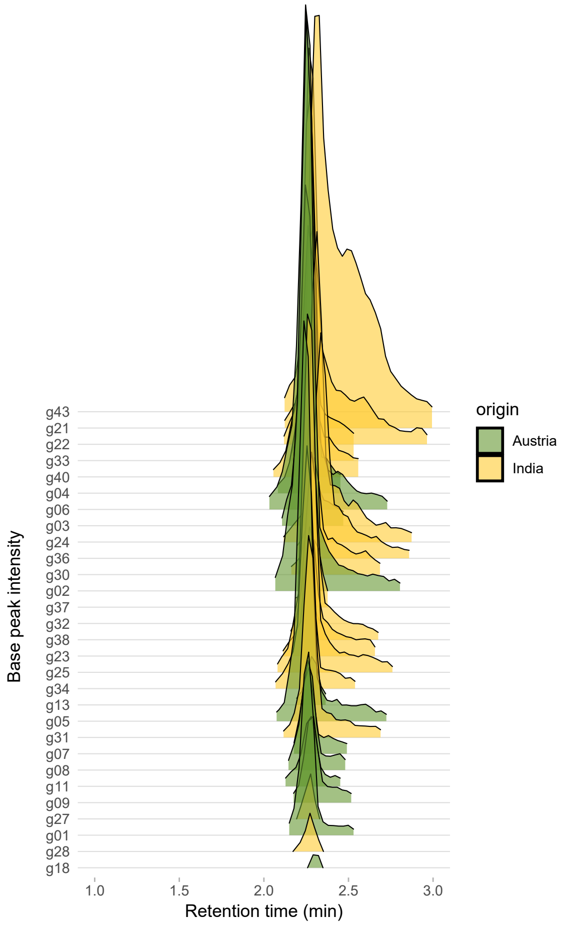

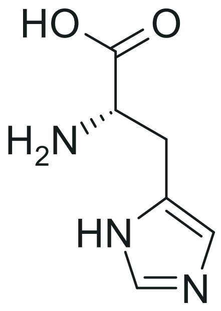

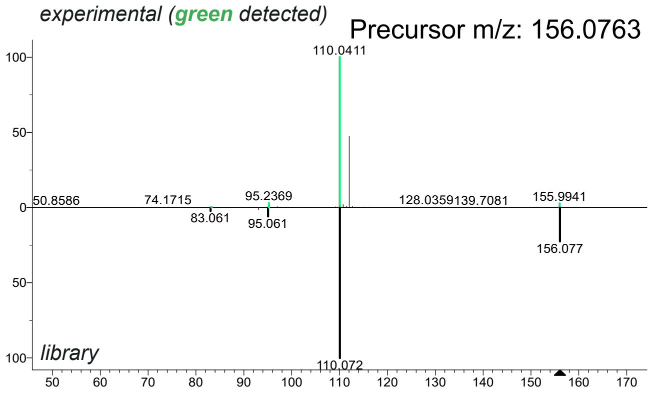

6,8C−glycosylated flavone

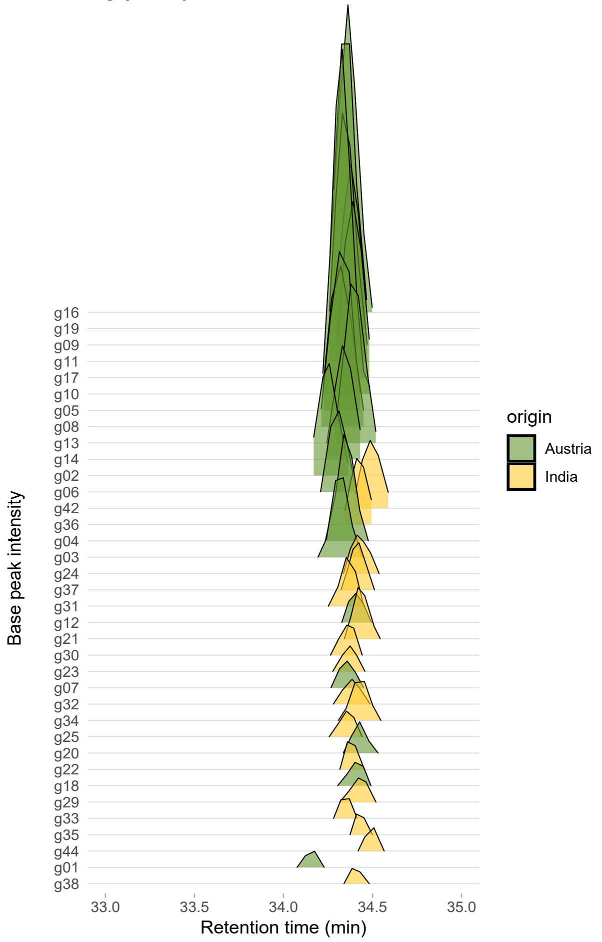

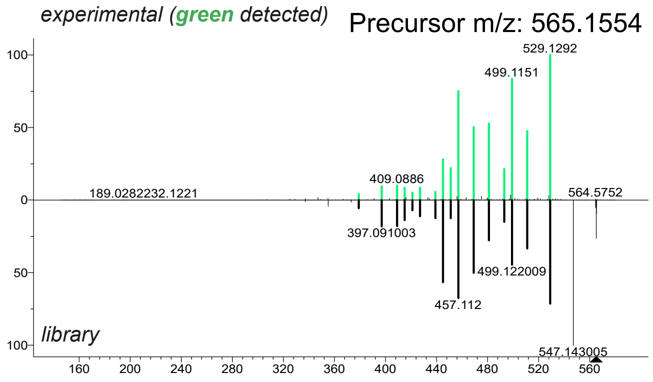

ABOA

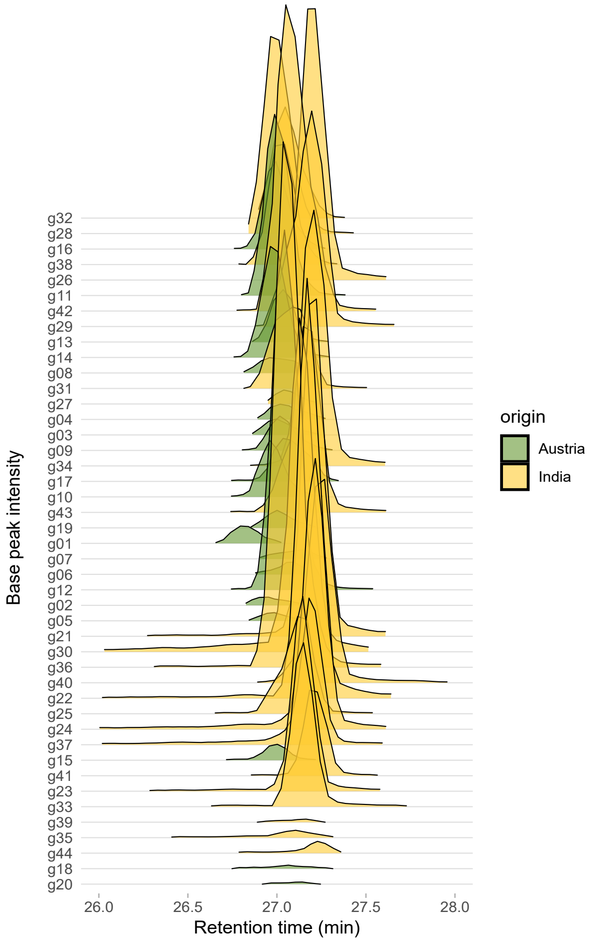

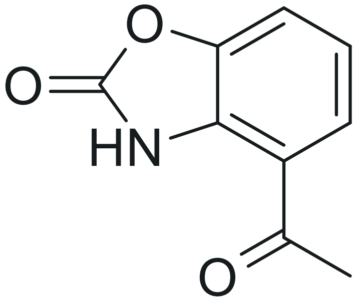

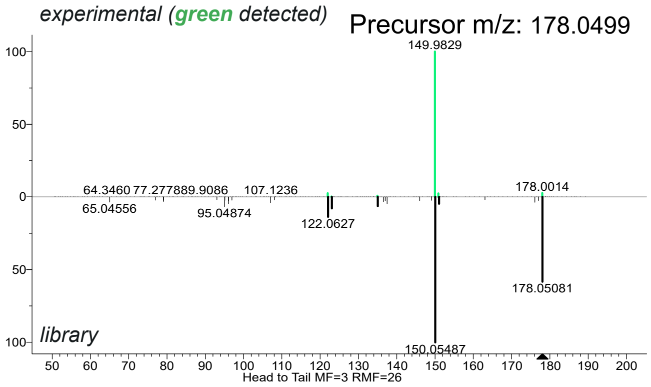

Catechol

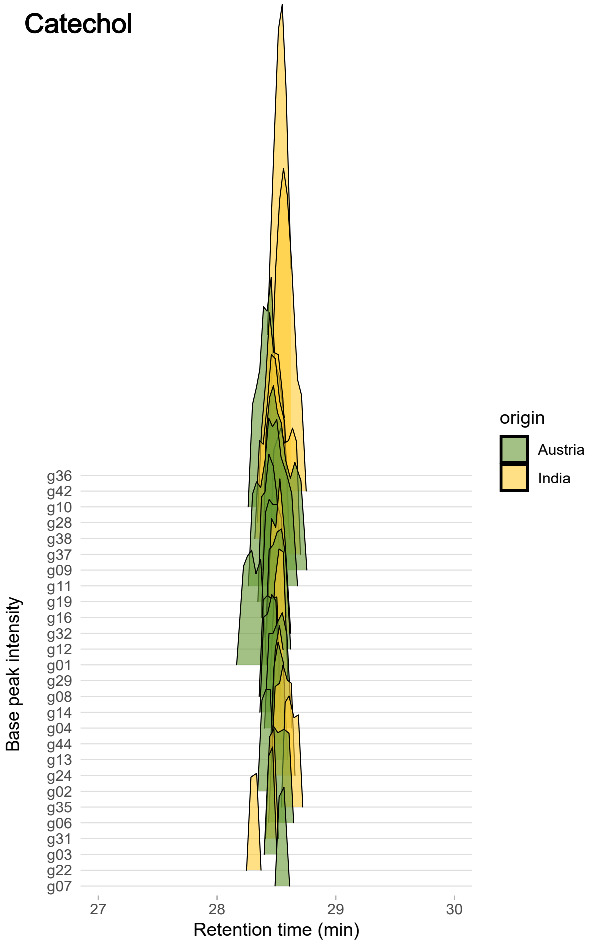

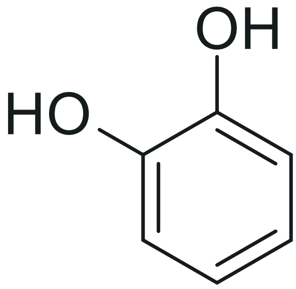

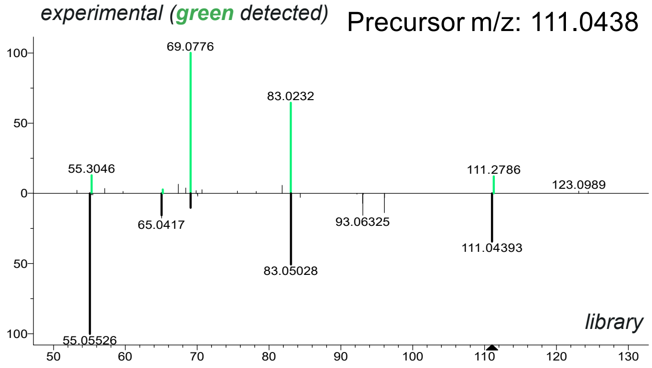

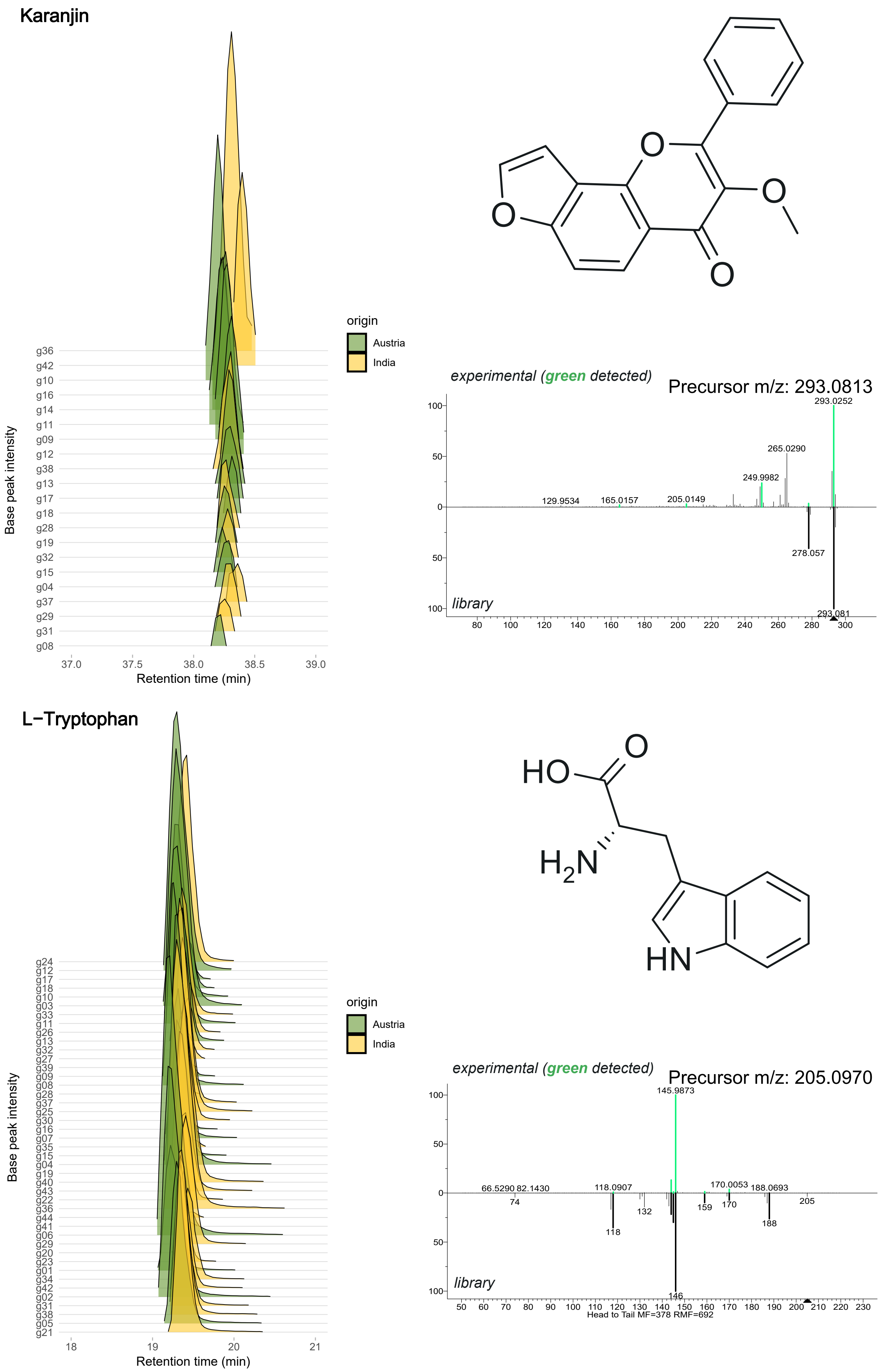
Karanjin
origin
Austria
India
Base peak intensity
Retention time (min)
g36
g42
g10
g16
g14
g11
g09
g12
g38
g13
g17
g18
g28
g19
g32
g15
g04
g37
g29
g31
g08
37.0
37.5
38.0
38.5
39.0
experimental (green detected)
Precursor m/z: 293.0813
293.0252
265.0290
249.9982
129.9534
165.0157
205.0149
278.057
293.081
library
L−Tryptophan
HO
O
HN
origin
Austria
India
Base peak intensity
Retention time (min)
18
19
20
21
experimental (green detected)
Precursor m/z: 205.0970
145.9873
66.5290 82.1430
118.0907
170.0053
188.0693
74
132
118
159
170
188
205
146
library
Head to Tail MF=378 RMF=692

Isoshaftoside

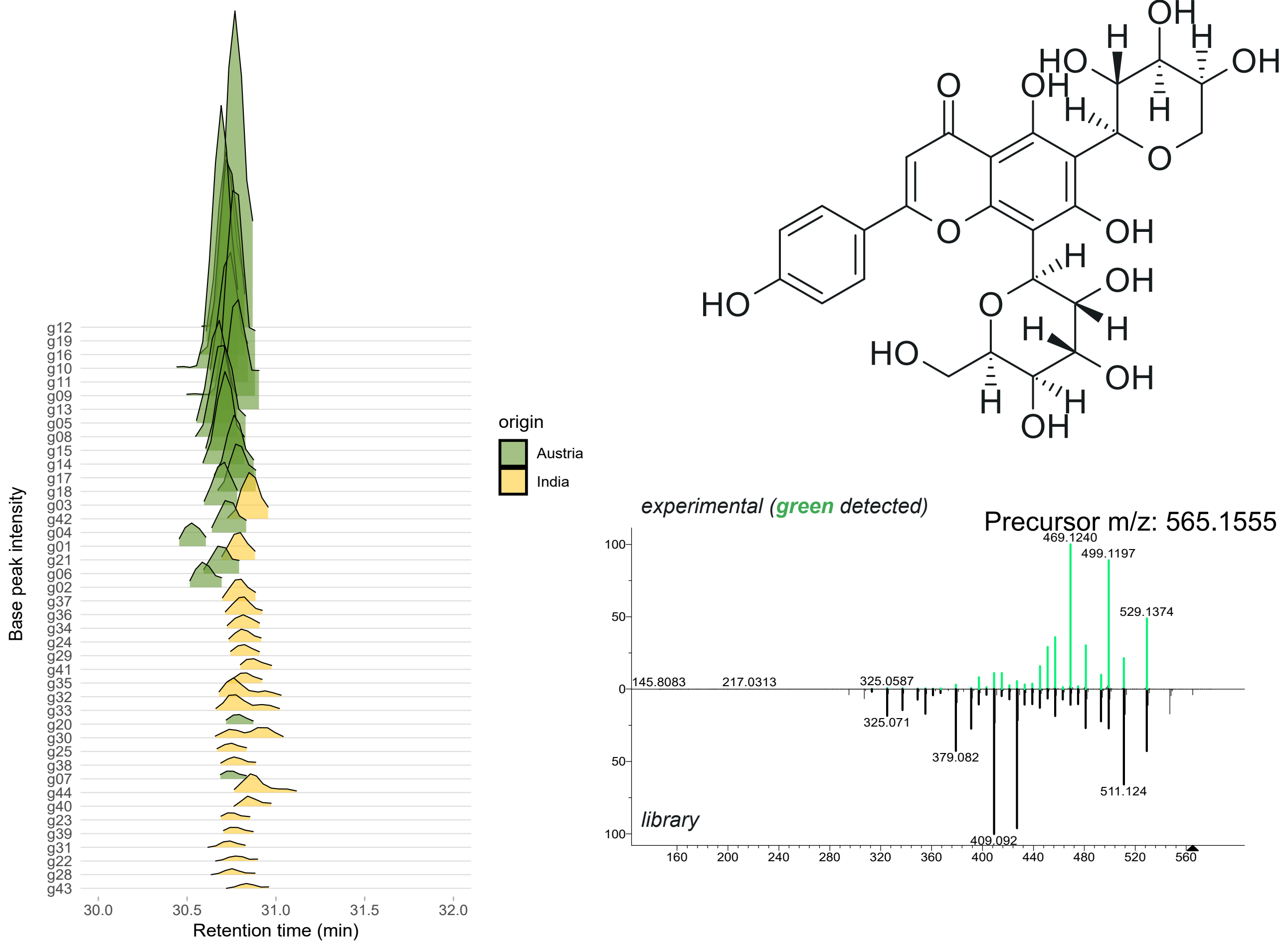

Schaftoside

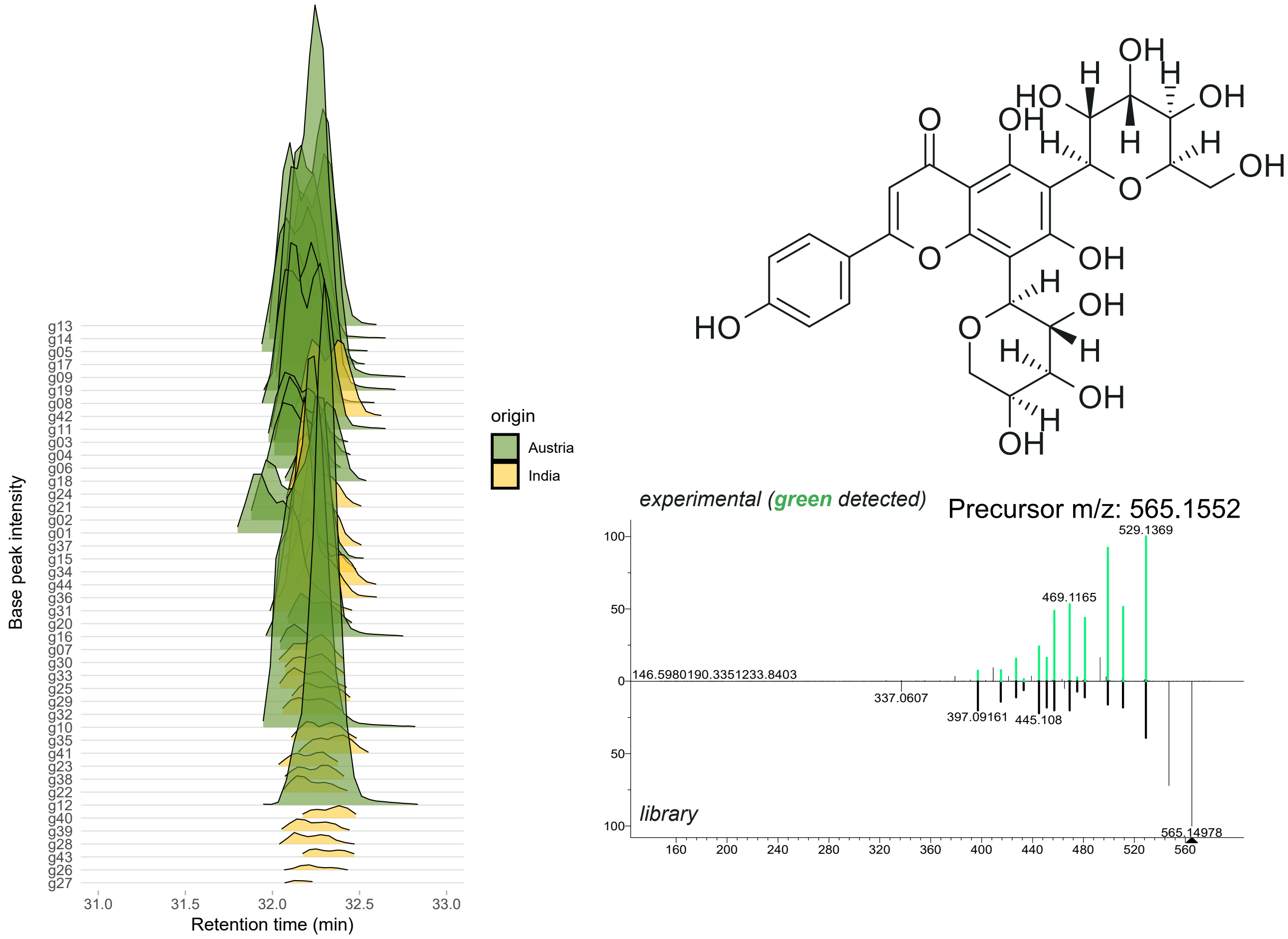

2−Hydroxybenzenepropanoic acid

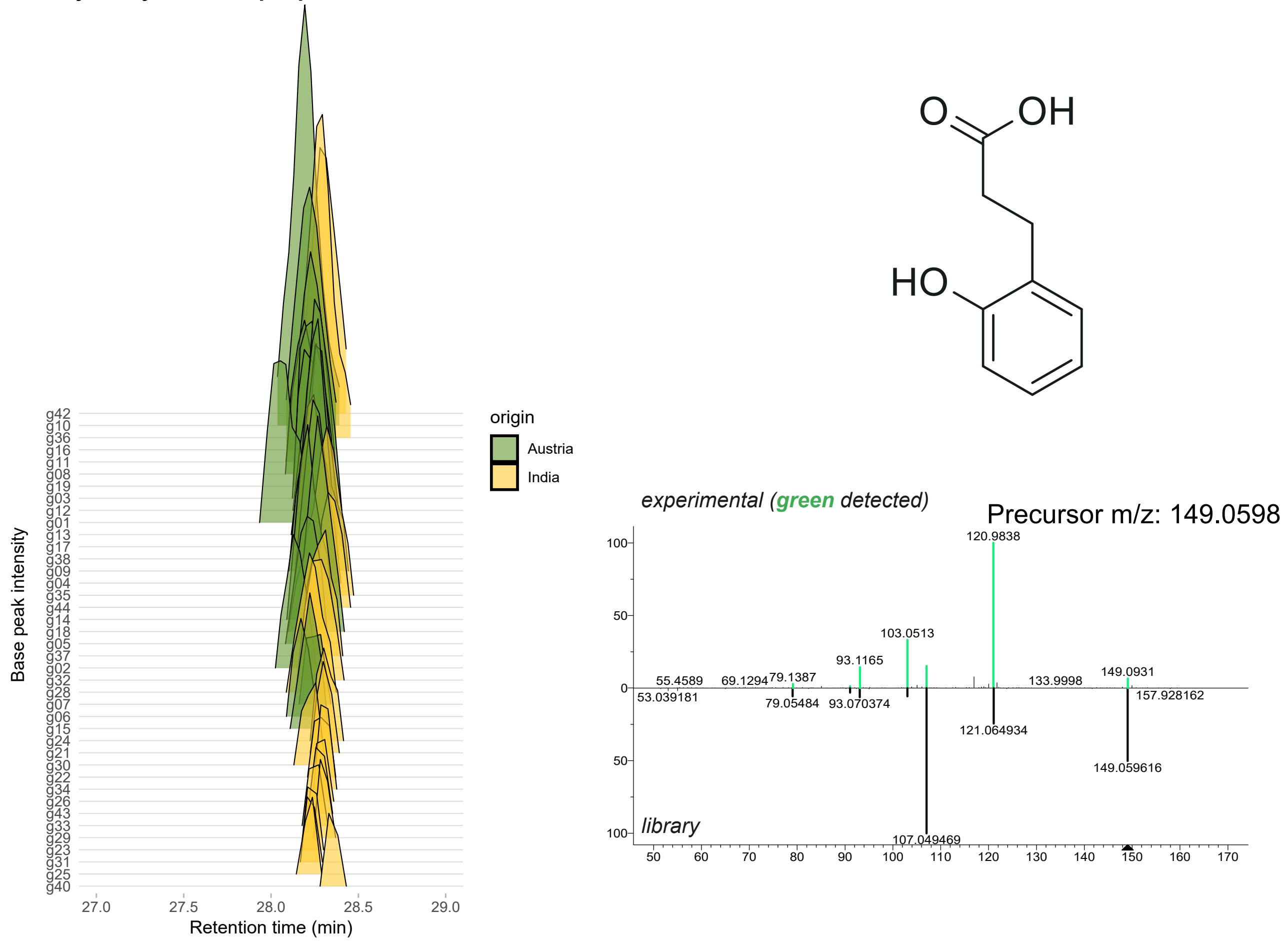

Isatin

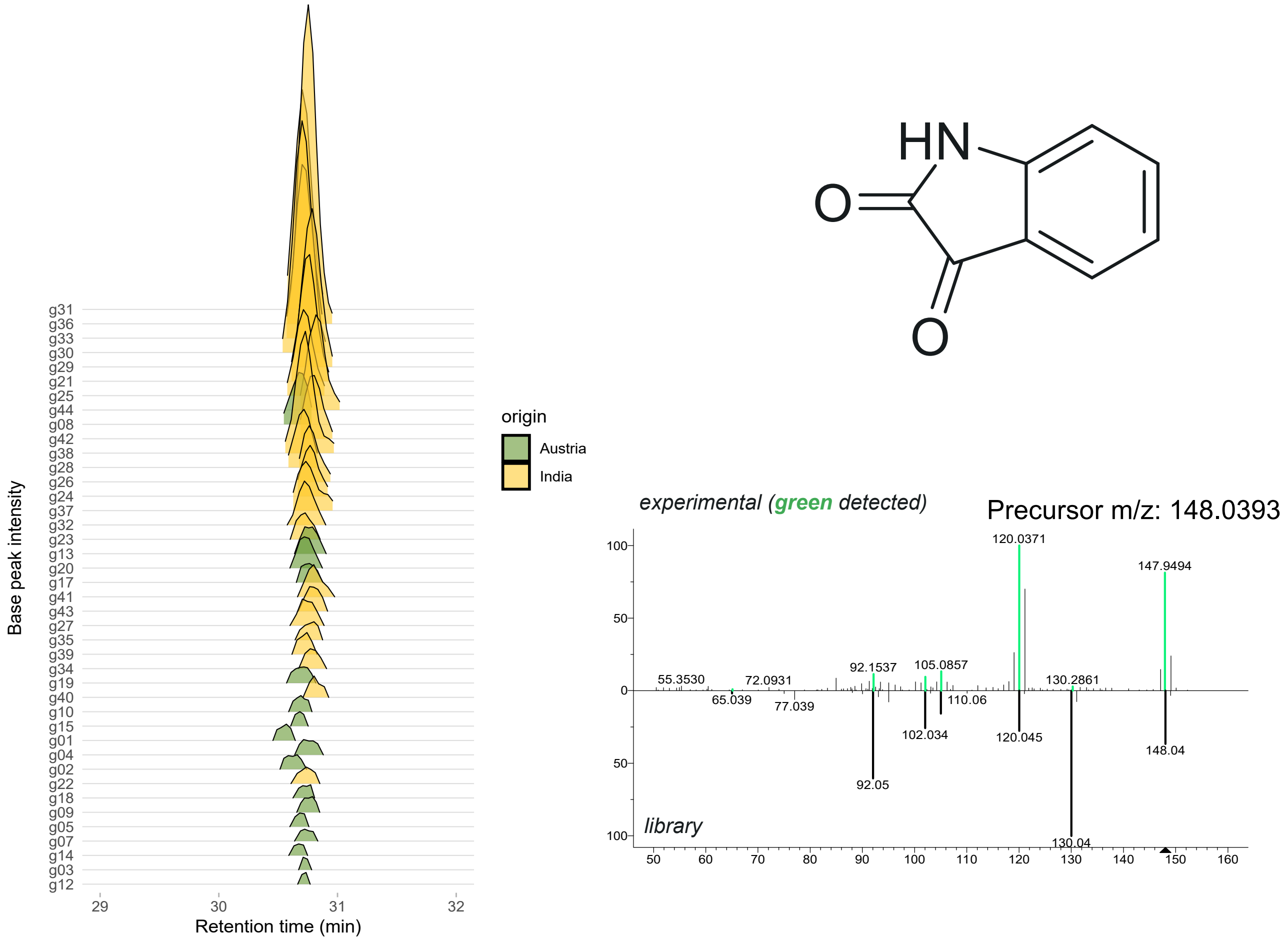

Zerumboneoxide

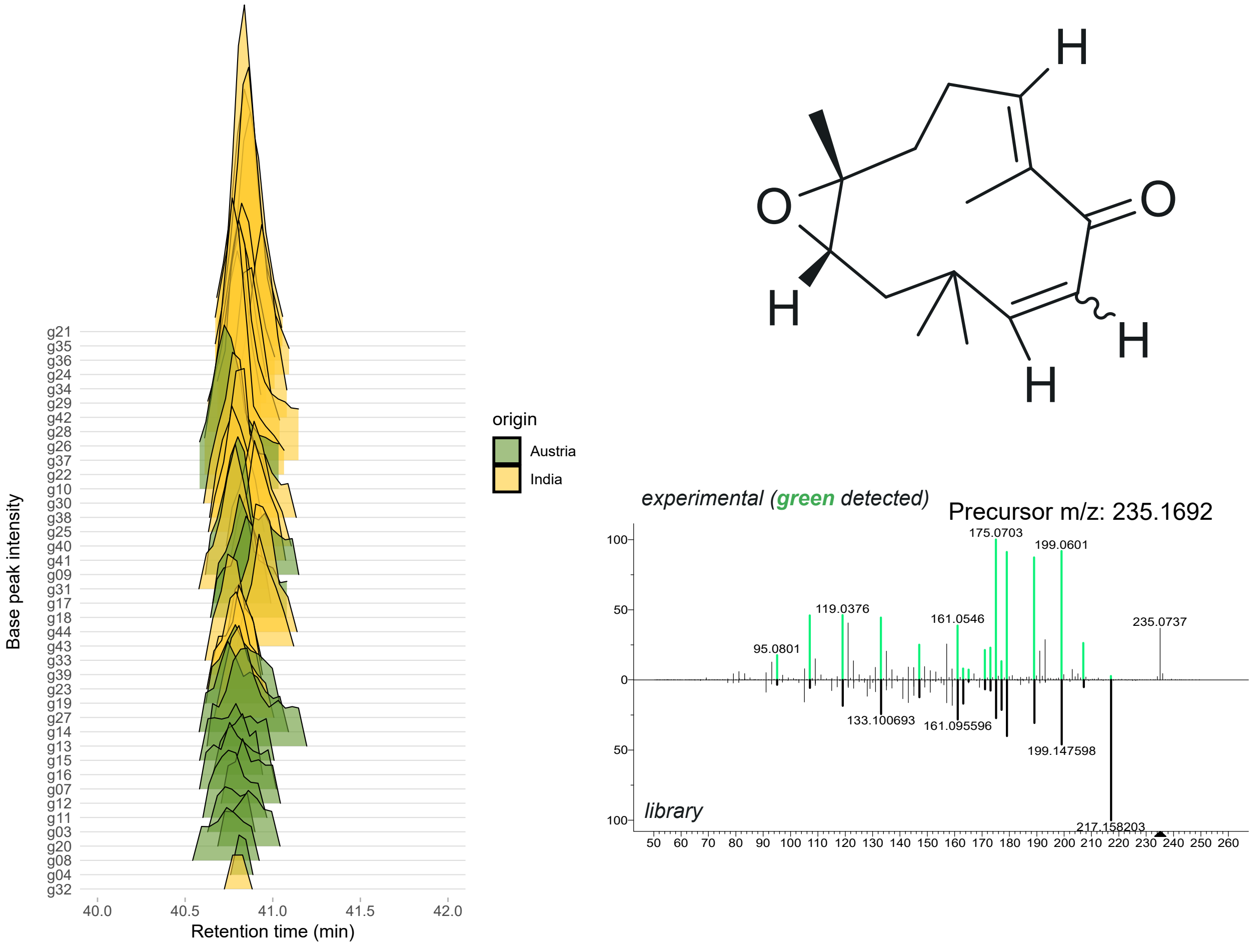

Lumichrome

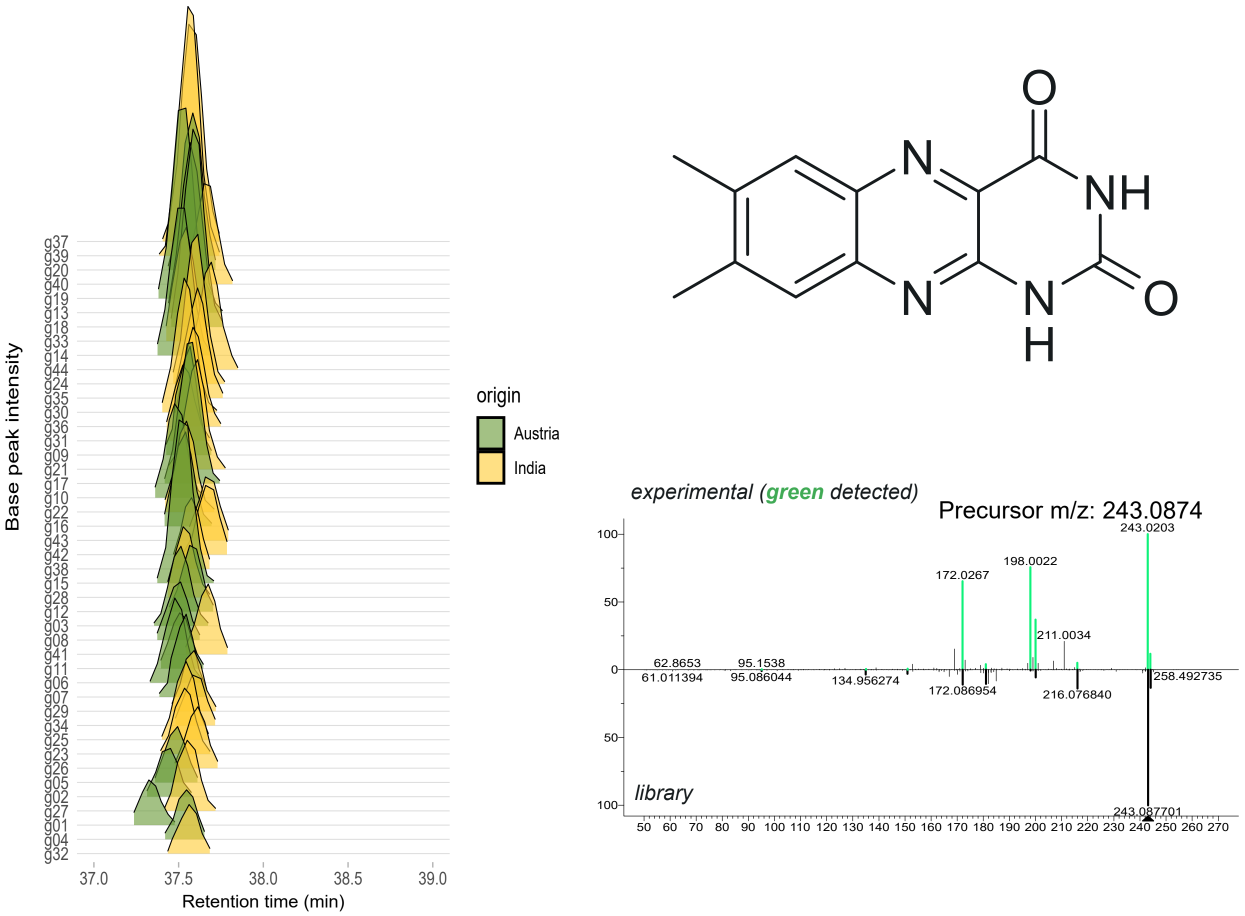

Perillic acid

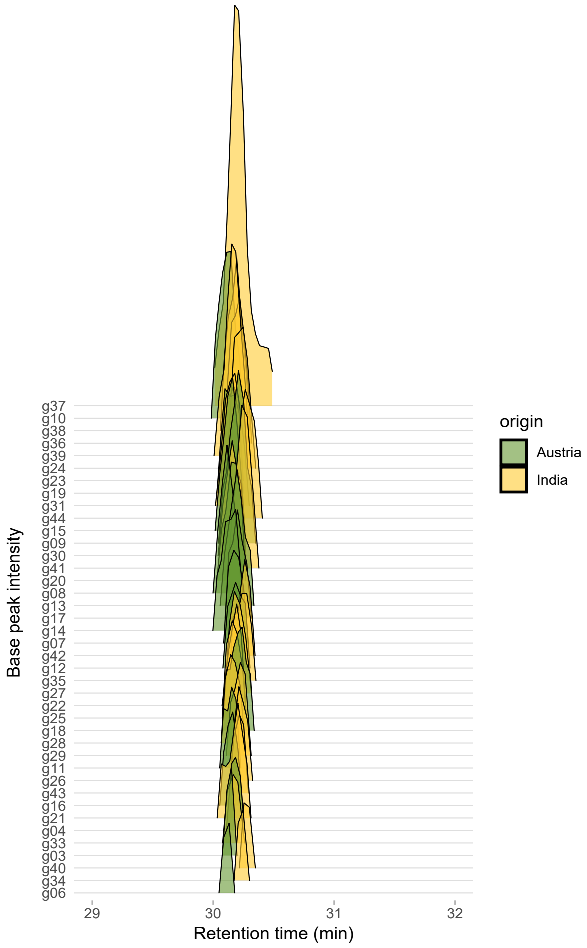

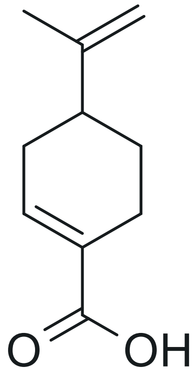

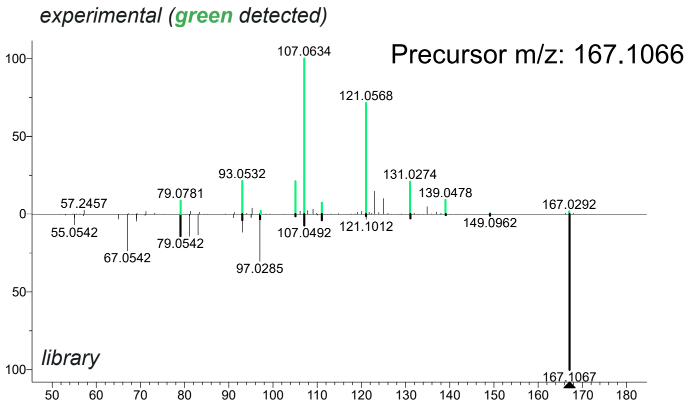

Syringic acid

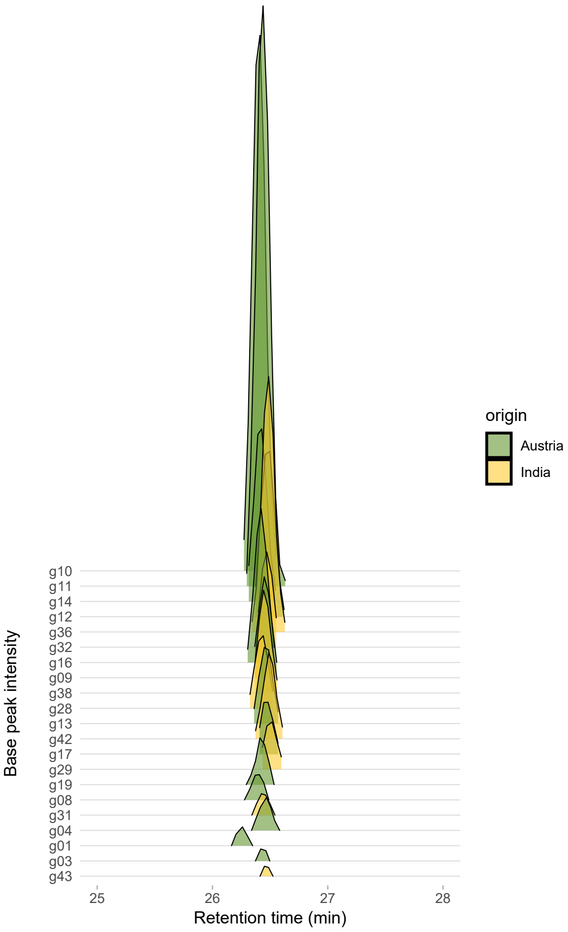

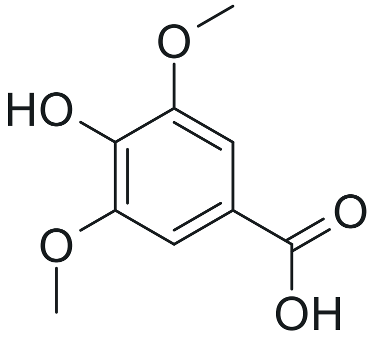

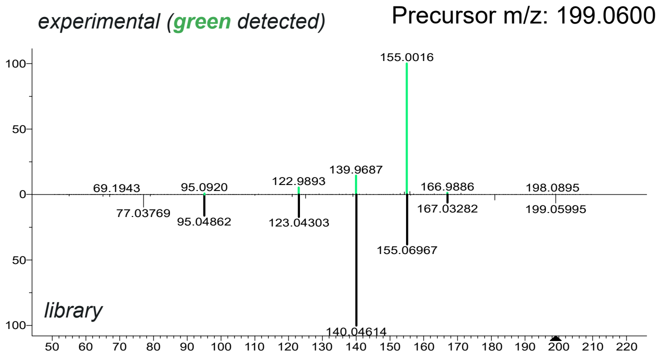

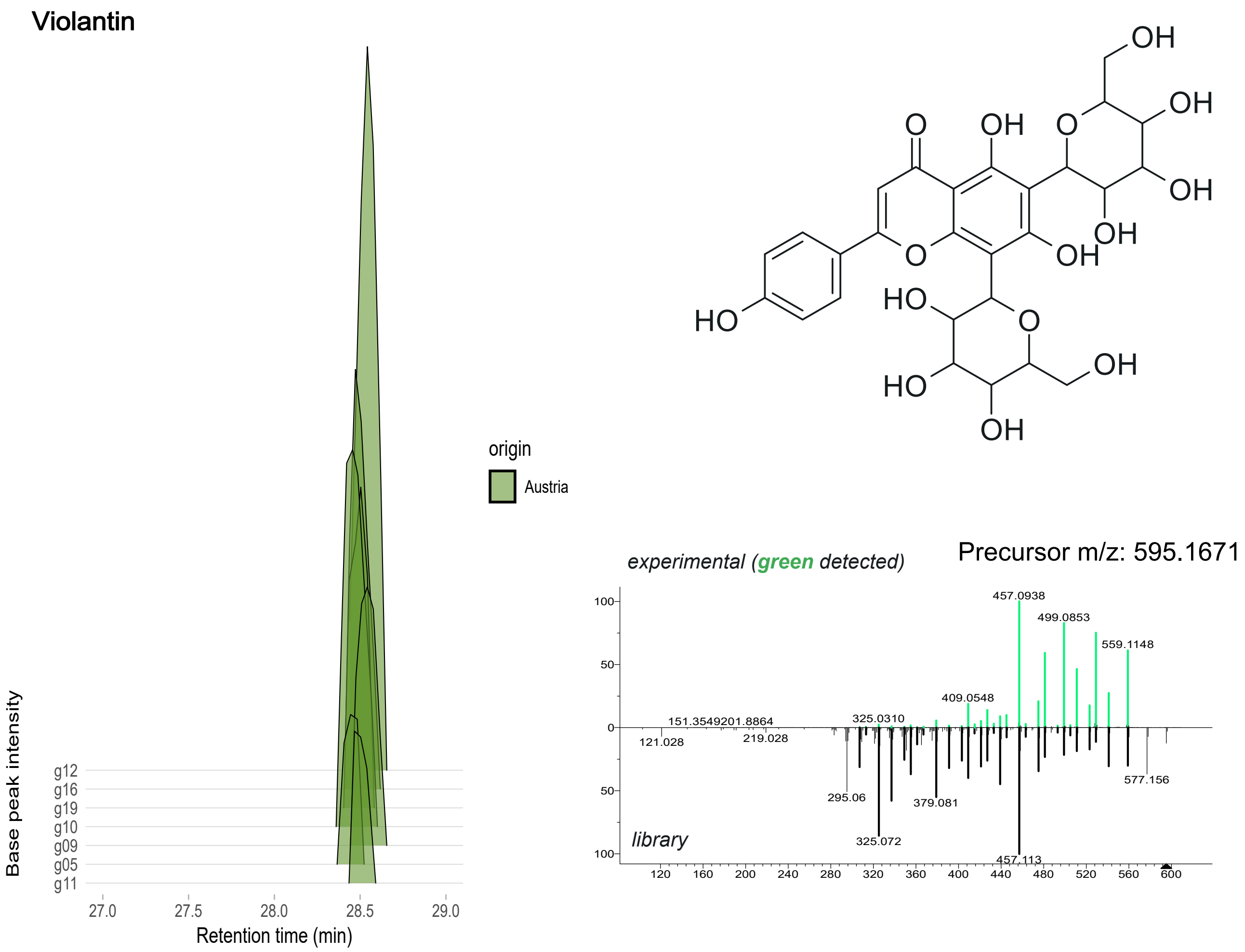

Violantin
Base peak intensity
g12
g16
g19
g10
g09
g05
g11
27.0
27.5
28.0
28.5
29.0
Retention time (min)
origin
Austria
experimental (green detected)
Precursor m/z: 595.1671
library
457.0938
499.0853
559.1148
409.0548
151.3549201.8864
325.0310
121.028
219.028
295.06
379.081
577.156
325.072
457.113

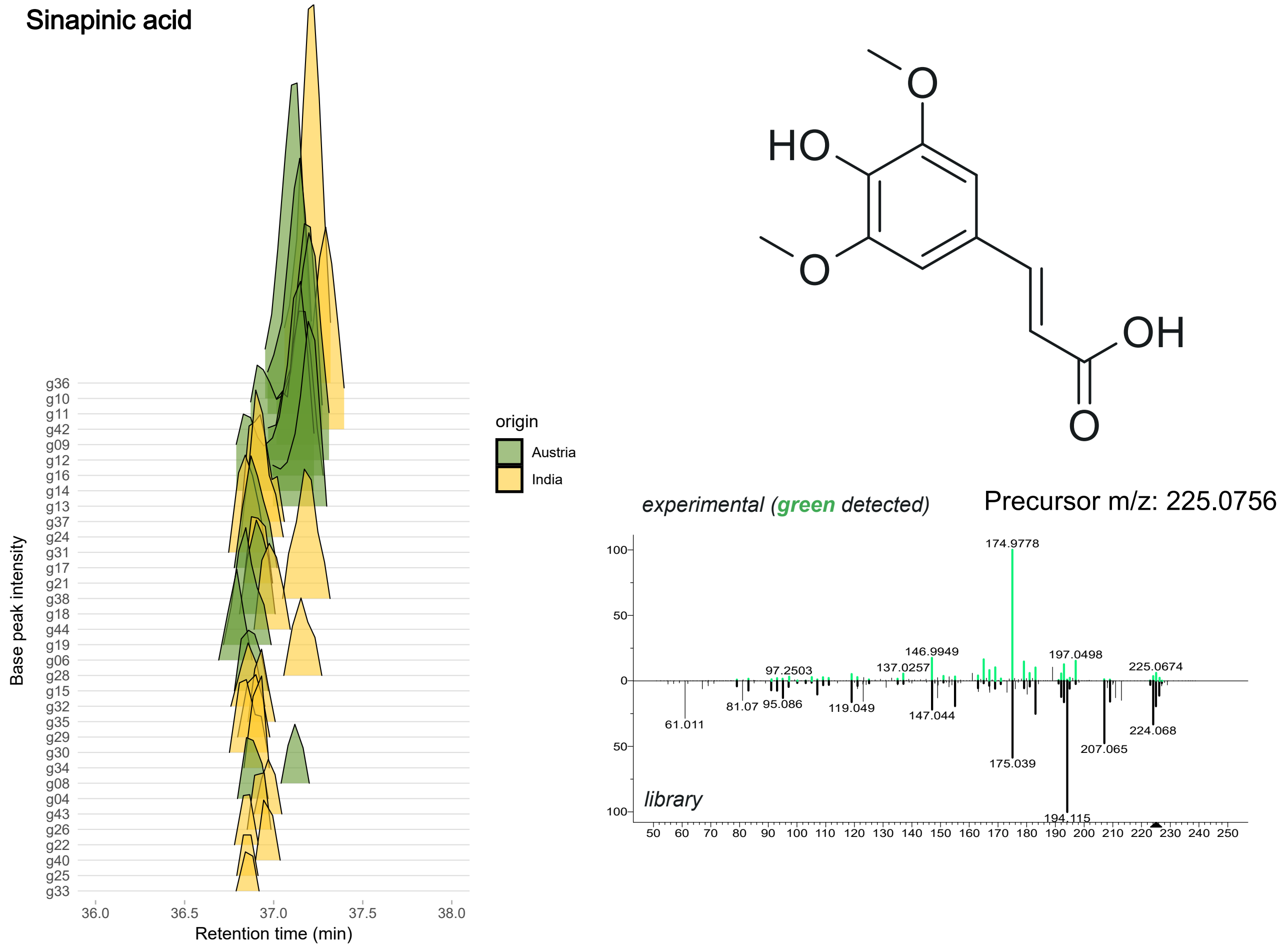

Sinapinic acid
Base peak intensity
g36
g10
g11
g42
g09
g12
g16
g14
g13
g37
g24
g31
g17
g21
g38
g18
g44
g19
g06
g28
g15
g32
g35
g29
g30
g34
g08
g04
g43
g26
g22
g40
g25
g33
36.0
36.5
37.0
37.5
38.0
Retention time (min)
origin
Austria
India
experimental (green detected)
Precursor m/z: 225.0756
library
174.9778
146.9949
197.0498
97.2503
137.0257
225.0674
61.011
81.07
95.086
119.049
147.044
224.068
207.065
175.039
194.115

Oleamide

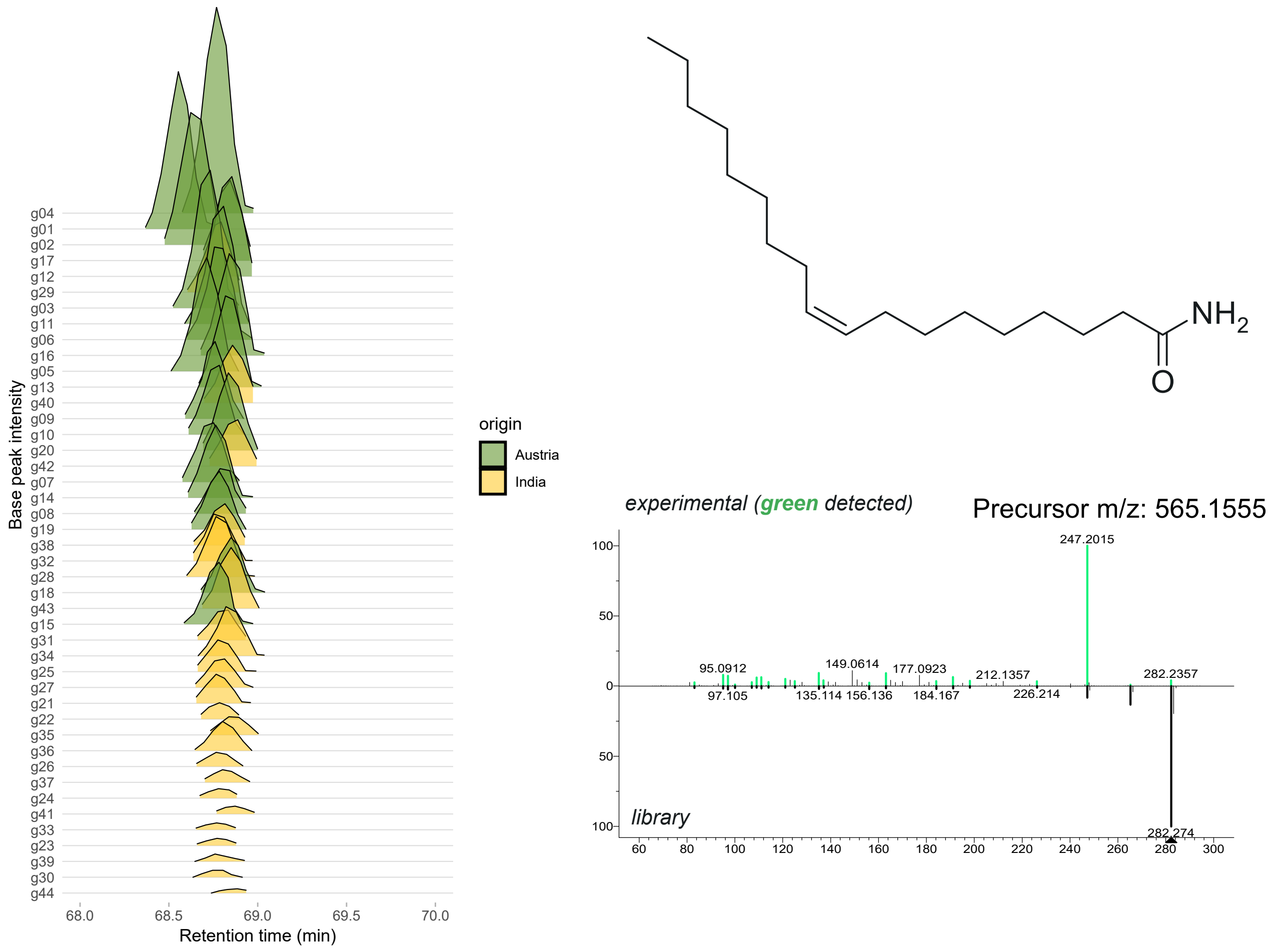

Oleamide

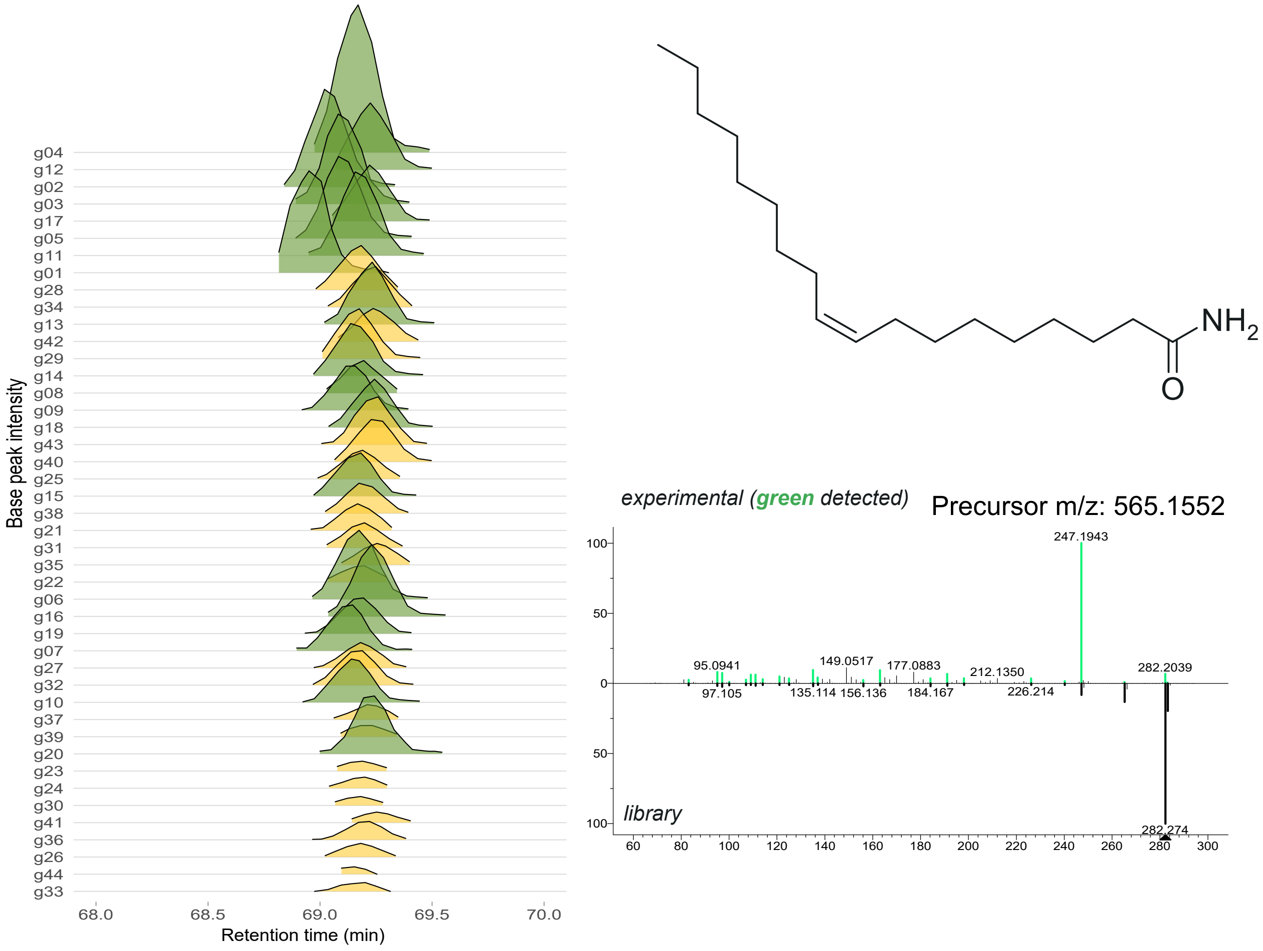

Cyclo(proline−leucine)

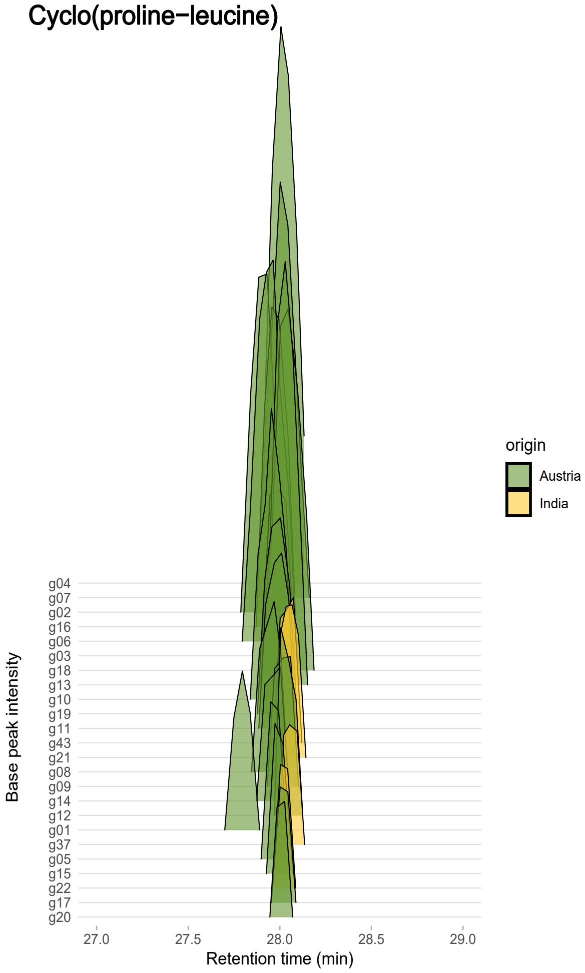

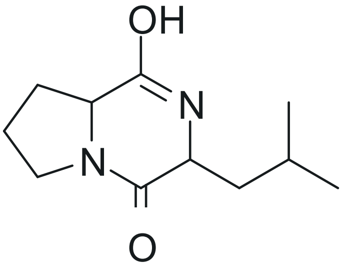

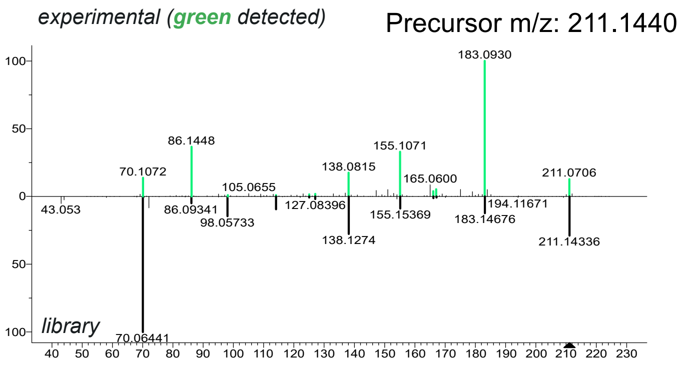

Cyclo(proline−leucine)

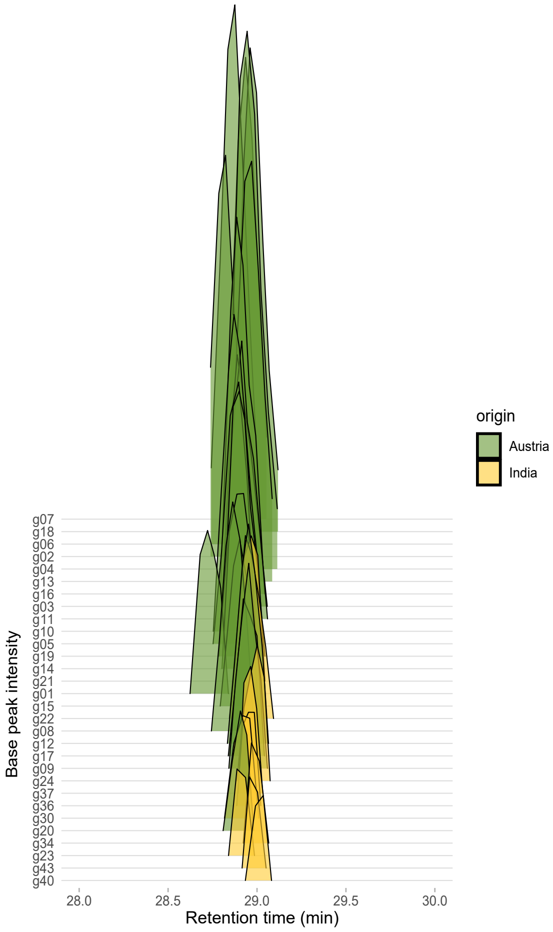

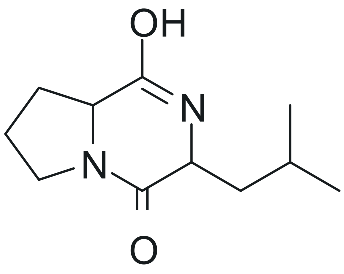

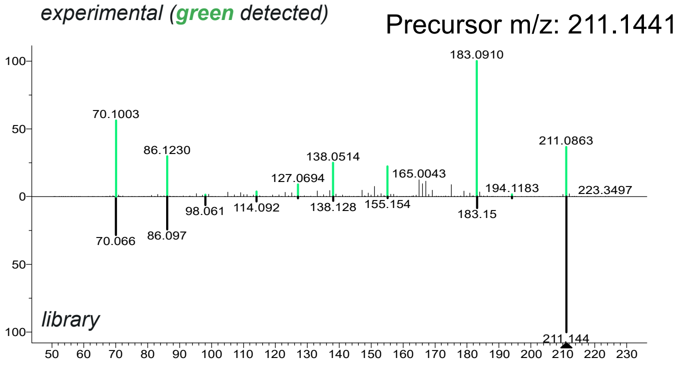

2−Acetylpyrazine

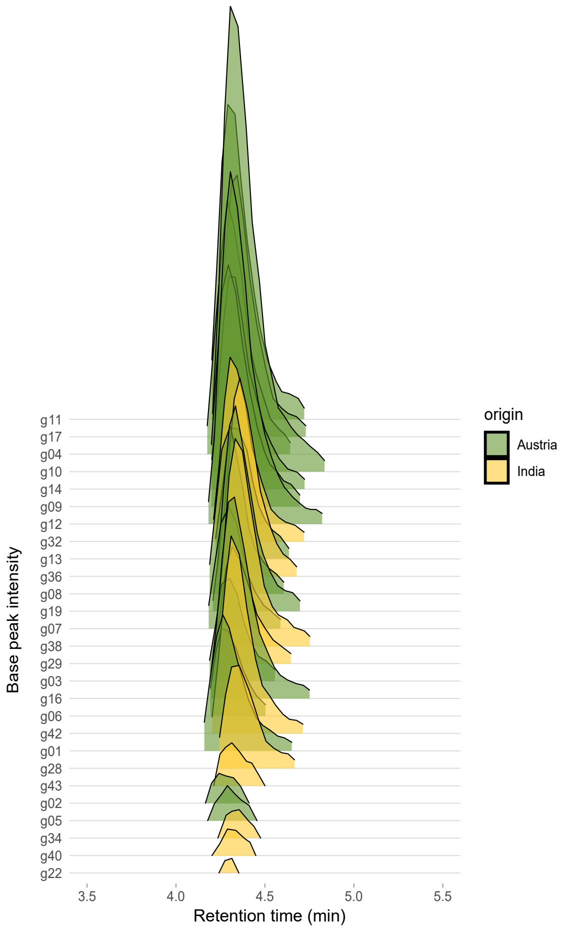

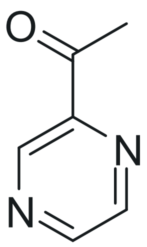

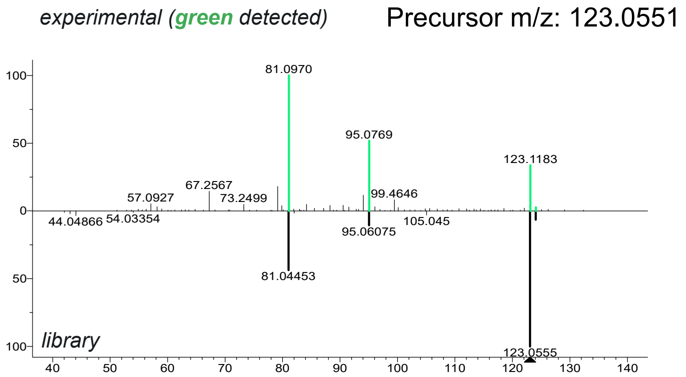

N,N−Dimethyl−L−arginine

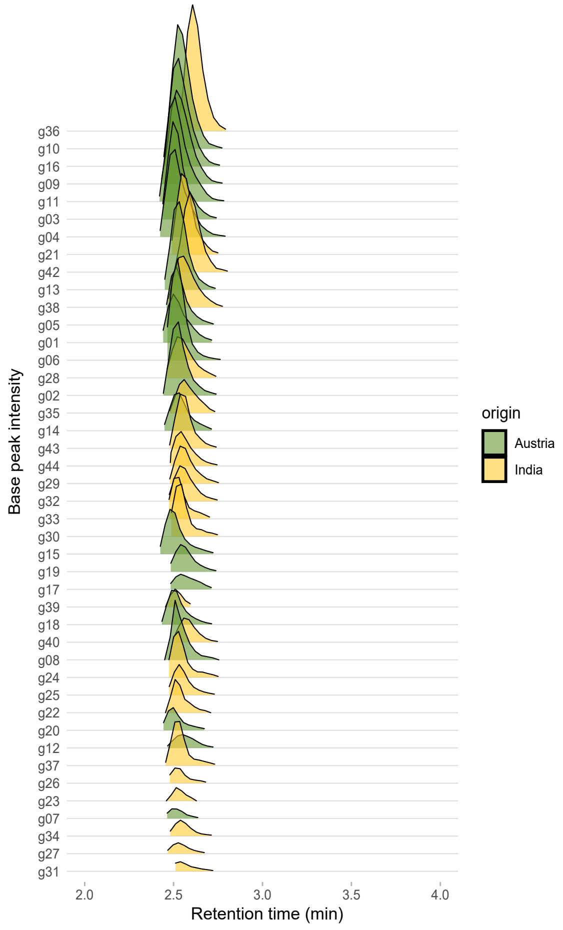

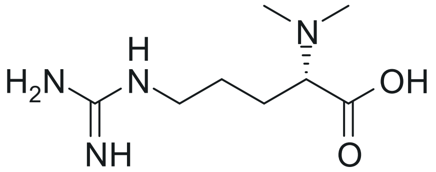

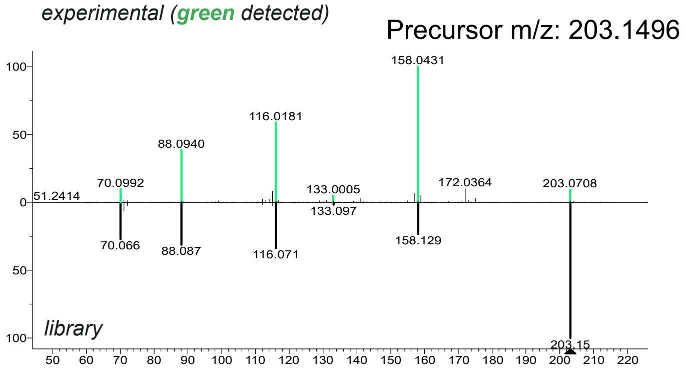

Ala−Ala

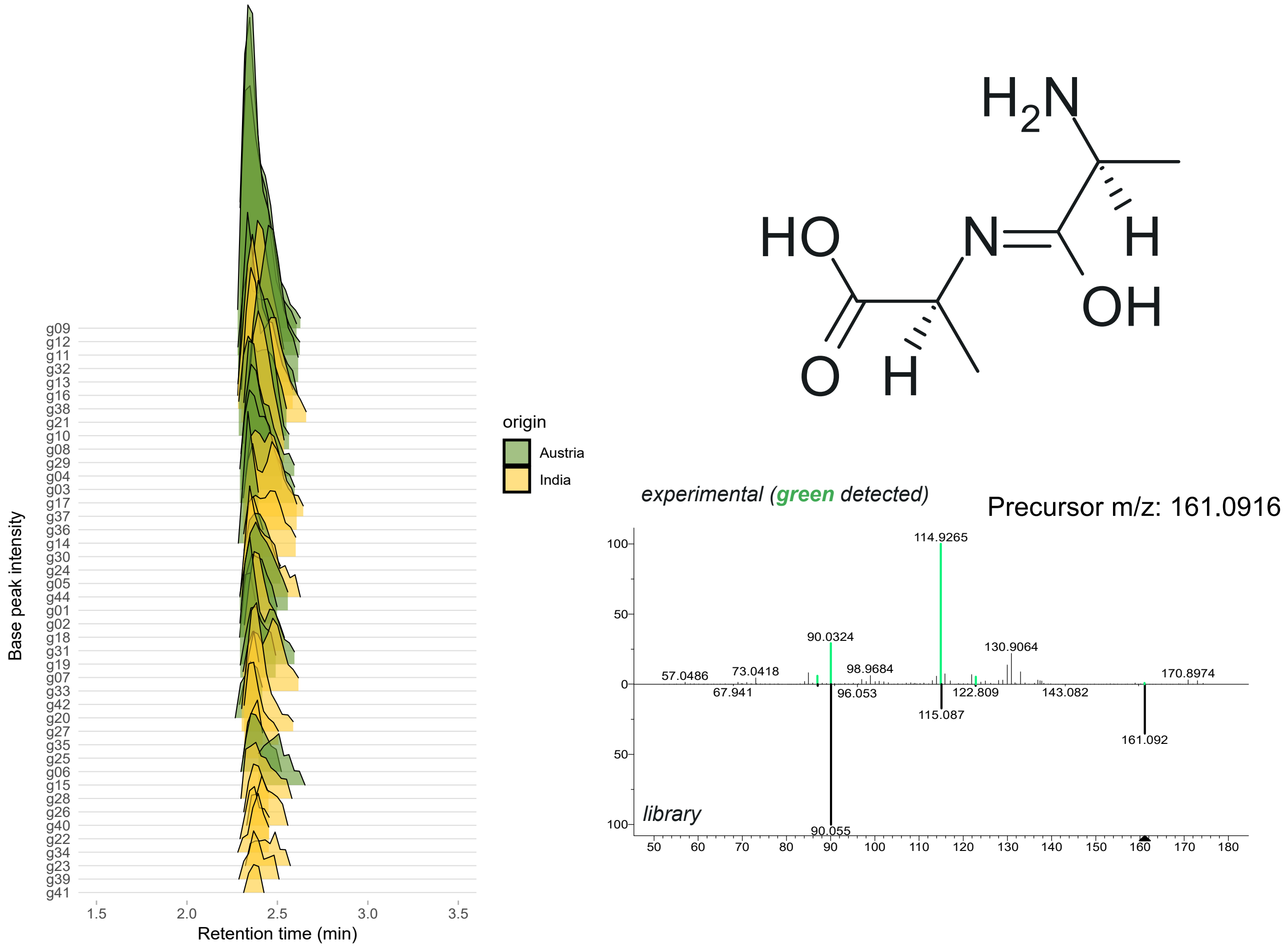

Caffeic acid

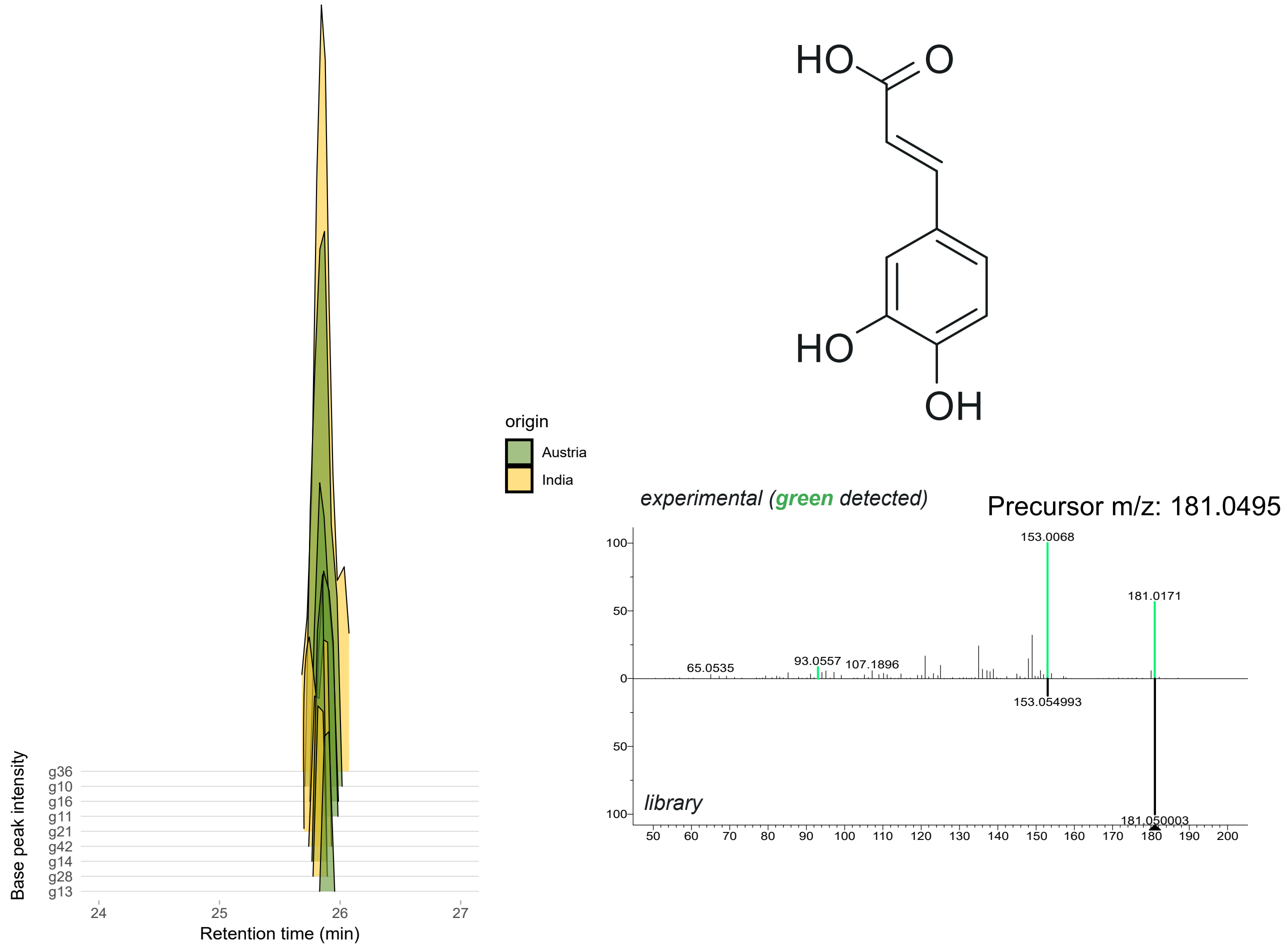

Carnitine

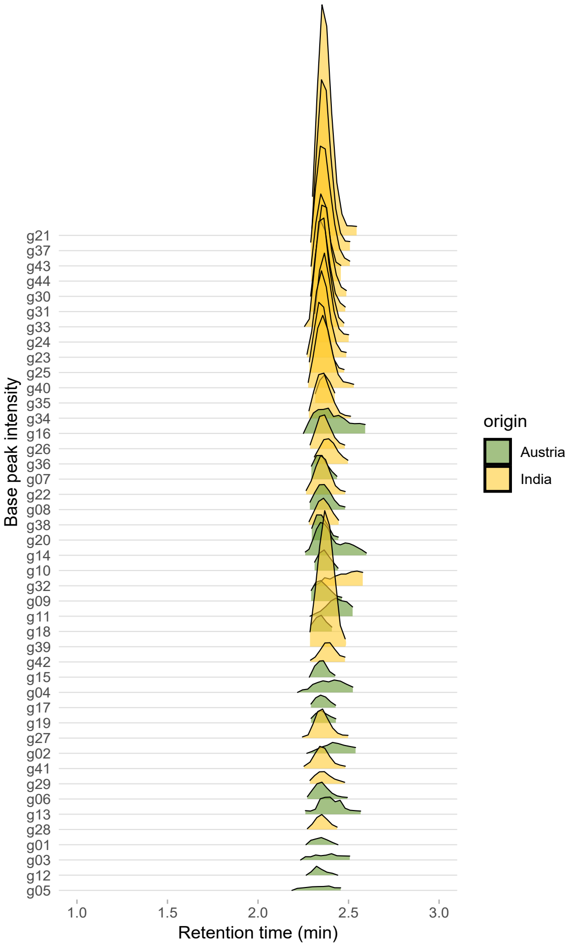

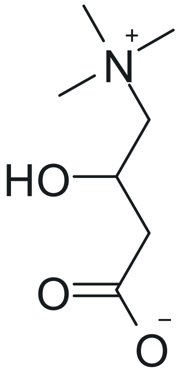

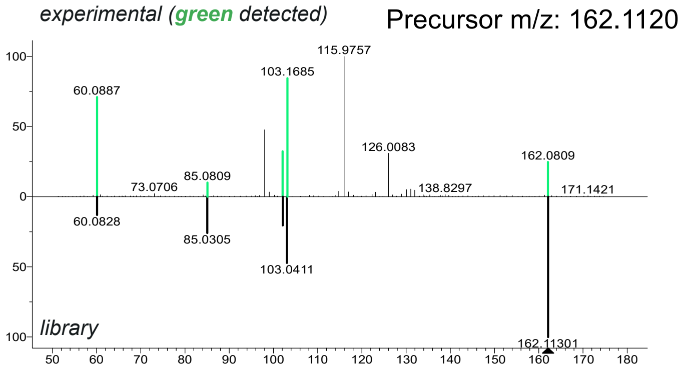

acetyl-DL-carnitine

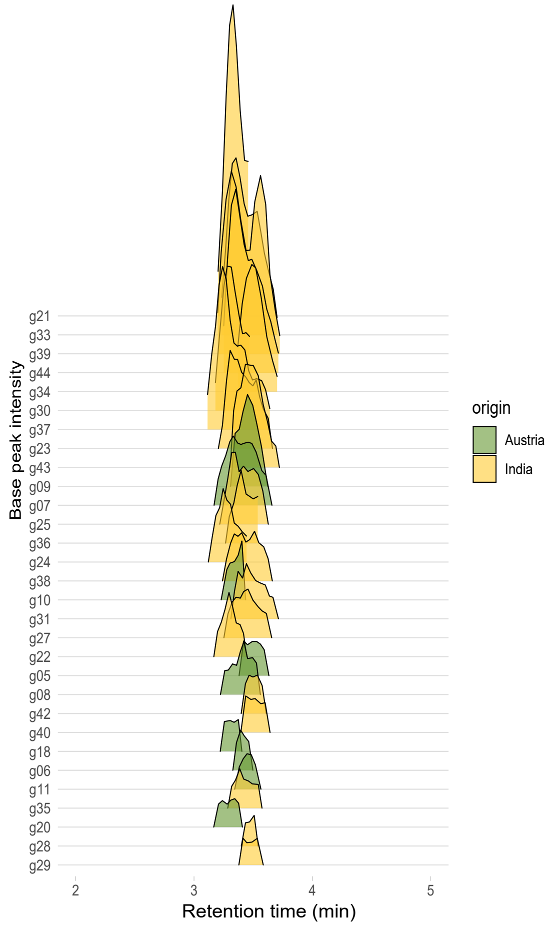

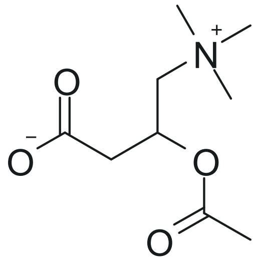

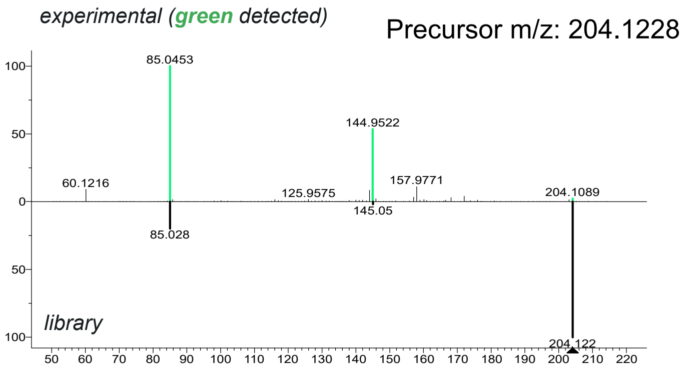

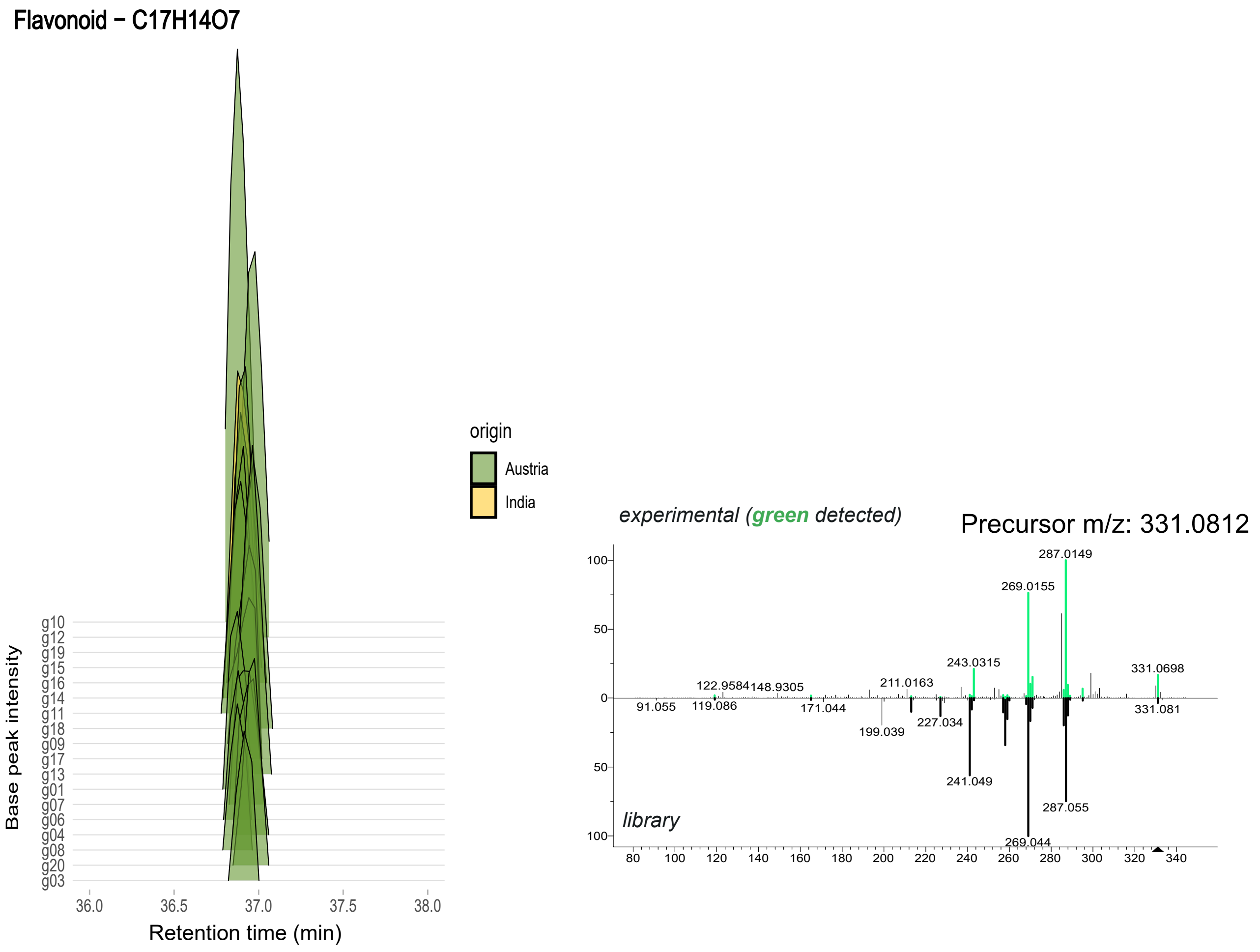
Flavonoid – C17H14O7
origin
Austria
India
Base peak intensity
Retention time (min)
experimental (green detected)
Precursor m/z: 331.0812
library
287.0149
269.0155
243.0315
331.0698
211.0163
122.9584
148.9305
91.055
119.086
171.044
199.039
227.034
241.049
287.055
269.044
331.081

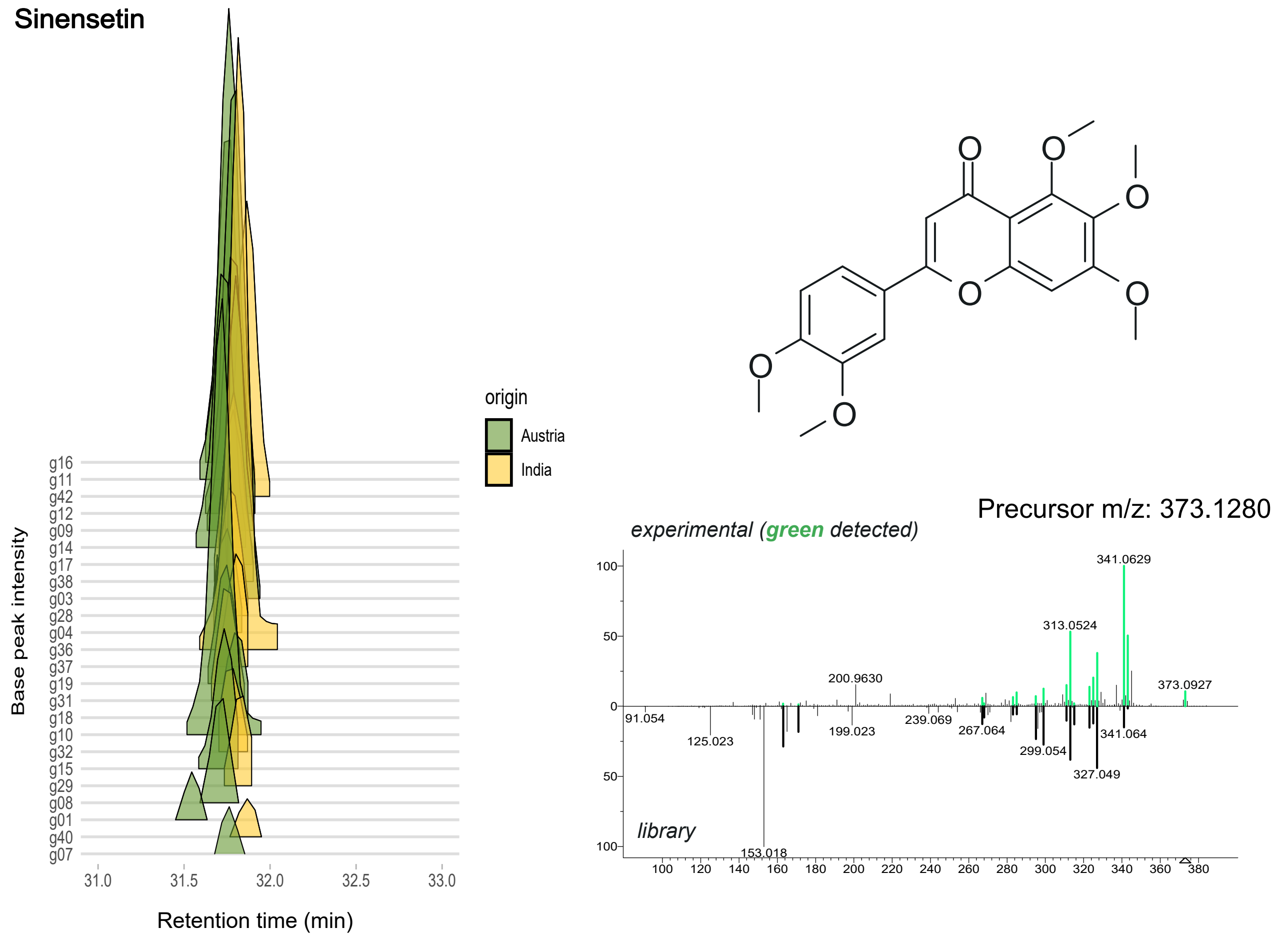
Sinensetin
origin
Austria
India
Base peak intensity
Retention time (min)
experimental (green detected)
Precursor m/z: 373.1280
library
341.0629
313.0524
200.9630
373.0927
91.054
125.023
199.023
239.069
267.064
299.054
341.064
327.049
153.018

Hydroferulic acid

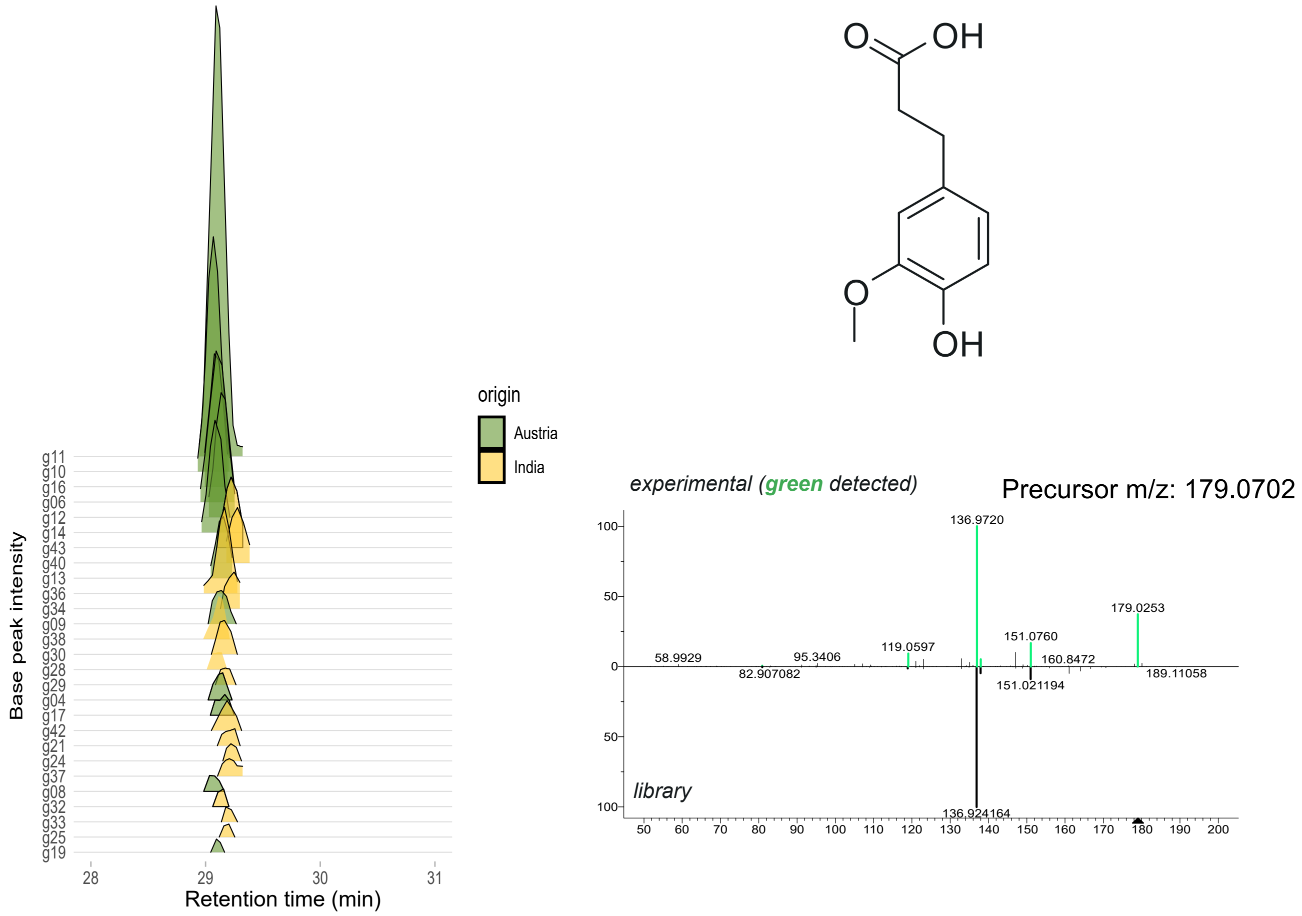

Flavone base + 3O, C−Pen, C−Pen

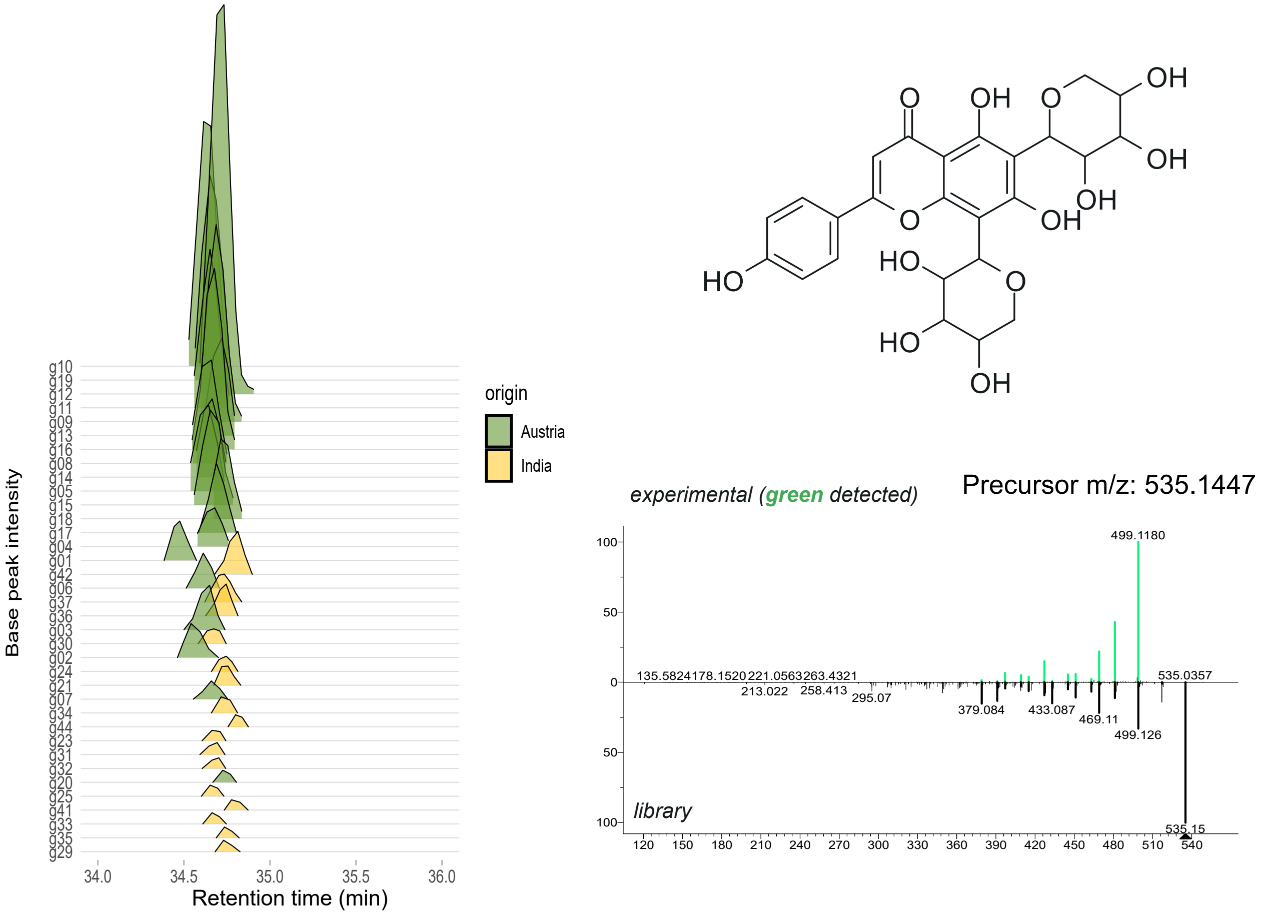

Supplement: Supplementary file 2 — Appendix S2 Chemical structure, chromatographic peak intensity and mass spectra of the significant metabolites. [file PBI-23-4755-s001.pdf]
