# Supplementary material for: Natural variation of the wheat root exudate metabolome and its influence on biological nitrification inhibition activity
Source: Plant Biotechnol J. 2025 Jul 21;23(11):4755–72. doi: 10.1111/pbi.70248 (PMC12576471; doi:10.1111/pbi.70248)
Supplement: Supplementary file 3 — Figure S1 GC–MS metabolomics analysis. Primary metabolites identified in the root exudates of different wheat genotypes originated from Austria and India. (a) Venn diagram. (b) Bar plots of each chemical class of the metabolites. The height of the bar represents the sum of the abundance of metabolites from each origin (Austria and India). (c) Principal Component Analysis. Principal Component 1 (PC1) ranked the metabolites in the root exudates. Loadings of PC1 are shown in rows. The bar colour indicates the origin of the genotypes (Austria and India), in which each metabolite is median. Figure S2 LC–MS metabolomics analysis. Secondary metabolites identified in the root exudates of different wheat genotypes originated from Austria and India. (a) Principal Component analysis. All detected features were used for PCA analysis. (b) Volcano plot. A volcano plot was generated with a 44‐genotype dataset (20 Austrian genotypes and 24 Indian genotypes, and 6632 metabolic features). Green circles represent metabolites with a fold change ≥2 that were statistically significant (P ≤ 0.01), indicating higher accumulation in Austrian genotypes than in Indian genotypes. Yellow circles represent metabolites with a fold change ≤ −2 that was statistically significant (P ≤ 0.01), indicating higher accumulation in Indian genotypes than in Austrian genotypes. Grey circles represent metabolites with a fold change ≤2 to ≥ −2 and lacking statistical significance (P > 0.01). (c) Upset plot. Three hundred and fifty‐seven annotated features (Annotation levels 1–4) were used for the upset diagram. (d, e) Principal Component Analysis of 357 annotated features (Annotation level 1–4). PC1 and PC2 are top‐ranked metabolites in root exudates. The highest loadings (15 highest and 15 lowest) of PC1 and PC2 are shown in rows for each principal component. Bar colours indicate the origin of the genotypes (Austria and India) in which each metabolite is a median. Figure S3 Spearman correlation analysis was u [file PBI-23-4755-s004.pdf]

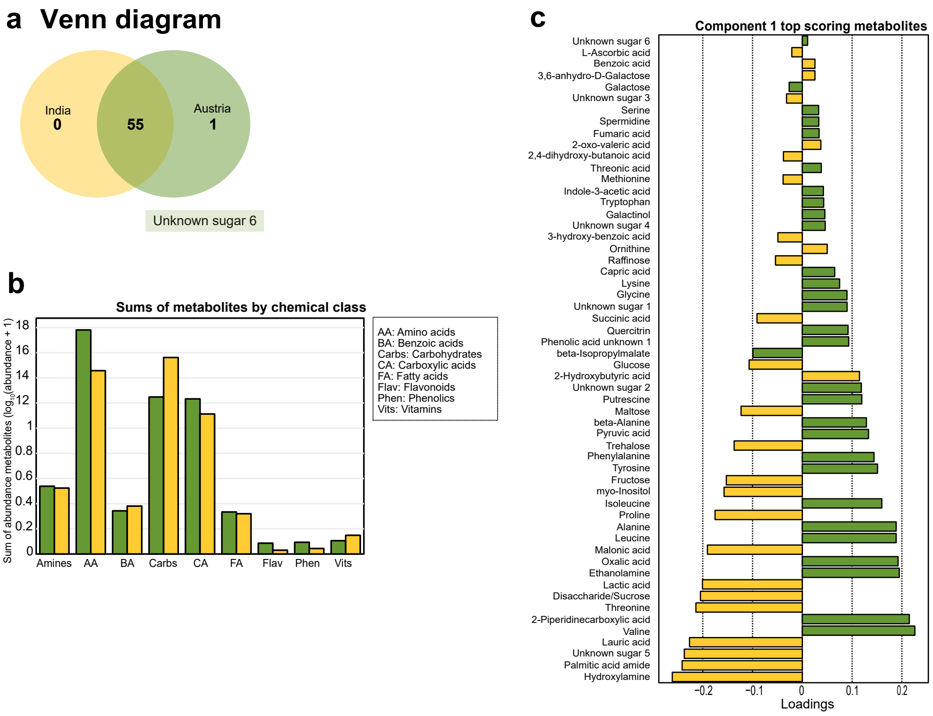

Figure S1.

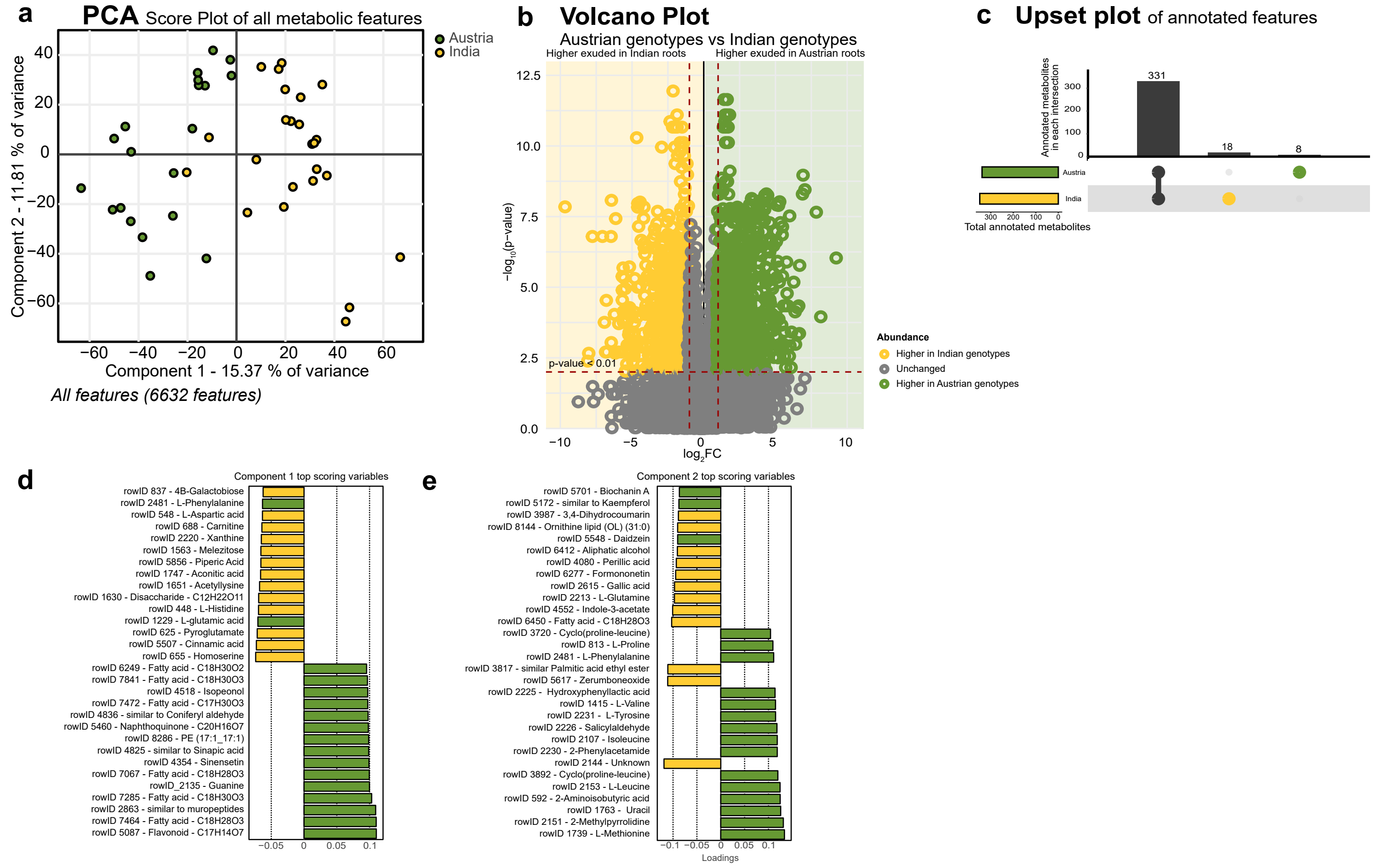

Figure S2.

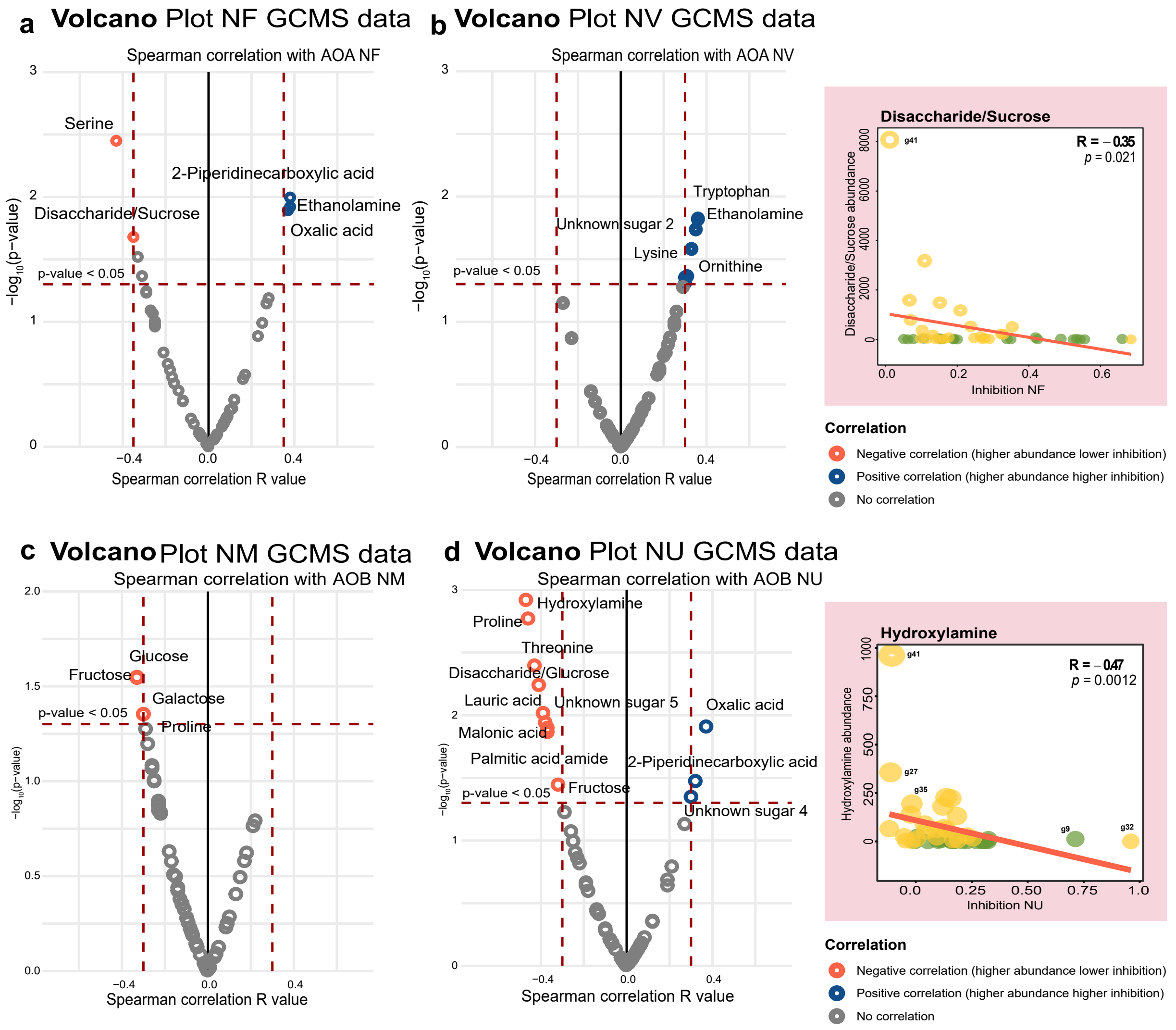
a Volcano Plot NF GCMS data
Spearman correlation with AOA NF
Serine
2-Piperidinecarboxylic acid
Ethanolamine
Disaccharide/Sucrose
Oxalic acid
p-value < 0.05
−log10(p−value)
Spearman correlation R value
b Volcano Plot NV GCMS data
Spearman correlation with AOA NV
Tryptophan
Ethanolamine
Unknown sugar 2
Lysine
Ornithine
p-value < 0.05
Spearman correlation R value
Disaccharide/Sucrose
R = −0.35
p = 0.021
Disaccharide/Sucrose abundance
Inhibition NF
Correlation
Negative correlation (higher abundance lower inhibition)
Positive correlation (higher abundance higher inhibition)
No correlation
c Volcano Plot NM GCMS data
Spearman correlation with AOB NM
Glucose
Fructose
Galactose
p-value < 0.05
Proline
Spearman correlation R value
d Volcano Plot NU GCMS data
Spearman correlation with AOB NU
Hydroxylamine
Proline
Threonine
Disaccharide/Glucrose
Lauric acid
Unknown sugar 5
Oxalic acid
Malonic acid
Palmitic acid amide
2-Piperidinecarboxylic acid
p-value < 0.05
Fructose
Unknown sugar 4
Spearman correlation R value
Hydroxylamine
R = −0.47
p = 0.0012
Hydroxylamine abundance
Inhibition NU
Correlation
Negative correlation (higher abundance lower inhibition)
Positive correlation (higher abundance higher inhibition)
No correlation

Figure S3.

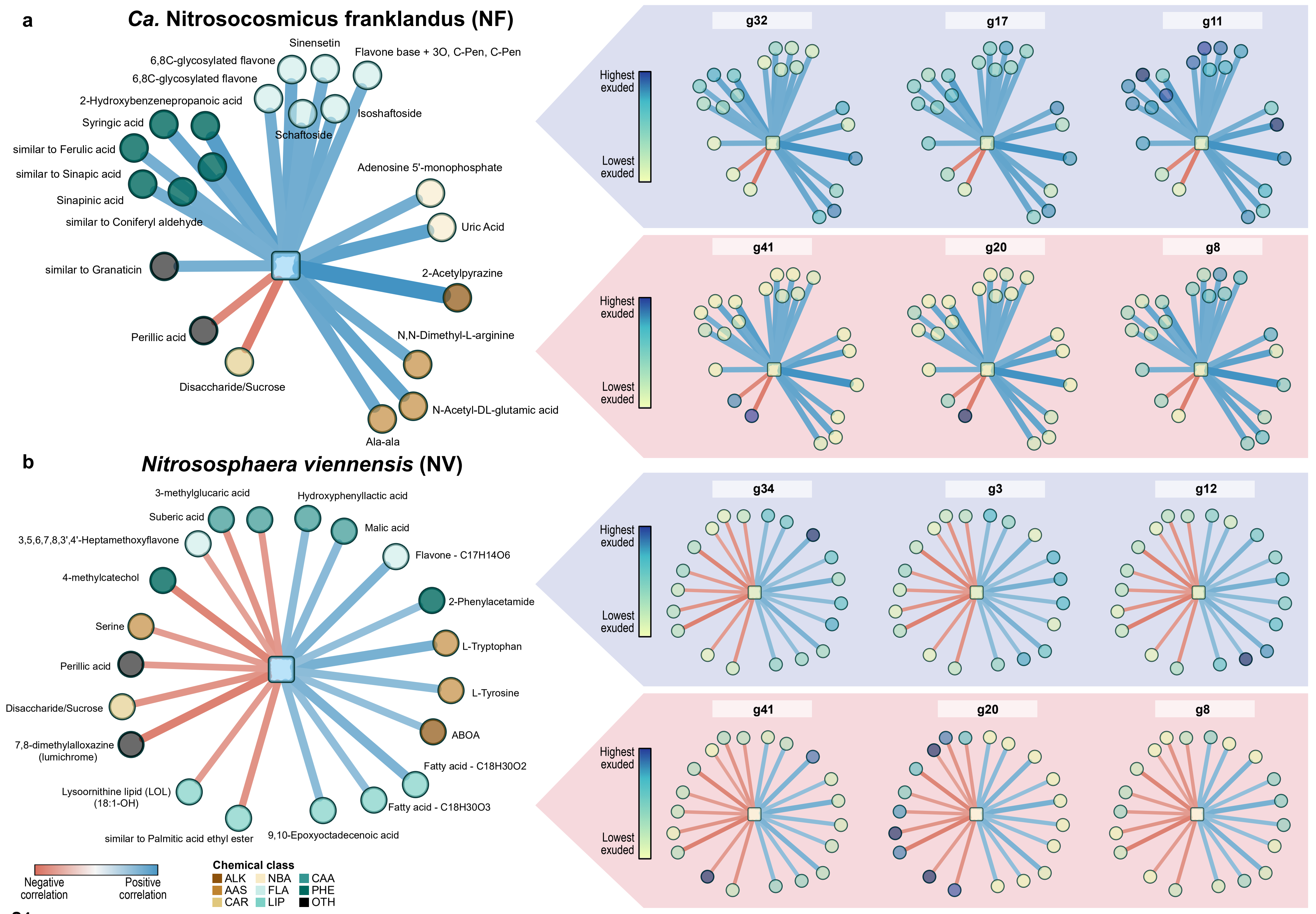
a
Ca. Nitrosocosmicus franklandus (NF)
Sinensetin
6,8C-glycosylated flavone
6,8C-glycosylated flavone
Flavone base + 3O, C-Pen, C-Pen
2-Hydroxybenzenepropanoic acid
Isoshaftoside
Syringic acid
Schaftoside
similar to Ferulic acid
similar to Sinapic acid
Sinapinic acid
similar to Coniferyl aldehyde
Adenosine 5'-monophosphate
Uric Acid
similar to Granaticin
2-Acetylpyrazine
Perillic acid
N,N-Dimethyl-L-arginine
Disaccharide/Sucrose
N-Acetyl-DL-glutamic acid
Ala-ala
g32
g17
g11
g41
g20
g8
Highest exuded
Lowest exuded
b
Nitrososphaera viennensis (NV)
3-methylglucaric acid
Hydroxyphenyllactic acid
Suberic acid
Malic acid
3,5,6,7,8,3',4'-Heptamethoxyflavone
Flavone - C17H14O6
4-methylcatechol
2-Phenylacetamide
Serine
L-Tryptophan
Perillic acid
L-Tyrosine
Disaccharide/Sucrose
ABOA
7,8-dimethylalloxazine (lumichrome)
Fatty acid - C18H30O2
Lysoornithine lipid (LOL) (18:1-OH)
Fatty acid - C18H30O3
similar to Palmitic acid ethyl ester
9,10-Epoxyoctadecenoic acid
g34
g3
g12
g41
g20
g8
Highest exuded
Lowest exuded
Negative correlation
Positive correlation
Chemical class
ALK
NBA
CAA
AAS
FLA
PHE
CAR
LIP
OTH

Figure S4.

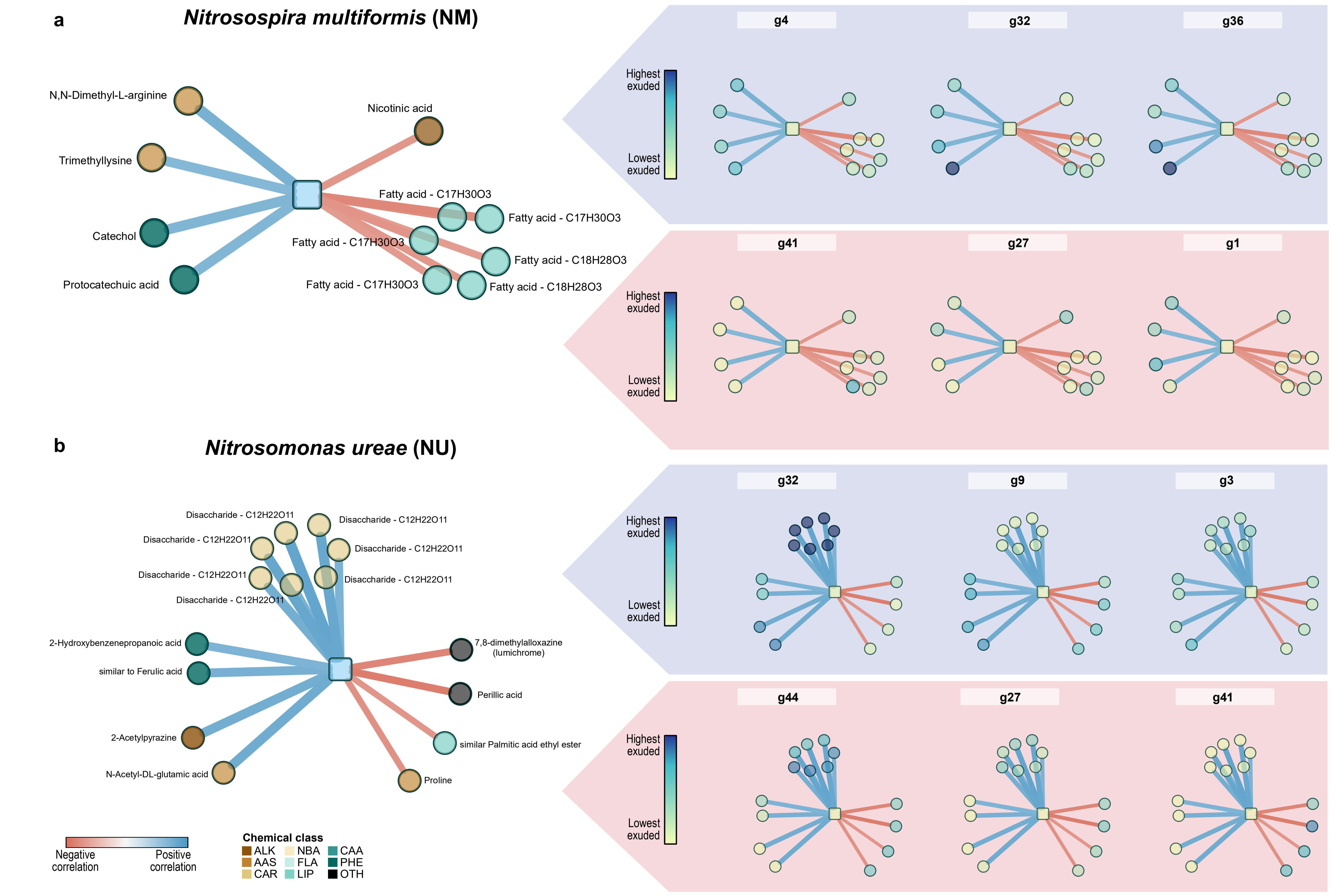
a
Nitrosospira multiformis (NM)
N,N-Dimethyl-L-arginine
Trimethyllysine
Catechol
Protocatechuic acid
Nicotinic acid
Fatty acid - C17H30O3
Fatty acid - C17H30O3
Fatty acid - C17H30O3
Fatty acid - C17H30O3
Fatty acid - C18H28O3
Fatty acid - C18H28O3
g4
g32
g36
g41
g27
g1
Highest exuded
Lowest exuded
b
Nitrosomonas ureae (NU)
Disaccharide - C12H22O11
Disaccharide - C12H22O11
Disaccharide - C12H22O11
Disaccharide - C12H22O11
Disaccharide - C12H22O11
Disaccharide - C12H22O11
Disaccharide - C12H22O11
2-Hydroxybenzenepropanoic acid
similar to Ferulic acid
2-Acetylpyrazine
N-Acetyl-DL-glutamic acid
7,8-dimethylalloxazine (lumichrome)
Perillic acid
similar Palmitic acid ethyl ester
Proline
g32
g9
g3
g44
g27
g41
Highest exuded
Lowest exuded
Negative correlation
Positive correlation
Chemical class
ALK
AAS
CAR
NBA
FLA
LIP
CAA
PHE
OTH

Figure S5.
